# Supplementary material for: Cut-and-Run: A Distinct Mechanism by which V(D)J Recombination Causes Genome Instability
Source: Mol Cell. 2019 May 2;74(3):584–597.e9. doi: 10.1016/j.molcel.2019.02.025 (PMC6509286; doi:10.1016/j.molcel.2019.02.025)
Supplement: Document S1. Figures S1–S7 and Tables S1–S6 [file mmc1.pdf]

Molecular Cell, Volume *74*

Supplemental Information

Cut-and-Run: A Distinct Mechanism by which V(D)J Recombination Causes Genome Instability

Christopher M. Kirkham, James N.F. Scott, Xiaoling Wang, Alastair L. Smith, Adam P. Kupinski, Anthony M. Ford, David R. Westhead, Peter G. Stockley, Roman Tuma, and Joan Boyes

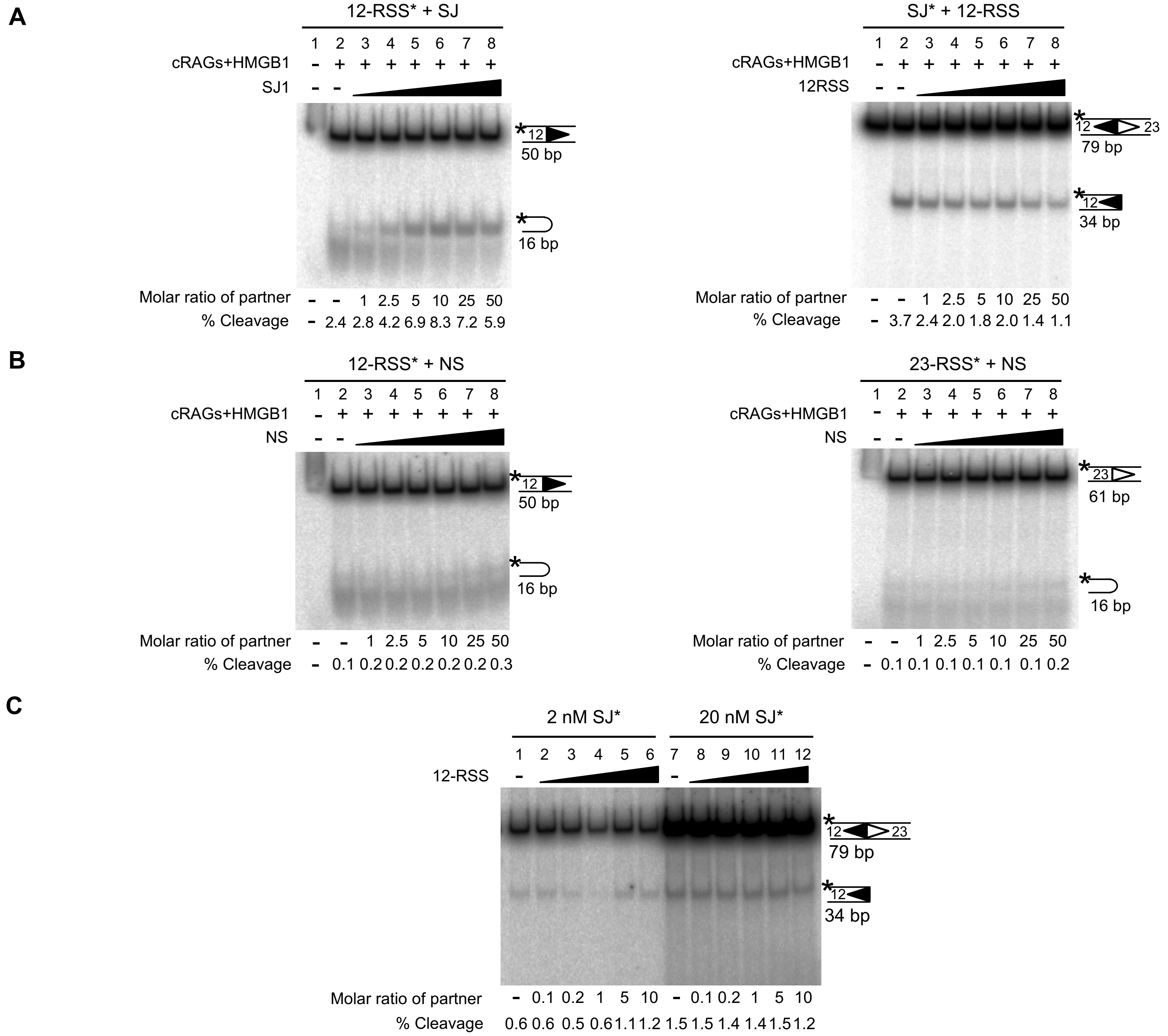

Supplementary Figure 1

Supplementary Figure Legends

Figure S1 – Asymmetric Cutting Occurs Under Different Conditions, Related to Figure 1. (A) Increasing amounts of unlabelled partner were added to an *in vitro* cleavage assay, as indicated by the filled arrow. This showed that i) cleavage is asymmetric over a wide range of partner ratios, and ii) that cleavage is greatest at a 10-fold excess of partner. The labelled oligonucleotide is indicated by an asterisk above each set of lanes. **(B)** Titration of a non-specific oligonucleotide does not enhance RAG cutting. Increasing amounts of an oligonucleotide that lacks RSS sequences (NS) were added to an *in vitro* cleavage assay, as indicated. **(C)** SJ cleavage by RAG proteins is not limited by the amount of substrate. The amount of labelled SJ was increased over a 10-fold range (lanes 1-6 versus 7-12) with no increase in per cent cutting. Titration of a partner RSS (indicated by the closed arrow) also did not increase the per cent cutting.

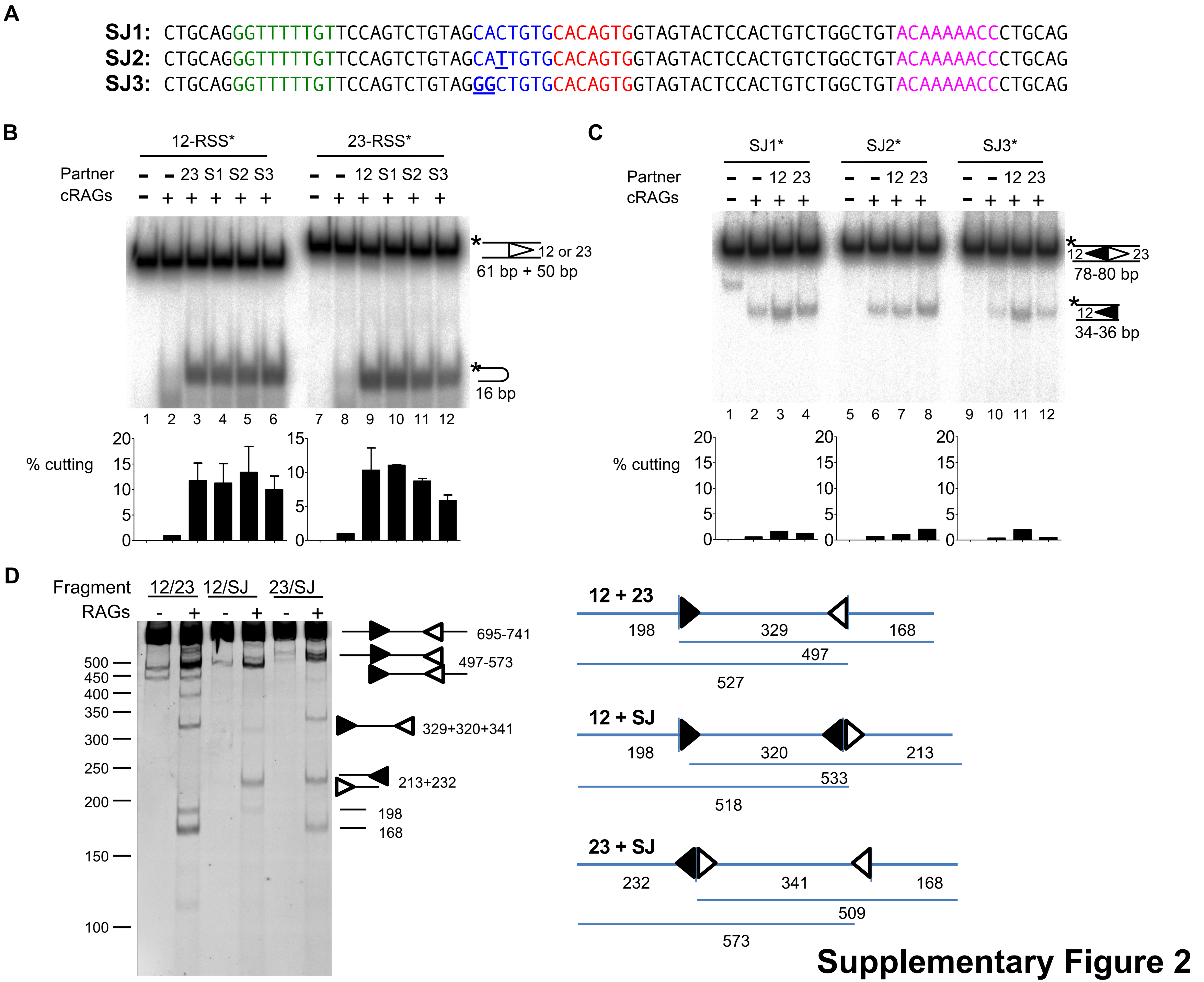

Supplementary Figure 2

Figure S2 – SJ cleavage is asymmetric with different SJ sequences, Related to Figure 1. (A) Comparison of the SJ sequences used. Bases that differ from consensus heptamer sequences are shown in bold. Blue = 12-RSS heptamer, green = 12-RSS nonamer, red = 23-RSS heptamer, magenta = 23-RSS nonamer. **(B)** SJs with common heptamer sequences (SJ2 and SJ3) were used in RAG cleavage reactions as partners for labelled 12 or 23-RSSs or **(C)** were radiolabelled and used as substrates with 12- and 23-RSS partners. Labelled oligonucleotides are indicated by an asterisk. See also Table S1 for frequently generated human ESCs. **(D)** The plasmid pJH290, which contains two RSSs 329 bp apart in a convergent orientation, was modified to contain a SJ in place of either the 12- or 23-RSS. Fragments with two complementary RSSs or an RSS plus SJ were PCR amplified from the plasmids, gel purified and used in RAG cutting assays (left). Following electrophoresis, DNA was stained with dsGreen. The expected product sizes for complete cleavage are shown to the right (12+23: 168+198+329 bp; 12+SJ: 198+213+320 bp; 23+SJ: 168+232+341 bp). Filled triangles = 12-RSS; open triangles = 23-RSS. The low level of cutting of the 12-RSS in pJH290 has been reported previously (Tevelev and Schatz, 2000).

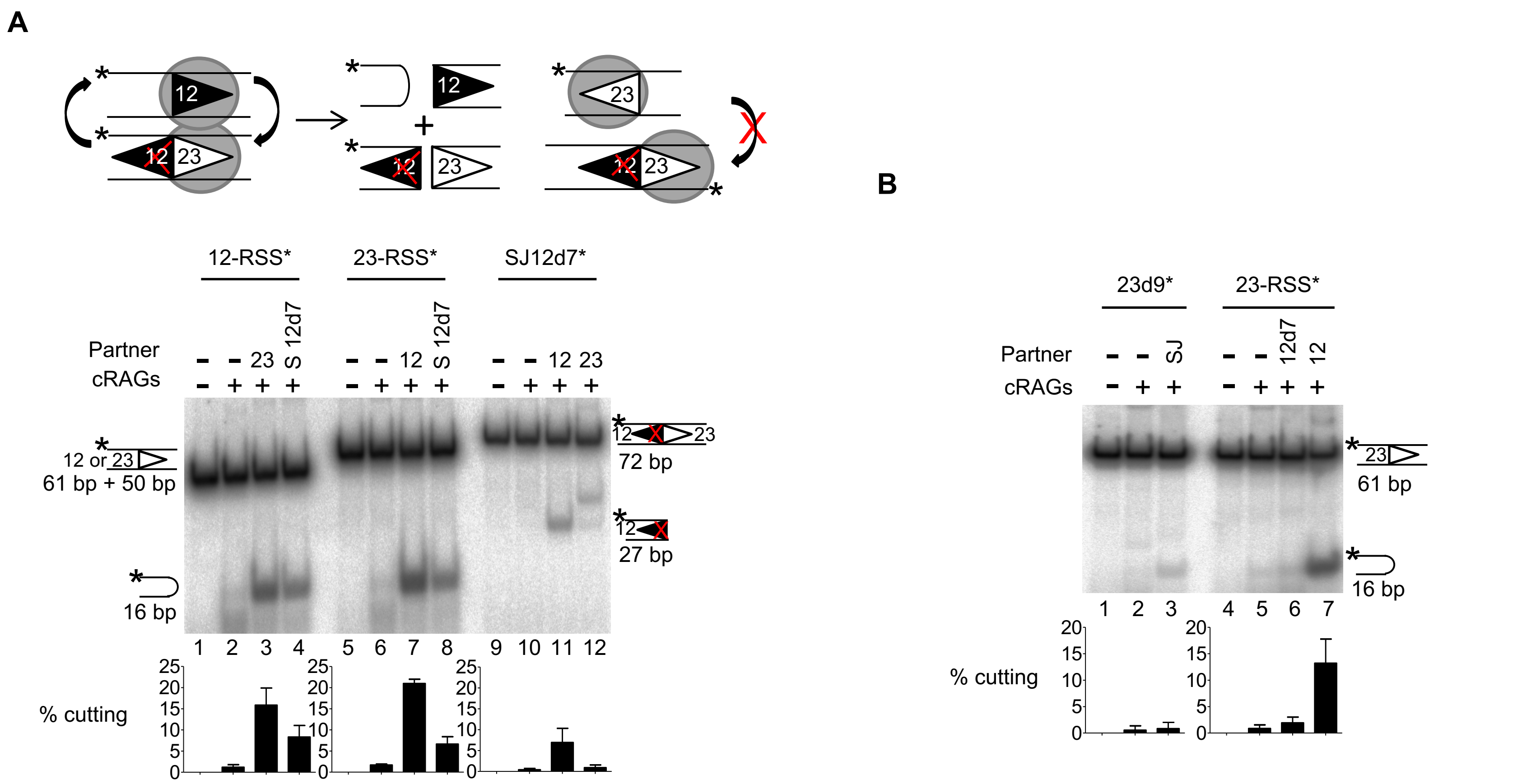

Supplementary Figure 3

Figure S3 – Deletion of the 12-RSS Heptamer within the SJ Restores Symmetric Cleavage, Related to Figure 3. (A) The 12-RSS heptamer within the SJ was deleted and the oligonucleotide used either as a partner for labelled 12- or 23-RSSs or as the substrate. Labelled oligonucleotides are denoted by an asterisk. Symmetrical cutting is restored with a 12-RSS (lanes 4 and 11). Enhanced cutting of SJ12d7 was observed when it was paired with a 23-RSS, to generate a product that is 7 bp bigger than that generated by cutting at the RSS/RSS boundary. This is likely due to the single heptamer being recognised in conjunction with both the 12 bp and 23 bp spacer and cutting occurring at the 3' end of the heptamer when it is paired with a 23-RSS. Graphs represent mean of ≥3 experiments ± SD. **(B)** The SJ mutations significantly reduce RAG cutting. An assumption of the experiments in Figures 3A, 3B and S3A is that mutating either the conserved heptamer or nonamer elements renders that "side" of the SJ unusable. To test this, we used a 12-RSS oligonucleotide where the heptamer sequence is mutated, essentially leaving a nonamer. Since the 12d7 oligonucleotide lacks a heptamer to direct RAG cutting, it was used as a partner with a 23-RSS. As expected, this oligonucleotide was unable to stimulate cutting at a 23-RSS (lane 6). Likewise, no cleavage is detected following mutation of the nonamer within a 23-RSS oligonucleotide (lanes 2-3). Graphs represent mean of four experiments ± SD.

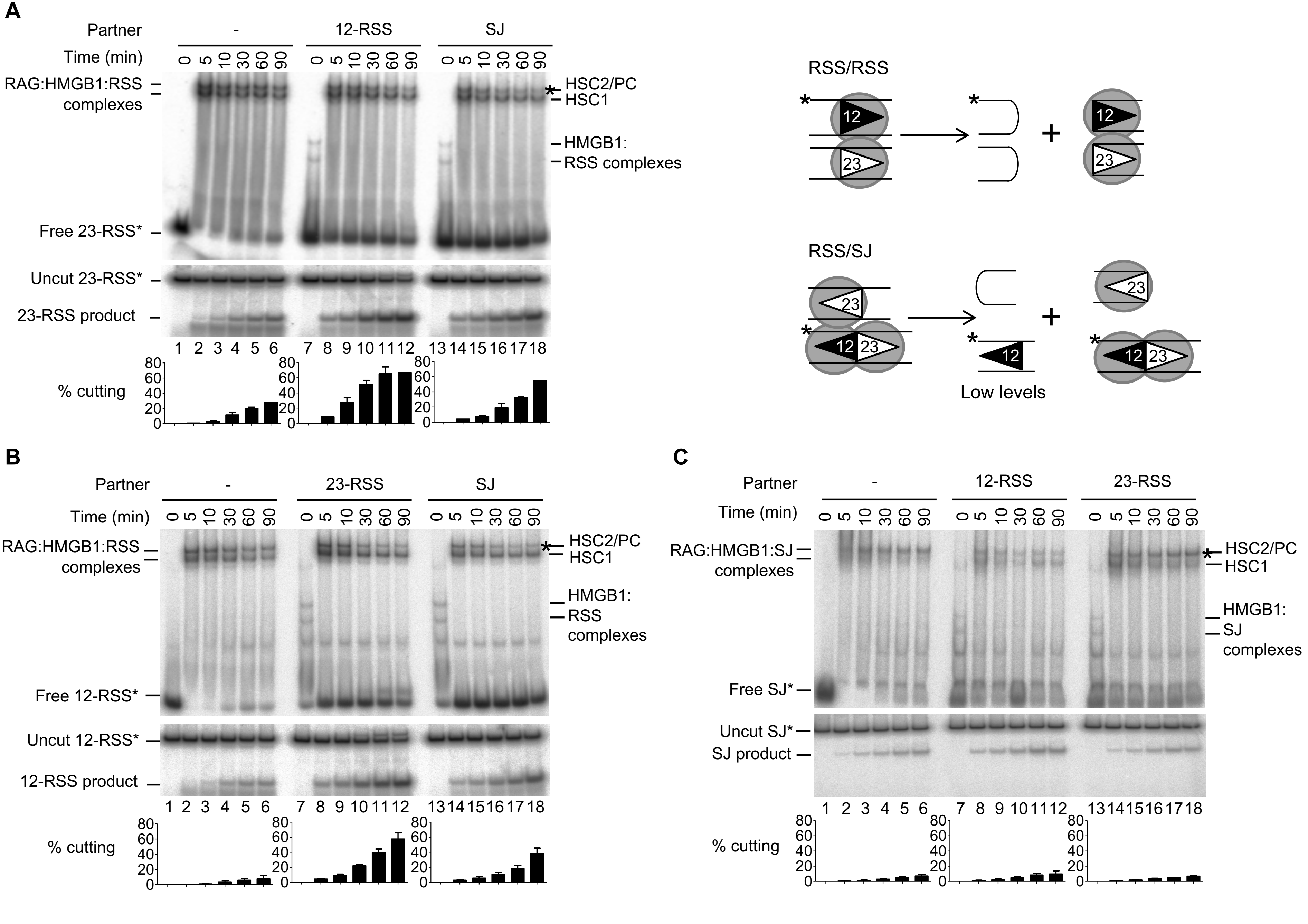

Supplementary Figure 4

Figure S4 – The Coding End of an RSS is Released by RAG Proteins Following Cleavage, Related to Figure 4. RAG cleavage assays were carried out in the presence of Mg^{2+} and the partner indicated with **(A)** a 23-RSS, **(B)** a 12-RSS and **(C)** a SJ. Samples were taken at the times indicated. Half the reaction was run on a 4% polyacrylamide gel to observe complex stability (upper gels), and half was run on a 12% native polyacrylamide gel to observe RAG cutting (lower gels). The HSC2 complex is indicated by an asterisk. In the absence of a partner RSS or SJ, little RAG cutting occurs (lower gels) and the RAG/ labelled RSS complex is stable (lanes 1-6). Upon addition of either a RSS or SJ partner, RAG cutting is enhanced and stability of the HSC2 complex is decreased at an RSS (parts A and B, middle and right panels) but not a SJ (part C; lanes 6-12 and 13-18). With a labelled SJ, complex C shown previously (Figure 2) is not consistently observed. A possible reason for this is that previously, Ca^{2+} was used as the divalent cation to observe precleavage synaptic complexes (Hiom and Gellert, 1997) whereas here, Mg^{2+} was used to facilitate cleavage, which possibly destabilises complex C. Graphs represent mean of three experiments $\pm$ SD.

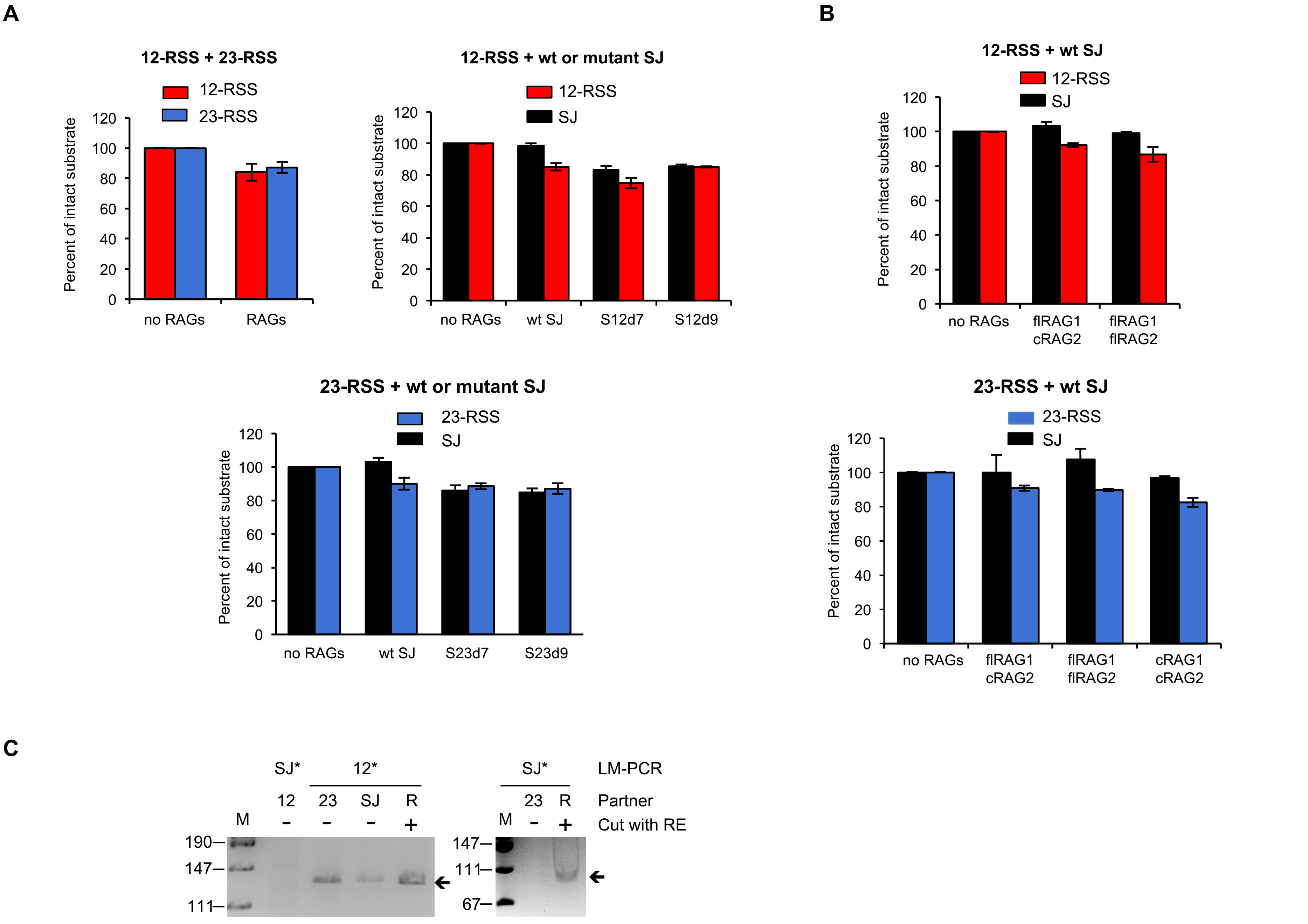

Supplementary Figure 5

Figure S5 – Asymmetric Cutting in SJ-RSS Pairs *in vivo,* Related to Figure 5. A) NIH3T3 cells were transfected with expression vectors for full length RAG1 and full length RAG2, and two substrate plasmids, as indicated. The amount of intact substrate after 48 hours was measured by qPCR. Data were normalised to unique regions within each plasmid, and the values given are relative to no RAG2 controls. Data are represented as mean of three experiments ± SD. **B)** As for (A) except that expression vectors for full-length (fl) RAG1 and RAG2 or core (c) RAG1 and RAG2 were used together with two substrate plasmids, as indicated. Data are represented as mean of three experiments ± SD. **C)** LM-PCR of extra-chromosomal substrate cleavage products. The plasmid samples used in (A) were used in an LM-PCR assay to verify that DSBs are formed. The RSS or SJ element that was amplified by LMPCR is indicated by an asterisk above each gel and the element on the partner plasmid is indicated by “Partner”. A product is detected only with plasmids carrying a 12-RSS and not those carrying a SJ. R indicates positive control samples that were cut with a restriction enzyme (RE; EcoRV for the 12-RSS and ApaLI for the SJ); arrows indicate products of the expected sizes (128 bp for 12-RSS & 92 bp for SJ).

A

Bait DSB site	Experiment	Total translocations	RSS	Not RSS	RSS fraction	Fold increase vs Cas9 only	Fold increase of SJ-mediated breaks at RSSs vs experiment	Significance of SJ-mediated breaks at RSSs vs experiment
ROSA26	Cas9 only	69824	12079	57745	0.172992094	1	1.131700211	p<0.0001
	Cas9 + pWPI (Control virus)	20641	3830	16811	0.185553026	1.072609856	1.055090259	p=0.0004
	Cas9 + 12-RSS	23591	4334	19257	0.183714128	1.061979907	1.065651247	p<0.0001
	Cas9 + 23-RSS	31993	5887	26106	0.184009002	1.063684457	1.063943545	p<0.0001
	Cas9 + SJ12d9	28252	5682	22570	0.201118505	1.162587837	0.973432007	ns
	Cas9 + SJ	106561	20862	85699	0.19577519	1.131700211	1	-
IgH	Cas9 only	9821	1616	8205	0.164545362	1	1.262257139	p<0.0001
	Cas9 + pWPI (Control virus)	6376	1163	5213	0.18240276	1.108525687	1.138681001	p<0.0001
	Cas9 + SJ	9846	2045	7801	0.207698558	1.262257139	1	-

B

	ROSA						IgH		
Experiment	Cas9 only	Cas9 + pWPI (empty virus)	Cas9 + 12-RSS	Cas9 + 23-RSS	Cas9 + SJ12d9	Cas9 + ESC	Cas9 only	Cas9 + pWPI (empty virus)	Cas9 + SJ
**Bait site chromosome**	41737	10789	12687	17680	13542	51321	5862	3294	4006
**Other chromosomes**	28087	9852	10904	14313	14710	55240	3959	3082	5840
**Fraction bait chromosome**	0.59774576	0.52269754	0.53778984	0.552621	0.479329	0.481611	0.59688423	0.51662484	0.40686573
**Significance of SJ-mediated breaks on other chromosomes vs experiment**	p<0.0001	p<0.0001	p<0.0001	p<0.0001	ns	-	p<0.0001	p<0.0001	-

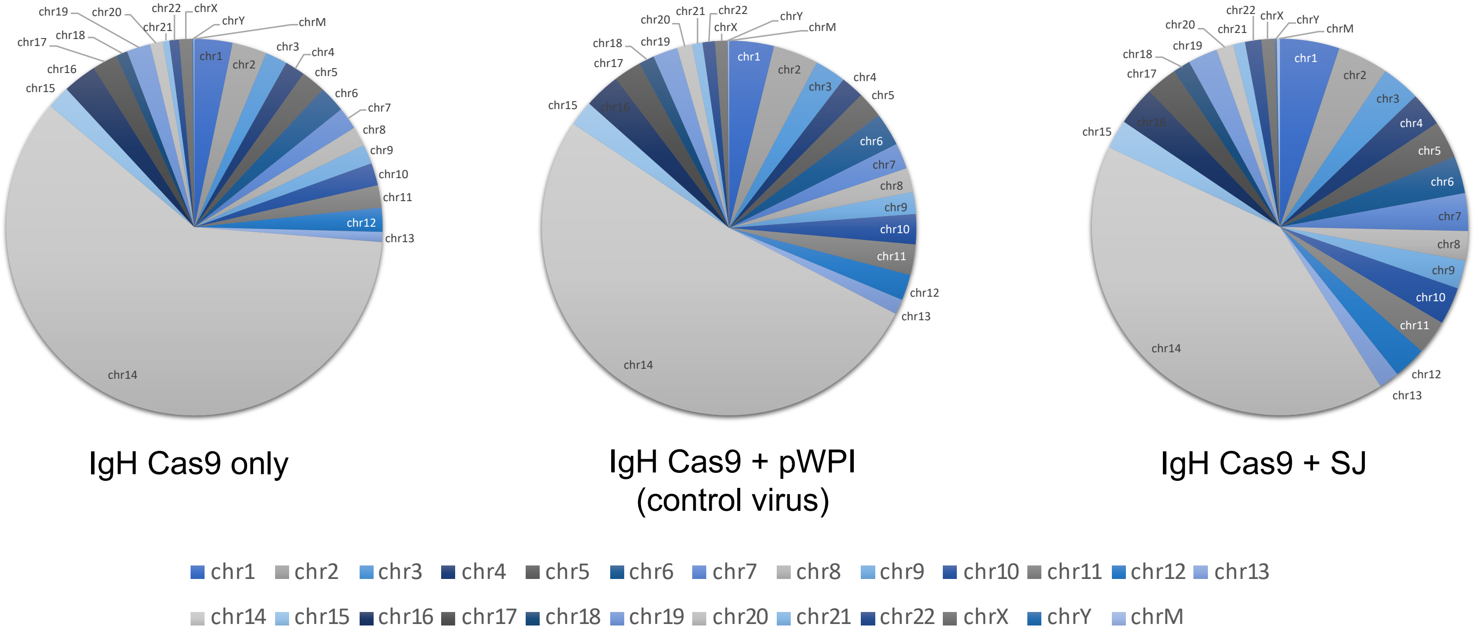

Supplementary Figure 6

Figure S6 – Translocations to RSSs are Increased in the Presence of the SJ, Related to Figure 7. (A) Table showing total number of translocations at the *ROSA26* and *IgH* loci to RSSs, as defined by RSS Site. A sequence is scored as an RSS by RSS Site by its similarity to physiological human RSSs. It is possible that yet more sequences are used as RSSs *in vivo*. Since rare cutting events at relatively poor RSSs are likely to be detected by the translocation assay, the calculation of translocations to RSSs is likely to be conservative. The SJ and SJ12d9 vectors differ by only 7 bp and since there is some residual binding to both RSSs in SJ12d9, it is perhaps not surprising that it also causes breaks at RSSs. The greater effect of SJ12d9 in cutting RSSs compared to the generation of γH2AX foci may be due to the γH2AX assay detecting all cuts whereas the LAM-HTGTS assay detects only the first cut but not subsequent ones due to “run”. If the RAG-SJ12d9 complex is less stable, this may reduce subsequent cuts due to “run”, giving rise to fewer γH2AX foci. **(B)** Table and pie charts of chromosomal distribution of translocations to the *ROSA26* and *IgH* loci in the presence and absence of the SJ. Many of the Cas9 only translocations are to the same chromosome as the break-point, suggesting that they might be the result of processing around the break-point. By contrast, the SJ causes translocations throughout the genome. Consistent with the idea that the SJ causes more breaks than a single RSS, a significant increase in chromosome distribution of breaks with the SJ is observed compared to the 12- and 23-RSS as well as the empty (control) virus (p <0.0001 in each case).

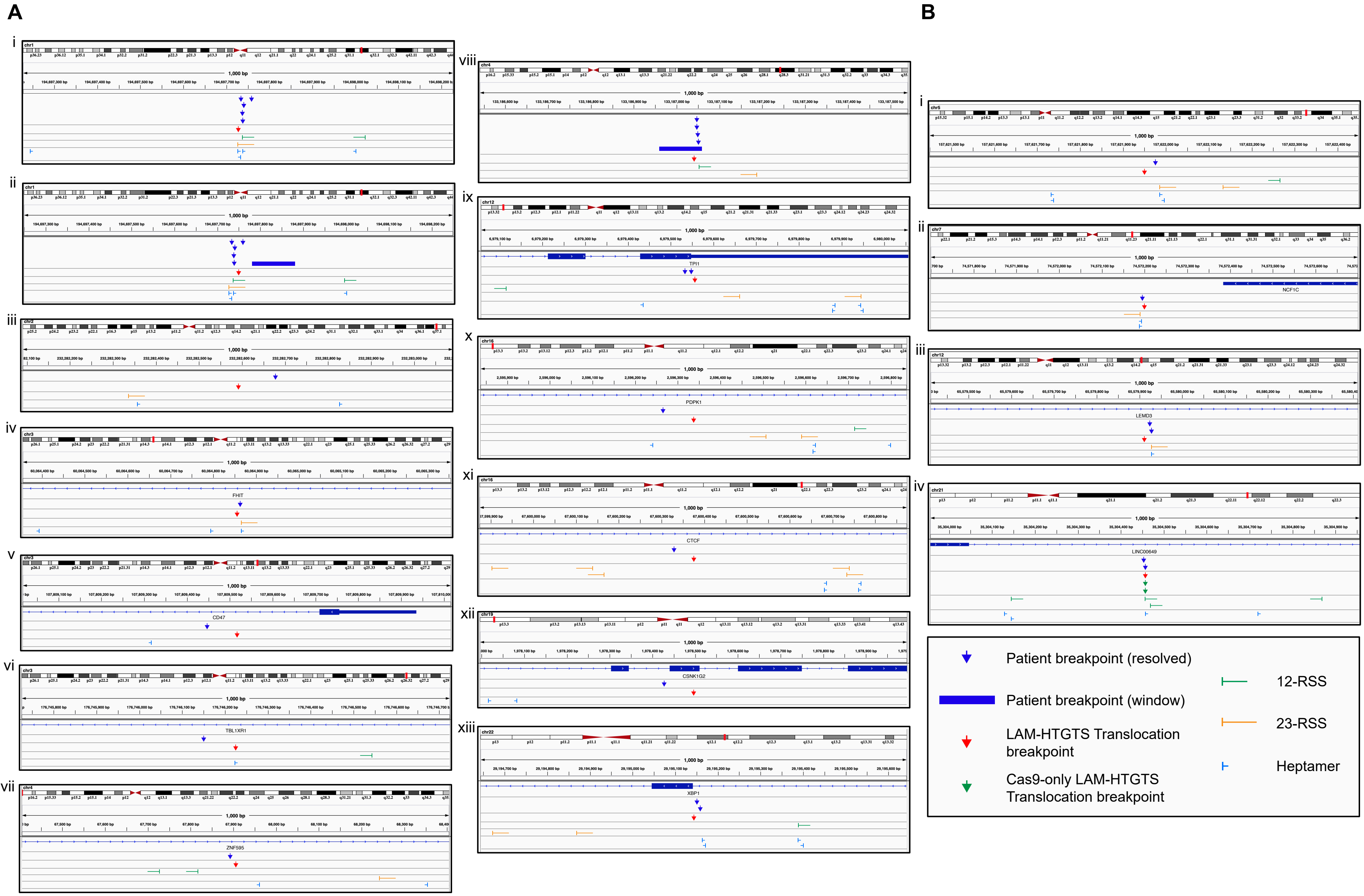

Supplementary Figure 7

Figure S7 – SJ-mediated Breaks Co-localise with those Found in ALL Patients, Related to Figure 7. A) Data showing 13 individual translocations identified by LAM-HTGTS in the presence of the SJ (red arrowheads) that lie within 100 bp of 28 breakpoints in ALL patients with the *ETV6/RUNX1* translocation (blue arrowheads; i-xiii). Some patient breakpoints were not fully resolved and these are represented by blue bars. Panels i and ii show two breaks detected by LAM-HTGTS that occur in close proximity on chromosome 1. These are shown separately since (i) overlaps with five patient breakpoints whereas (ii) overlaps with six. Heptamer and RSS data are taken from the FIMO and RSS Site web-sites, respectively (STAR Methods). **B)** Translocations detected in the presence of SJ12d9 (i-iv). A number of breaks is to be expected since single RSSs cause some genome instability (Han et al., 1999). One of the breakpoints in the presence of SJ12d9 (iv) is detected twice in cells transduced to express Cas9 only, suggesting that this particular break may be due to off-target Cas9 cleavage.

Table S1: Sequence Conservation of most Frequently Generated human ESCs, Related to Figure S2.

Locus	Recombination event	Number of recombination events	ESCs where both heptamers have the consensus sequence and AA is conserved in positions 5 and 6 of both nonamers (%)	ESCs where both heptamers have CAC and at least 6 out of 7 conserved nucleotides and AA is conserved in positions 5 and 6 of both nonamers (%)
Immunoglobulin Heavy Chain (*IgH*)	V to D	1512	49.70	86.24
Immunoglobulin Heavy Chain (*IgH*)	D to J	216	0.00	84.82
Immunoglobulin Kappa (*IgK*)	V to J	200	62.80	90.84
Immunoglobulin Lambda (*IgL*)	V to J	238	18.40	63.6
T Cell Receptor Alpha (*TCRA*)	V to J	2499	21.14	65.16
T Cell Receptor Beta (*TCRB*)	V to D	98	0.00	36.67
T Cell Receptor Beta (*TCRB*)	D to J	26	0.00	19.06
T Cell Receptor Gamma (*TCRG*)	V to J	40	4.22	5.46
T Cell Receptor Delta (*TCRD*)	V to D	6	14.40	20.00
T Cell Receptor Delta (*TCRD*)	D to J	8	0.00	0.00
**Total**		**4843**	**29.98**	**72.12**

Data weighted based on IMGT database of human gene segment usage

Table S2: Overlap between patient breakpoints and translocations mapped via LAM-HTGTS, Related to Figures 7 and S7

Translocation	Translocation chr	Translocation breakpoint	Translocation orientation	Patient breakpoint ID	Breakpoint position	Patient sample ID	RSS within 500 bp	Heptamer within 500 bp
**Cas9**								
1	chr12	123075587	-	37612940	123075442-123075542	PD4013a	Yes	Yes
2	chr20	33412439	-	37733808	33412465	PD4028a	Yes	Yes
3	chr21	35304456	-	37724900	35304456	PD3958a	Yes	Yes
				38057914	35304453	PD4038a		
4	chr21	35304456	-	37724900	35304456	PD3958a	Yes	Yes
				38057914	35304453	PD4038a		
**pWPI**								
1	chr16	69169672	+	37719304	69169579	PD4010a	Yes	Yes
**12-RSS**								
1	chr19	37019097	-	37718050	37019115	PD4030a	Yes	Yes
**23-RSS**								
1	chr8	133765911	+	37446514	133765977	PD3956a	Yes	Yes
2	chr11	36638045	+	37821575	37821575	PD4016a	No	Yes
				37611808	37611808	PD4013a		
				37700046	37700046	PD3958a		
3	chr14	93812808	-	37706408	93812668-93812768	PD3961a	Yes	Yes
4	chrX	38421475	-	37456899	38421395-38421495	PD3968a	Yes	Yes
**SJ12d9**								
1	chr5	157621949	-	37441544	157621975	PD3964a	Yes	Yes
2	chr7	74572198	+	43011562	74572193	PD4020a	Yes	Yes
3	chr12	65579911	-	37519754	65579927	PD4022a	Yes	Yes
				37612853	65579923	PD4013a		
4	chr21	35304456	+	37724900	35304456	PD3958a	Yes	Yes
				38057914	35304453	PD4038a		

**SJ**								
1	chr1	194697727	-	37502102	194697737	PD3971a	Yes	Yes
				37750532	194697739	PD3960a		
				37639400	194697758	PD4018a		
				37526674	194697733	PD3953a		
				37631487	194697736	PD4025a		
2	chr1	194697749	+	37502102	194697737	PD3971a	Yes	Yes
				37750532	194697739	PD3960a		
				37639400	194697758	PD4018a		
				37526674	194697733	PD3953a		
				37631487	194697736	PD4025a		
				38581730	194697780-194697880	PD4035a		
3	chr2	232282590	-	37668781	232282677	PD4018a	Yes	Yes
4	chr3	60064855	-	37981423	60064861	PD4036a	Yes	Yes
5	chr3	107809516	-	37654082	107809446	PD4025a	No	Yes
6	chr3	176746225	-	37657164	176746149	PD4028a	Yes	Yes
7	chr4	67905	+	37516749	67891	PD4022a	Yes	Yes
8	chr4	133187040	-	37603732	133187048	PD4013a	Yes	No*
				37869991	133187050	PD4012a		
				38033843	133187046	PD4037a		
				37663849	133187046	PD4010a		
				37465888	133186959-133187059	PD3965a		
9	chr12	6979556	+	37703949	6979532	PD4010a	Yes	Yes
				37704018	6979547	PD4010a		
10	chr16	2596337	-	37736538	2596265	PD3957a	Yes	Yes
11	chr16	67600374	-	37716657	67600328	PD3973a	Yes	Yes
12	chr19	1978496	-	37718728	1978427	PD3958a	No	Yes
13	chr22	29195143	-	37457724	29195158	PD3959a	Yes	Yes
				37725243	29195150	PD4025a		

*As determined by FIMO motif finder; RSSs were determined by RSS Site

Table S3: Breaks in frequently mutated genes in ALL in the presence of the SJ, Related to Figure 7

	Translocations normalised per 100,000 translocations						SJ enrichment over test:				
	Cas9	pWPI	12-RSS	23-RSS	SJ12d9	SJ	Cas9	pWPI	12-RSS	23-RSS	SJ12d9
IKZF1	0.000	3.704	8.478	6.251	3.540	5.156	TRUE	TRUE	FALSE	FALSE	TRUE
NRAS	1.256	0.000	0.000	0.000	0.000	4.297	TRUE	TRUE	TRUE	TRUE	TRUE
KRAS	0.000	0.000	0.000	0.000	0.000	0.859	TRUE	TRUE	TRUE	TRUE	TRUE
PAX5	10.048	0.000	4.239	12.503	3.540	11.172	TRUE	TRUE	TRUE	FALSE	TRUE
CDKN2A	0.000	0.000	0.000	0.000	0.000	0.859	TRUE	TRUE	TRUE	TRUE	TRUE
FLT3	2.512	0.000	0.000	3.126	0.000	4.297	TRUE	TRUE	TRUE	TRUE	TRUE
JAK2	1.256	0.000	0.000	3.126	0.000	1.719	TRUE	TRUE	TRUE	FALSE	TRUE
TP53	1.256	0.000	4.239	0.000	3.540	1.719	TRUE	TRUE	FALSE	TRUE	FALSE
PTPN11	5.024	3.704	0.000	3.126	0.000	4.297	FALSE	TRUE	TRUE	TRUE	TRUE
CREBBP	11.304	29.629	21.195	6.251	10.619	18.047	TRUE	FALSE	FALSE	TRUE	TRUE
JAK1	2.512	0.000	4.239	3.126	3.540	5.156	TRUE	TRUE	TRUE	TRUE	TRUE
Number of genes enriched with SJ vs test:							10	10	8	8	10
Significance of SJ vs test:							0.00022	0.00029	0.021	0.018	0.00019

(B) Frequently mutated genes in *ETV6/RUNX1*-positive ALL

	Translocations normalised per 100,000 translocations						SJ enrichment over:				
	Cas9	pWPI	12-RSS	23-RSS	SJ12d9	SJ	Cas9	pWPI	12-RSS	23-RSS	SJ12d9
ETV6	0.000	3.704	8.478	0.000	0.000	3.438	TRUE	FALSE	FALSE	TRUE	TRUE
TBL1XR1	1.256	0.000	4.239	3.126	3.540	8.594	TRUE	TRUE	TRUE	TRUE	TRUE
PAX5	10.048	0.000	4.239	12.503	3.540	11.172	TRUE	TRUE	TRUE	FALSE	TRUE
ATF7IP	2.512	3.704	4.239	9.377	3.540	6.875	TRUE	TRUE	TRUE	FALSE	TRUE
BTG1	1.256	0.000	0.000	0.000	0.000	1.719	TRUE	TRUE	TRUE	TRUE	TRUE
RAG2	0.000	0.000	0.000	3.126	0.000	0.859	TRUE	TRUE	TRUE	FALSE	TRUE
BTLA	0.000	0.000	0.000	0.000	0.000	0.000	-	-	-	-	-
NR3C2	3.768	3.704	8.478	0.000	3.540	3.438	FALSE	FALSE	FALSE	TRUE	FALSE
CDKN2A	0.000	0.000	0.000	0.000	0.000	0.859	TRUE	TRUE	TRUE	TRUE	TRUE
KRAS	0.000	0.000	0.000	0.000	0.000	0.859	TRUE	TRUE	TRUE	TRUE	TRUE
STAG2	0.000	3.704	4.239	0.000	7.079	2.578	TRUE	FALSE	FALSE	TRUE	FALSE
ZMYM2	5.024	11.111	0.000	0.000	7.079	3.438	FALSE	FALSE	TRUE	TRUE	FALSE
MGA	3.768	7.407	0.000	6.251	3.540	8.594	TRUE	TRUE	TRUE	TRUE	TRUE
Number of genes enriched with SJ vs test:							10	8	9	9	9
Significance of SJ vs test:							0.0027	0.056	0.023	0.019	0.012

Table S4: Oligonucleotides used in RAG cutting assays, Related to Star Methods

Oligonucleotide	Sequence
12-RSS top (DAR39)	5’-GATCTGGCCTGTCTTACACAGTGCTACAGACTGGAACAAAAACCCTGCAG-3’
12-RSS bottom (DAR40)	5’-CTGCAGGGTTTTTGTTCCAGTCTGTAGCACTGTGTAAGACAGGCCAGATC-3’
23-RSS top (DG61)	5’-GATCTGGCCTGTCTTACACAGTGGTAGTACTCCACTGTCTGGCTGTACAAAAACCCTGCAG-3’
23-RSS bottom (DG62)	5’-CTGCAGGGTTTTTGTACAGCCAGACAGTGGAGTACTACCACTGTGTAAGACAGGCCAGATC-3’
SJ1 top (MBNSJ top)	5’-CTGCAGGGTTTTTGTTCCAGTCTGTAGCACTGTGCACAGTGGTAGTACTCCACTGTCTGGCTGTACAAAAACCCTGCAG-3’
SJ1 bottom (MBNSJ bottom)	5’-CTGCAGGGTTTTTGTACAGCCAGACAGTGGAGTACTACCACTGTGCACAGTGCTACAGACTGGAACAAAAACCCTGCAG-3’
SJ2 top	5’-CTGCAGGGTTTTTGTTCCAGTCTGTAGCA**T**TGTGCACAGTGGTAGTACTCCACTGTCTGGCTGTACAAAAACCCTGCAG-3’
SJ2 bottom	5’-CTGCAGGGTTTTTGTACAGCCAGACAGTGGAGTACTACCACTGTGCACA**A**TGCTACAGACTGGAACAAAAACCCTGCAG-3’
SJ3 top	5’-CTGCAGGGTTTTTGTTCCAGTCTGTAG**GG**CTGTGCACAGTGGTAGTACTCCACTGTCTGGCTGTACAAAAACCCTGCAG-3’
SJ3 bottom	5’-CTGCAGGGTTTTTGTACAGCCAGACAGTGGAGTACTACCACTGTGCACAG**CC**CTACAGACTGGAACAAAAACCCTGCAG-3’
Non-specific top (DAR81)	5’-GATCTCGCCTCTCTTAGGTTAATCCTATAGAACTCGTCCCCGTACCTCGAG-3’

Non-specific bottom (DAR82)	5’-CTAGAGCGGAGAGAATCCAATTAGGATATCTTGAGCAGGGGCATGGAGCTC-3’
SJ12d7 top	5’-CTGCAGGGTTTTTGTTCCAGTCTGTAGCACAGTGGTAGTACTCCACTGTCTGGCTGTACAAAAACCCTGCAG-3’
SJ12d7 bottom	5’-GACGTCCCAAAAACAAGGTCAGACATCGTGTCACCATCATGAGGTGACAGACCGACATGTTTTTGGGACGTC-3’
SJ23d9 top	5’-CTGCAGGGTTTTTGTTCCAGTCTGTCGCACTGTGCACAGTGGTAGTACTCCACTGTCTGGCTGTCTGCAG-3’
SJ23d9 bottom	5’-CTGCAGACAGCCAGACAGTGGAGTACTACCACTGTGCACAGTGCGACAGACTGGAACAAAAACCCTGCAG-3’
SJ12d9 top	5’-CTGCAGTCCAGTCTGTAGCACTGTGCACAGTGGTAGTACTCCACTGTCTGGCTGTACAAAAACCCTGCAG-3’
SJ12d9 bottom	5’-CTGCAGGGTTTTTGTACAGCCAGACAGTGGAGTACTACCACTGTGCACAGTGCTACAGACTGGACTGCAG-3’
12d7 top	5’-GATCTGGCCTGTCTTACTACAGACTGGAACAAAAACCCTGCAG-3’
12d7 bottom	5’-CTGCAGGGTTTTTGTTCCAGTCTGTAGCACTGTGTAAGACAGGCCAGATC-3’
23d9 top	5’-GATCTGGCCTGTCTTACACAGTGGTAGTACTCCACTGTCTGGCTGTGCGCTTATTCTGCAG-3’
23d9 bottom	5’-CTGCAGAATAAGCGCACAGCCAGACAGTGGAGTACTACCACTGTGTAAGACAGGCCAGATC-3’
LMO2 top	5’-GATCTGGCCTGTCTTACACAGT**A**GTAGTAGGCTGT**G**CAA**T**AA**TT**CTGCAG-3’
LMO2 bottom	5’-CTGCAG**AA**TT**A**TTG**C**ACAGCCTACTAC**T**ACTGTGTAAGACAGGCCAGATC-3’

Table S5: Oligonucleotides used in PCR assays, Related to Star Methods

Oligonucleotide	Sequence	Tm °C
JH290F	5'-GCACGACAGGTTTCCCGACTGG-3'	61
JH290R	5'-CCAGCTGAACGGTCTGGTTATAGGTAC-3'	61
23-RSS Cut Fwd	5'-CTGGCTAGCGTTTAAACTTAAGC-3'	60
23-RSS Cut Rev	5'-CTATGATCGATTCACTGACTGTAGA-3'	60
12-RSS Cut Fwd	5'-CCTGTGCTGGGAGACCTG-3'	60
12-RSS Cut Rev	5'-AGACGCCCGAATCTCACC-3'	60
Neo3 Fwd	5'-TGCTCCTGCCGAGAAAGTATC-3'	60
Neo3 Rev	5'-TTTCGCTTGGTGGTCGAATG-3'	60
SJ Cut Fwd	5'-CTGGCTAGCGTTTAAACTTAAGC-3'	60
SJ Cut Rev	5'-TGTTTTTCCAAAGCGGTCGAG-3'	60
Jdnorm Fwd	5'-GTCGCCTTCTTGCACATTACTC-3'	60
Jdnorm Rev	5'-AACAACAGATGGCTGGCAAC-3'	60
12-RSS virus Norm Fwd	5'-CCACCTTCCAGCAAGCTTG-3'	60
Virus Norm Rev	5'-CCCGTAGTTTTAGAAGGCACAG-3'	60
23-RSS virus Norm Fwd	5'-GGTACAGTGCAGGGGAAAGAATAG-3'	60
23-RSS virus Norm Rev	5'-GTGAATCGATCATAGTCTAGAGGG-3'	60
SJ virus Norm Fwd	5'-CGTCGCCTTCTTGCACATTAC-3'	60
FM25	5'-GCGGTGACTCGGGAGATCTGAAGTG-3'	-
FM11	5'-CACTTCAGATC-3'	-
CMV fwd	5'-CGCAAATGGGCGGTAGGCGTG-3'	57
12-RSS fwd	5'-CTTCCTGTGCTGGGAGACCT-3'	60
BGHRev	5'-TAGAAGGCACAGTCGAGG-3'	55
V1SacIF	5'-GCCAACTGGGTCCAAGAAAAAC-3'	58
J1StyIR	5'-GCACCTCAAGTCTTGGAGAG-3'	58
V1RSSF	5'-GGCACAGACTGAGGATGAG-3'	55
J1RSSR	5'-CAGTCAGTTTGGTTCCTCCA-3'	55
Vend2nd	5'-CCTGTTAAGAAGATGGTAGTTATGAGACTG	61

	TACC-3'	
Jend3	5'-CCTATGAGGACATATGGATCCTGGGAAGA AGG-3'	63
Jk5 end first	5'-CCTGTGCATCAATAGAAGATCC-3'	54
Jk5 end second	5'-CTTAGGCTTCTGAGACCAGT-3'	54
Jk5 cdjt first	5'-GAACTGACTTTAACTCCTAACATG-3'	53
Jk5 cdnd first	5'-GTACTTACGTTTCAGCTCCAGC-3'	56
Vk3 end first	5'-GTAATTTTTACGAATAAACCTATAGC-3'	50
Vk3 end second	5'-GAGCCTCAGTGAGCTGCAAC-3'	58
Vk3 cdnd first	5'-GAATATTATGGCACAAGTTTAATGCAG-3'	54
Vk3 cdnd second	5'-CCTCATCTATGCTGCATCCAACG-3'	58
Vk16 end first	5'-CATTCTCCATGGGTTCATGCT-3'	54
Vk16 end second	5'-GCCCAACATCTGGTTTCTGC-3'	56
Vk16 cdjt first	5'-GCCTGGTATCAAGAGAAACC-3'	54
Vk16 cdnd first	5'-GGATCCACTTTGCAATCTGG-3'	54
GAPDH F	5'-ACTTTCTTGTGCAGTGCCAGC-3'	56
GAPDH R2	5'-GCACACTTCGCACCAGCATC-3'	56
Vend1	5'-CAGACTAGGGAGATGTAGCCACCTG-3'	61
Jend1	5'-CCTATGAGGACATATGGATCCTGGGAAGA AGG-3'	63
Vendnested	5'-CTCGGGAGATCTGAAGTGCACAATG-3'	60
VendApaLI	5'-CGGGAGATCTGAAGTGTGCACAATG-3'	60
Jendnested	5'-CTCGGGAGATCTGAAGTGCACTGTG-3'	61
JendApaLI	5'-CTCGGGAGATCTGAAGTGTGCACTGTGATA T-3'	61
Genomic DNA PCR 680bp Fwd	5'-TCATTCCCAGTGTTGATGCC-3'	54
Genomic DNA PCR 680bp Rev	5'-TGGGTCCACTCTGAAGGAAC-3'	56
Genomic DNA qPCR 200bp Fwd	5'-ACCATGTCCTTCCCGATCTG-3'	56
Genomic DNA qPCR 200bp Rev	5'-TGGGTCCACTCTGAAGGAAC-3'	56

hROSA26-Bio primer	5'-/5Biosg/ TTCCTGGGTCAGGACGCTTC-3'	
hROSA26-I5-Nested	5'-ACACTCTTTCCCTACACGACGCTCTTCCGA TCT**BARCODE**GGAACTGCCTACTCCACGTT-3'	
IgH-Bio primer	5'-/5Biosg/ GGAAGGTGGAGGCTCTGAGC-3'	
IgH-I5_Nested-control	5'-ACACTCTTTCCCTACACGACGCTCTTCCGA TCT**BARCODE**ATGCTGCCACTTCTAGAGCA-3'	
12-RSS 1st round	5'-GATCACGAGACTAGCCTCG-3'	56
12-RSS 2nd round	5'-CTGTGCTGGGAGACCTGGGC-3'	62
23-RSS/SJ23d9-12-RSS 1st round	5'-GGAATTCCTGCAGCCCGTAG-3'	58
23-RSS 2nd round	5'CCTGCAGCCCGTAGTTTTAATACGACTCACT ATAGGG-3'	65
SJ-12-RSS/SJ12d7-23-RSS 1st round	5'-GATCACGAGACTAGCCTCGAG-3'	58
SJ-12-RSS 2nd round	5'GCTAGCGTTTAAACTTAAGCTTGGTACCGA GCTCGG-3'	65
SJ-23-RSS 1st round	5'-AGTCGAGGCTGATCAGCGGG-3'	60
SJ-23-RSS 2nd round	5'-TGTTTTTCCAAAGCGGTCGAGCTGCAGGG - 3'	64
1st round reverse (DR20)	5'-GCTATGTACTACCCGGGAATTCGTG-3'	59
Ligation primer (DR19)	5'-CACGAATTCCC -3'	
2nd round reverse	5'-GTACTACCCGGGAATTCGTGCACAG-3'	61

Table S6: Plasmids, Related to Star Methods

Plasmid	Description
**pEF-McR1**	The cDNA for core RAG1 (amino acids 384-1008) was cloned in frame with a maltose binding protein tag into the Xba I and Bam HI sites of pEF-XC (Mizushima and Nagata, 1990).
**pEF-McR2**	The cDNA for core RAG2 (amino acids 1-387) was cloned in frame with a maltose binding protein tag into the Xba I and Bam HI sites of pEF-XC (Mizushima and Nagata, 1990).
**pJH290**	A 12- and 23-RSS are cloned in a convergent orientation 329 bp apart (Steen et al., 1996; Han et al., 1999)
**pJH29012SJ + pJH29023SJ**	As above but where an SJ replaces one of the RSSs; pJH29012SJ thus has a 12-RSS and an SJ and pJH29023SJ has a 23-RSS and an SJ
**pSJ+**	Oligonucleotides containing a consensus SJ oligonucleotide with terminal Bam HI and Xho I sites were annealed and cloned into the polylinker of pcDNA3.1. An additional 120 bp Sal I/Bam HI fragment was cloned into the Xho I site of the polylinker to enable the SJ plasmid to be distinguished from the 12- and 23-RSS plasmids.
**p23+**	Oligonucleotides containing a consensus 23-RSS oligonucleotide with terminal Bam HI and Hind III sites were annealed and ligated into pcDNA3.1 cut with Bam HI and Hind III. To remove a cRSS in the polylinker of pcDNA3.1, the vector was digested with Eco RI and Xba I and the oligonucleotide sequence AATTCTCTACAGTCAGTGAATCGATCATAGT was ligated in to give a unique sequence that could be used for as a primer for PCR.
**p12+**	Oligonucleotides containing a consensus 12-RSS oligonucleotide with terminal Bam HI and Hind III sites ligated into the polylinker of pcDNA3.1. To remove a cRSS in the polylinker of pcDNA3.1, the vector was digested with Eco RI and Xba I and the oligonucleotide sequence

	AATTCGTCTCACATCGATGAACTATTGTATGT was ligated in to give a unique sequence that could be used for as a primer for PCR.
**pSJ12d7+ and pSJ23d7+**	The SJ sequence in pSJ was replaced with a mutant SJ sequence in which the heptamer in the 12-RSS or 23-RSS within the SJ was deleted (SJ12d7 and SJ23d7, respectively).
**pSJ12d9+ and pSJ23d9+**	The SJ sequence in pSJ was replaced with a mutant SJ sequence in which the nonamer in the 12-RSS or 23-RSS within the SJ was mutated (SJ12d9 and SJ23d9, respectively).
**pSJ12d9CAC+ and pSJ23d9CAC+**	As above but the CAC of the heptamer was additionally mutated to TAT
**pSJ12d923d9CAC+**	The RSSs in pSJ+ were mutated in both 12- and 23-nonamers and heptamers. The heptamer mutations changed CAC to TAT in each RSS. The nonamer mutations were as above.
**pJH548 (RAG1 expression vector)**	The full length mouse RAG1 cDNA was cloned into pCDM8 so that the cDNA was expressed via the CMV immediate early promoter (Sadofsky et al 1993).
**pEFflRAG2 (full length RAG2 expression vector)**	The full length mouse RAG2 cDNA was cloned into the pEF-XC vector (Mizushima and Nagata, 1990) so that the cDNA was expressed via the strong EF1α promoter.
**pEFcRAG2 (core RAG2 expression vector)**	Core mouse RAG2 cDNA (amino acids 1-387) was cloned into the pEF-XC vector (Mizushima and Nagata, 1990) so that the cDNA was expressed via the strong EF1α promoter.
**pWPI**	Second generation bicistonic lentiviral vector to allow for simultaneous expression of a transgene and EGFP marker. A gift from Didier Trono (Addgene plasmid #12254).
**pWPI-12RSS**	The 12-RSS sequence was amplified from p12+ using the T7 forward and bGH reverse primers. The PCR product was cloned into the Pme I site of pWPI.
**pWPI-23RSS**	As above but the 23-RSS was amplified from p23+.

**pWPI-SJ and pWPI-mutant SJ vectors**	As above but the SJ sequence was amplified from pSJ+ whilst the mutant SJ sequences were amplified from the respective mutant pSJ+ plasmids.
