# Supplementary material for: Cut-and-Run: A Distinct Mechanism by which V(D)J Recombination Causes Genome Instability
Source: Mol Cell. 2019 May 2;74(3):584–597.e9. doi: 10.1016/j.molcel.2019.02.025 (PMC6509286; doi:10.1016/j.molcel.2019.02.025)
Supplement: Document S2. Article plus Supplemental Information [file mmc2.pdf]

Molecular Cell

Article

Cut-and-Run: A Distinct Mechanism by which V(D)J Recombination Causes Genome Instability

Graphical Abstract

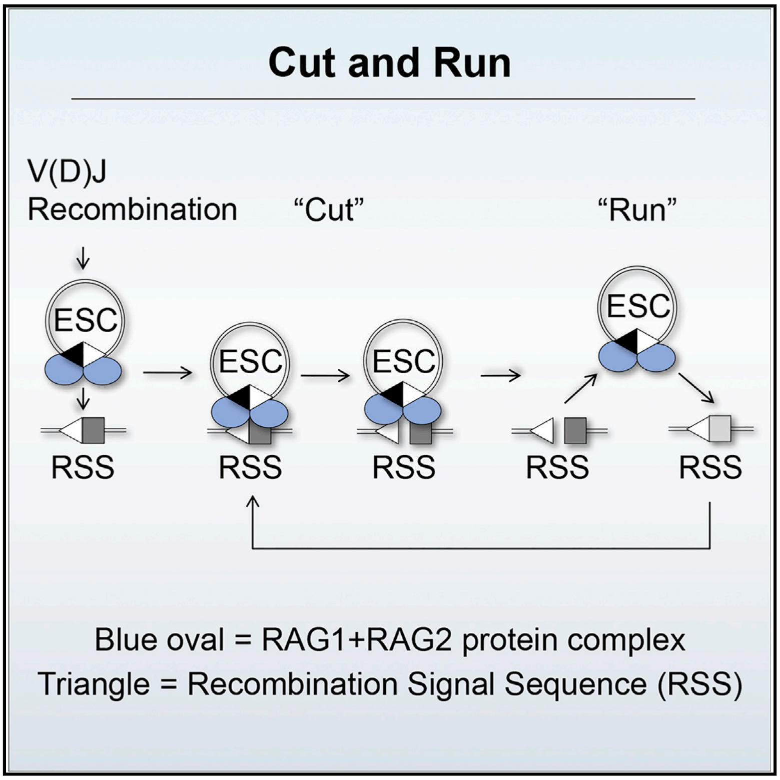

Highlights

- A complex between the recombination by-product and RAGs triggers multiple DNA breaks
- The breaks co-localize with chromosome breakpoints in acute lymphoblastic leukemias
- The breaks occur at many frequently mutated genes in acute lymphoblastic leukemia
- Cut-and-run may underpin the most common types of lymphocyte chromosome instabilities

Authors

Christopher M. Kirkham, James N.F. Scott, Xiaoling Wang, ..., Peter G. Stockley, Roman Tuma, Joan Boyes

Correspondence

j.m.boyes@leeds.ac.uk

In Brief

V(D)J recombination errors have long been associated with chromosome alterations in lymphoid cancers. Here, Kirkham et al. describe an unexpected reaction where the recombination by-product complexes with the RAG recombinase to trigger DNA breaks throughout the genome. Notably, these breaks co-localize with those found in acute lymphoblastic leukemia.

Kirkham et al., 2019, Molecular Cell *74*, 584–597
May 2, 2019 © 2019 The Author(s). Published by Elsevier Inc.
https://doi.org/10.1016/j.molcel.2019.02.025

CellPress

Molecular Cell
Article

Cut-and-Run: A Distinct Mechanism by which V(D)J Recombination Causes Genome Instability

Christopher M. Kirkham,[1,3,7] James N.F. Scott,[1,7] Xiaoling Wang,[1,7] Alastair L. Smith,[1,4] Adam P. Kupinski,[1,5] Anthony M. Ford,[2] David R. Westhead,[1] Peter G. Stockley,[1] Roman Tuma,[1,6] and Joan Boyes[1,8,*]
[1]School of Molecular and Cellular Biology, Faculty of Biological Sciences, University of Leeds, Leeds LS2 9JT, UK
[2]Centre for Evolution and Cancer, The Institute of Cancer Research, London SM2 5NG, UK
[3]Present address: Charles River Laboratories, Inc., Bristol BS20 7AW, UK
[4]Present address: MRC Molecular Haematology Unit, Weatherall Institute of Molecular Medicine, University of Oxford, John Radcliffe Hospital, Headington, Oxford OX3 9DS, UK
[5]Present address: Ipsen Bioinnovation, Milton Park, Abingdon OX14 4RY, UK
[6]Present address: Faculty of Science, University of South Bohemia, Ceske Budejovice, Czech Republic
[7]These authors contributed equally
[8]Lead Contact
*Correspondence: j.m.boyes@leeds.ac.uk
https://doi.org/10.1016/j.molcel.2019.02.025

SUMMARY

V(D)J recombination is essential to generate antigen receptor diversity but is also a potent cause of genome instability. Many chromosome alterations that result from aberrant V(D)J recombination involve breaks at single recombination signal sequences (RSSs). A long-standing question, however, is how such breaks occur. Here, we show that the genomic DNA that is excised during recombination, the excised signal circle (ESC), forms a complex with the recombinase proteins to efficiently catalyze breaks at single RSSs both *in vitro* and *in vivo*. Following cutting, the RSS is released while the ESC-recombinase complex remains intact to potentially trigger breaks at further RSSs. Consistent with this, chromosome breaks at RSSs increase markedly in the presence of the ESC. Notably, these breaks colocalize with those found in acute lymphoblastic leukemia patients and occur at key cancer driver genes. We have named this reaction "cut-and-run" and suggest that it could be a significant cause of lymphocyte genome instability.

INTRODUCTION

V(D)J recombination stochastically joins individual V, D, and J gene segments, to generate vast arrays of immunoglobulin and T cell receptor genes that enable vertebrates to combat almost infinite numbers of potential pathogens. Despite its obvious advantages, V(D)J recombination is an inherently dangerous process because it involves breaking and rejoining DNA. Indeed, recombination errors have long been associated with chromosome alterations (Küppers, 2005; Roth, 2003). One of the most common recombination errors is end donation (Roth, 2003), which occurs when a broken recombination signal sequence (RSS) is joined with a broken DNA end that was formed independently, for example, via ionizing radiation. If this brings an oncogene under the control of the strong transcriptional elements of the antigen receptor loci, malignant transformation can result. This process is thought to underpin 30%–40% of the chromosome translocations found in follicular and mantle cell lymphomas (Jäger et al., 2000; Welzel et al., 2001).

More recently, whole genome sequencing studies of acute lymphoblastic leukemia (ALL) patients carrying the *ETV6/RUNX1* translocation have further implicated broken RSSs in the generation of chromosome alterations (Papaemmanuil et al., 2014). Both interstitial deletions and chromosome rearrangements at RSSs were observed and these are thought to play an integral role in disease progression from pre- to overt leukemia. Notably, ~50% of the RAG-mediated chromosome alterations appear to involve only single RSSs (Papaemmanuil et al., 2014). However, a fundamental question is how V(D)J recombination generates single broken RSSs for this and the end donation reaction, as stringent measures exist throughout the reaction to prevent the release of single broken ends.

Indeed, initiation of V(D)J recombination is restricted so that it occurs primarily within a synaptic complex between the two regions that ultimately become joined. Here, the lymphocyte-specific proteins, RAG1 and RAG2, bind to the RSSs, which lie adjacent to V, D, and J gene segments and consist of conserved heptamer and nonamer sequences, separated by 12 ± 1 bp or 23 ± 1 bp non-conserved spacers (Gellert, 2002; Schatz and Swanson, 2011). RAG1 binds to the RSS nonamer and following capture of a partner RSS to form a synaptic complex, RAG2 directs the RAG1 DDE catalytic site (Fugmann et al., 2000; Kim et al., 1999; Landree et al., 1999) to cleave the partner RSS precisely at the boundary between the heptamer and coding sequence (Kim et al., 2018; Ru et al., 2015; Schatz and Swanson, 2011; Yin et al., 2009). This ensures that cleavage is restricted to a pair of RSSs only after they are brought into very close proximity.

 © 2019 The Author(s). Published by Elsevier Inc.
This is an open access article under the CC BY license (http://creativecommons.org/licenses/by/4.0/).

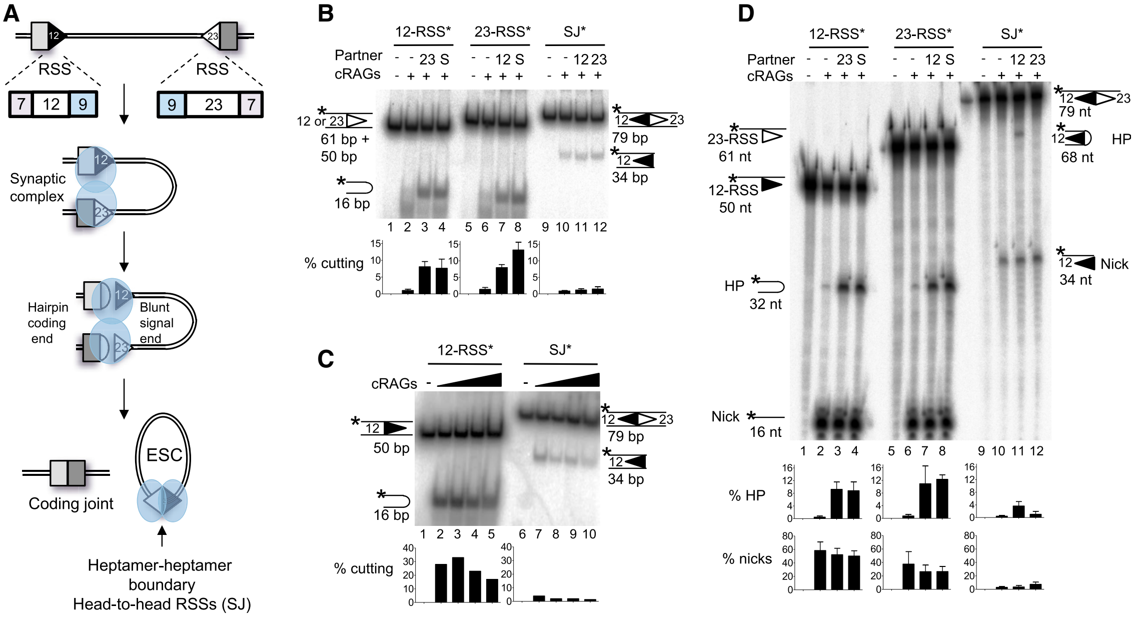

Figure 1. SJ-RSS Cleavage Is Asymmetric

(A) Cartoon of deletional V(D)J recombination and the generation of an ESC.

(B) A SJ stimulates cleavage of 12- and 23-RSSs, but not vice versa. RAG cutting assays were performed using radiolabeled oligonucleotides, denoted by an asterisk above each set of lanes, carrying a 12-RSS, a 23-RSS, or a SJ sequence and separated on a native polyacrylamide gel. Unlabeled partner RSSs are present as indicated; S = SJ. Graphs represent mean of five experiments ± SD.

(C) SJ-RSS cleavage is asymmetric at a range of core RAG concentrations. As for (B) except the amount of core RAG proteins was increased over an 8-fold range, indicated by the filled arrow; a 23-RSS partner was present in all reactions.

(D) Cleavage reactions were performed as in (B) and uncut, nicked, and hairpinned (HP) DNA was separated on a denaturing gel. nt, nucleotides. Graphs represent mean of three experiments ± SD.

See also Figures S1 and S2 and Table S1.

Extensive measures then promote correct joining of the broken DNA ends. Initially, RAGs retain the four broken ends prior to their transfer to the classical non-homologous end joining (cNHEJ) machinery (Lee et al., 2004). Although the transfer mechanism is poorly understood, the acidic hinge region in the RAG2 C terminus plays a central role in shepherding the ends along the cNHEJ pathway, rather than the error-prone alternative NHEJ (aNHEJ) pathway (Coussens et al., 2013; Gigi et al., 2014). Ku70/Ku80 then bind to the signal ends, while the DNA-dependent protein kinase catalytic subunit (DNA-PKcs) and Artemis associate with the coding ends. The latter are tethered during processing by complexes involving DNAPK, MRN, ATM, and the XRCC4-associated protein XLF, prior to end joining by DNA ligase IV (reviewed by Helmink and Sleckman, 2012). Signal ends remain bound to RAG proteins for longer (Livak and Schatz, 1996; Ramsden and Gellert, 1995) before becoming directly ligated to generate an excised signal circle (ESC) (Figure 1A).

Beyond this extensive network of end joining proteins, yet further safeguards prevent the release of broken DNA ends: in cells deficient in cNHEJ proteins, RAG cutting results in cells being directed to apoptosis via the p53-mediated pathway (reviewed by Helmink and Sleckman, 2012). Absence of functional ATM, however, does result in increased chromosome deletions and translocations, particularly involving the *TCRα/δ* locus (Liyanage et al., 2000). Translocations similarly result when loss of XLF or p53 is combined with a non-functional RAG2 C terminus (Lescale et al., 2016). Thus, except in ATM-negative cells or engineered double mutant cells, the release of single broken RSS ends is likely to be rare. Here, we describe an unexpected mechanism that generates broken RSSs that are potential substrates for the observed chromosome alterations.

This mechanism involves the recombination by-product, the ESC. This retains the signal joint (SJ) where the RSSs are joined in a head-to-head configuration and can be (re)-bound by RAG proteins (Figure 1A) (Curry et al., 2007; Neiditch et al., 2002). Synaptic complex formation between the ESC and genomic RSSs or cryptic RSSs (cRSSs) can result in re-integration of the ESC as evidenced by mouse lymphocytes being littered with re-inserted ESCs (Curry et al., 2007). However, our biochemical analysis of the re-integration reaction showed that remarkably, within the synaptic complex between the SJ and an RSS, the RSS is cut much more efficiently than the head-to-head arrangement of RSSs in the SJ. We find further that following cutting, the RSS is released, generating a double strand break while the RAG-SJ complex remains intact and is free to trigger cutting at further RSSs. We have named this reaction "cut-and-run" and propose that the ESC triggers a double strand break (DSB) at one RSS and then "runs," to potentially trigger breaks at additional RSSs. Consistent with this idea, we find that the frequency of broken RSSs increases significantly in the presence of the ESC and that these breaks

mirror those associated with chromosome translocations or interstitial deletions in lymphoid cancers.

RESULTS

The unique arrangement of two head-to-head RSSs in the SJ (Figure 1A) could potentially form a distinct complex with RAG proteins to influence ESC activity. To test if this is the case, RAG interactions with the SJ were examined *in vitro*, and we first asked if our RAG cutting conditions obey the 12/23 rule that ensures that efficient RAG cleavage occurs only between RSSs with different spacer lengths (reviewed by Schatz and Swanson, 2011). As expected, addition of an unlabeled partner RSS results in an ~10-fold increase in cutting at both 12- and 23-RSSs compared to the level of cutting in the absence of a partner RSS (Figure 1B, lanes 3 and 7).

Asymmetric Cutting within an SJ-RSS Synaptic Complex

The SJ has both a 12- and 23-RSS and therefore is expected to enhance cutting of either a 12- or 23-RSS. Consistent with this, we find that an unlabeled SJ oligonucleotide increases RAG cutting at both RSS types by a magnitude similar to when a single complementary RSS is added (Figure 1B, lanes 4 and 8). Remarkably, however, RAG cleavage of a SJ is low and is barely increased by addition of either a 12- or 23-RSS partner (Figure 1B, lanes 10–12). These data therefore suggest that within a 12- and 23- RSS complex, each RSS enhances cutting of the other, resulting in symmetric RAG cutting. By contrast, within a SJ-RSS complex asymmetric cleavage is observed since the RSS is cleaved significantly more than the SJ.

Asymmetric SJ-RSS cutting occurs over a range of concentrations of partner RSS (Figure S1A). Moreover, SJ-mediated stimulation of RSS cutting is specific since no increase in cleavage is observed upon addition of non-RSS containing DNA (Figure S1B). To test if reduced SJ cutting is due to limiting amounts of protein, RAG proteins were titrated over an 8-fold range. No substantial change in cutting at either a 12-RSS or a SJ was observed (Figure 1C). Likewise, to test if another component in the reaction is limiting, the DNA substrate was increased by 10-fold. A corresponding increase in SJ cutting was seen (Figure S1C), suggesting that no individual component is limiting; instead, something intrinsic to the SJ sequence appears to prevent its cutting.

Previous studies showed that RSS flanking sequences influence RAG cutting (Sadofsky et al., 1995). This does not explain reduced SJ cutting, however, because the flanking dinucleotide for each of the RSSs within the SJ is GT, which is cut efficiently. Therefore, to better understand this phenomenon, we next examined at which step of the RAG cutting reaction asymmetric SJ-RSS cutting occurs. RAGs first generate a nick precisely at the boundary between the heptamer and coding region; the free 3′-OH group then attacks the opposite DNA strand, forming a hairpin at the coding end and a blunt double strand break at the signal end (Figure 1A) (reviewed by Gellert, 2002). To test whether SJ cutting is blocked at the nicking or hairpinning step, RAG cleavage products were electrophoresed on a denaturing gel. Nicking and hairpinning occur efficiently at the 12- and 23-RSS when paired with either a complementary RSS or the SJ (Figure 1D, lanes 1–4 and 5–8). Nicking of the SJ, however, is markedly lower, both in the absence of partner and upon addition of a 12- or 23-RSS. Moreover, while a low level of hairpin formation is observed in the presence of a 12-RSS partner, hairpins are almost completely absent with a 23-RSS. Together, these data suggest that there is asymmetric cleavage within a SJ-RSS complex and that SJ cutting is primarily blocked at the nicking step but hairpin formation is also prevented in a SJ/23-RSS pair.

Asymmetric Cutting Is Observed with Different SJs

In the experiments described above, the SJ (SJ1) has two consensus RSSs in a head-to-head configuration. However, RSSs can differ from the consensus, especially at non-critical bases. The most conserved bases are the first three positions in the RSS heptamer and positions 5 and 6 of the nonamer (Ramsden et al., 1994). To test if asymmetric cutting also occurs with non-consensus SJs, two common alternative heptamer sequences, CACA**A**TG and CACAG**CC** (Ramsden et al., 1994) were substituted for one of the conserved heptamers in SJ1 to generate SJ2 and SJ3. As for SJ1, these new SJs stimulate cutting at both a 12- and 23-RSS (Figure S2B, lanes 5, 6, 11, and 12), while the SJ itself is poorly cut, even in the presence of partner RSSs (Figure S2C). Moreover, analysis of the ESCs generated from known recombination events predicts that 72% consist of RSSs that are conserved at six or seven positions in the heptamer and at positions 5 and 6 in the nonamer (Table S1). This implies that most of the human ESCs generated are likely to behave similarly to those tested here. Such asymmetric SJ-RSS cutting therefore raises the possibility that RAG-ESC complexes could trigger breaks throughout the genome at RSSs and cRSSs, to potentially generate substrates for chromosome alterations.

Although the SJ is consistently cut much less frequently than the RSS when these elements are present in *trans*, when they are present in *cis* on the same DNA construct, both elements are cut (Figure S2D). A likely explanation for this is the much faster kinetics of synaptic complex formation during an intra-molecular interaction that may result in cutting before RAGs are fully complexed with the SJ (below). *In vivo*, an SJ can be present in *cis* to RSSs following inversional recombination; if both the SJ and an RSS are in accessible chromatin, RAG cleavage could result in deletion of part of the antigen receptor locus. Deletional recombination to generate an ESC, however, is much more common than inversional recombination, resulting in the dangers outlined above.

Asymmetric Cutting Is Caused by RAG Binding to Both RSSs in the SJ

The SJ consists of two RSSs in a head-to-head configuration, and it seemed plausible that the molecular basis of the asymmetric cutting described above might stem from simultaneous RAG binding to both RSSs of the SJ to prevent cutting at the heptamer/heptamer boundary. Consistent with this, footprinting studies showed RAG contacts extend 12 bp into the coding flank in a single RAG-RSS complex and 16 bp in a paired complex (Nagawa et al., 2002, 2004). Contacts to at least 10 bp of coding flank, have been further confirmed by recent cryo electron

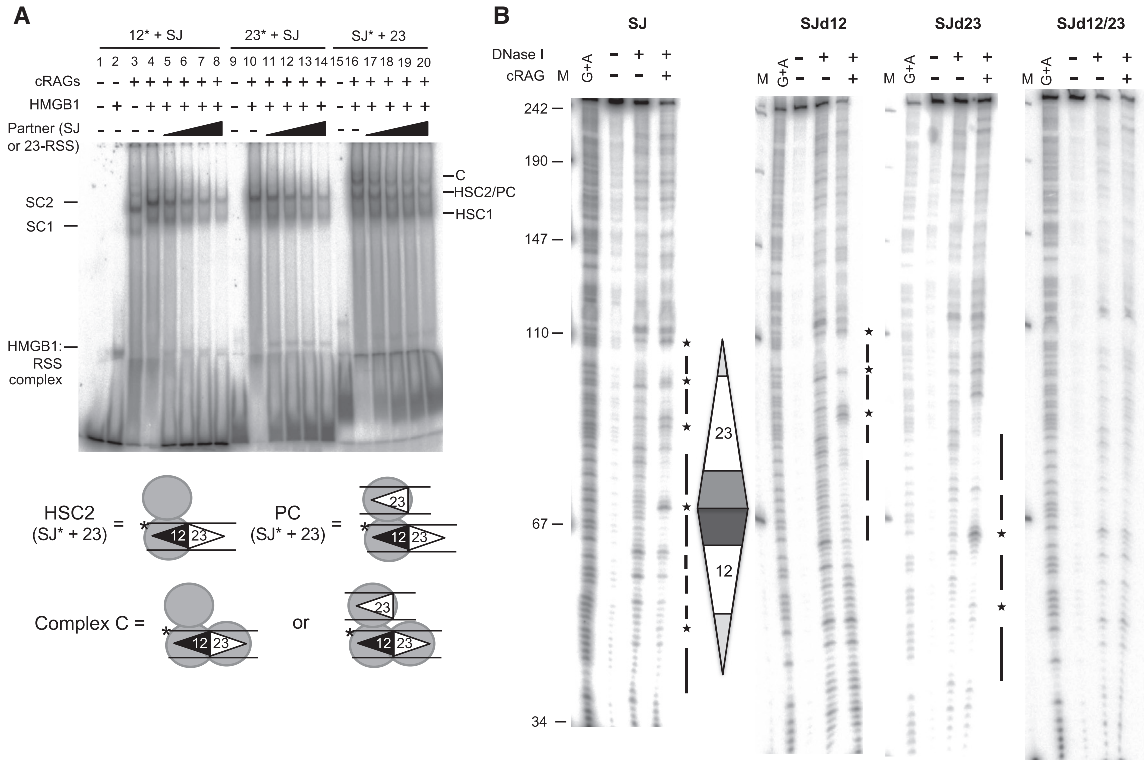

Figure 2. RAGs Bind to Both RSSs in the SJ

(A) A slower migrating complex is formed with the SJ. RAG complexes were formed with labeled oligonucleotides carrying a 12-RSS (lanes 3–8), 23-RSS (lanes 10–14), or SJ (lanes 16–20) and run on a 4% polyacrylamide gel. Increasing amounts of unlabeled partner RSSs are present as indicated above the gel. When the SJ is labeled, a third complex of higher molecular weight is visible (indicated by “C”). This is not visible in lanes containing unlabeled SJ, most likely because the paired complex has an average lifetime of ~400 s (Lovely et al., 2015) and would dissociate before loading on the gel. SC1/2, single complex 1/2; HSC1/2, HMGB1 single complex 1/2; PC, paired complex.

(B) DNaseI footprint shows both RSSs are occupied. The wild-type (WT) SJ is on the left and the position of the RSSs, as determined from the G+A marker ladder is shown to the right of this gel. Triangles indicate nonamer sequences and pentamers represent hexamers. Bars indicate protected regions; stars depict enhanced cutting. Footprints using SJ probes where the 12-RSS, 23-RSS, or both RSSs are mutated in heptamer and nonamer sequences are shown to the right. Protection of mutated heptamers adjacent to the WT RSS with SJd12 and SJd23 likely results from RAG binding to flanking DNA (Nagawa et al., 2002, 2004).

microscopy (EM) studies (Kim et al., 2018). These studies also demonstrated that DNA nicking by RAGs requires melting of the heptamer CAC and a 180° corkscrew rotation of the coding flank DNA to place the scissile phosphate in the active site (Kim et al., 2018; Ru et al., 2018). Such dynamic changes may be blocked when the coding flank is bound by a second RAG complex, thereby preventing cutting of the ESC.

To investigate this idea further, we compared the complexes formed on the SJ and RSS by a gel mobility shift assay. Previous studies have shown that RAG proteins form two complexes with an RSS, single complex 1 (SC1) that contains two molecules of RAG1 and one of RAG2 and single complex 2 (SC2) that contains two molecules each of RAG1 and RAG2 (Swanson, 2002). HMGB1 is present in all our reactions to aid cleavage (van Gent et al., 1997) and in the presence of HMGB1, these complexes are supershifted to form HSC1 and HSC2 (Swanson, 2002). However, with the SJ, an additional, higher molecular weight complex is formed (complex C, Figure 2A, lanes 16–20). Given that the only difference between the SJ and 12- and 23-RSS oligonucleotides is the presence of an additional RSS in the SJ, these data suggest that this slower migrating complex may result from additional RAG binding to the second RSS.

To further test if RAGs indeed bind to both RSSs of the SJ, we carried out DNase I footprinting studies by binding purified core RAG proteins to a labeled DNA fragment that carries a consensus SJ sequence. Equivalent protected regions are detected on both RSSs of the SJ (Figure 2B, left), and because some sites are fully protected on each RSS, this implies that both RSSs of the SJ are bound simultaneously by RAGs. Control experiments where the heptamer and nonamer sequences of each RSS are mutated (Figure 2B, middle and right) confirm that the observed protection is a result of RAG binding.

Mutating the Second RSS in the SJ Restores Symmetric Cutting

If asymmetric cutting is indeed due to simultaneous RAG binding to both RSSs of the SJ, then we would predict that mutations that abolish RAG binding to one RSS of the SJ will result in increased cutting of the SJ in the presence of an appropriate partner RSS.

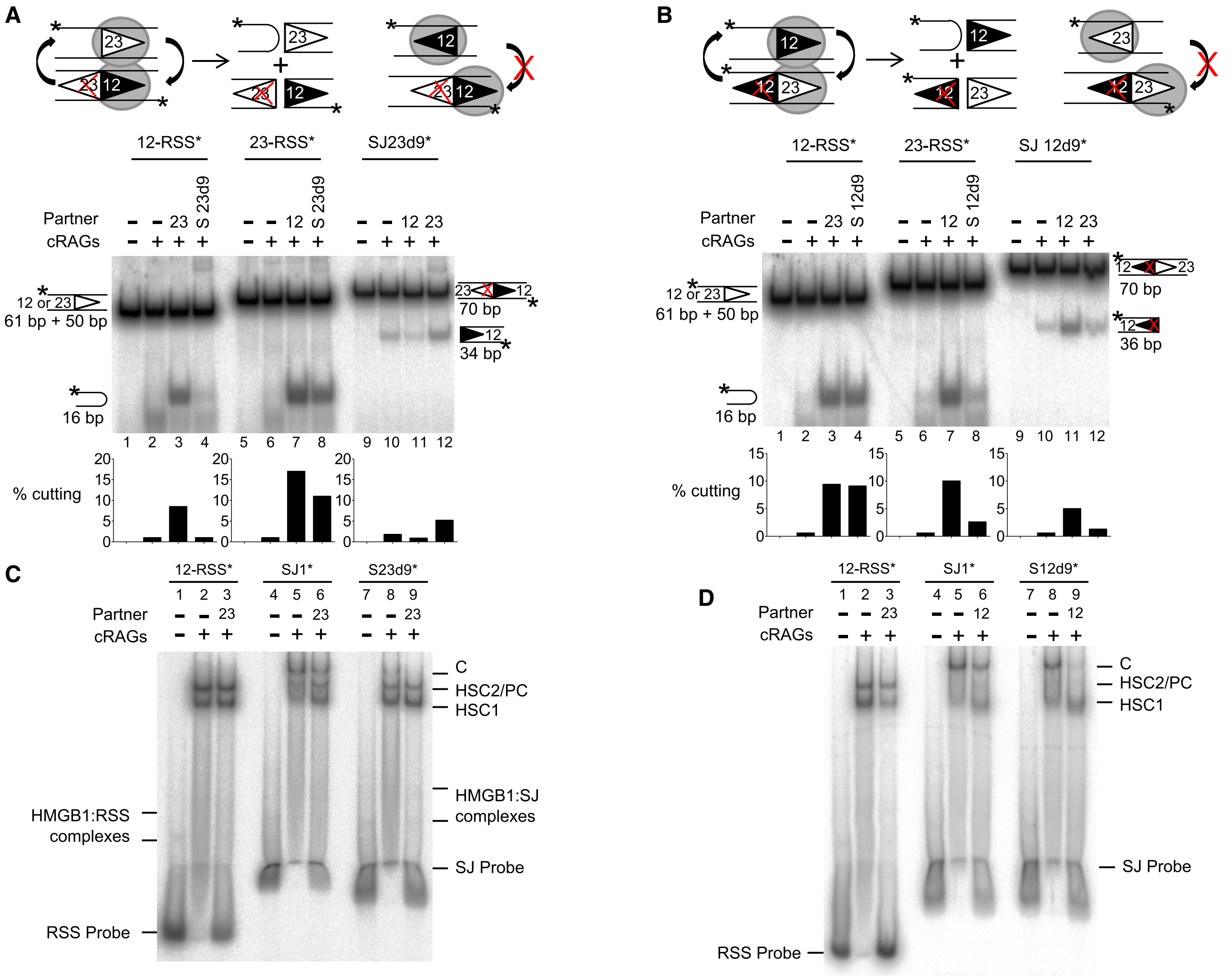

Figure 3. Mutating One RSS within the SJ Restores Symmetric Cleavage

(A) The 23-RSS nonamer within the SJ was deleted and the oligonucleotide used in cutting reactions either as a partner for labeled 12- or 23-RSSs or as the substrate. Symmetric cutting was almost restored when SJ23d9 was paired with a 23-RSS.

(B) Deletion of the nonamer within the 12-RSS of the SJ results in more symmetric cutting with a 12-RSS. As for (A) except the oligonucleotide SJ12d9 was used.

(C and D) RAG binding to mutant SJs. RAG complexes were formed with the 12-RSS, wild-type SJ, or mutant SJs and resolved on a native gel. Complex "C" is formed with the wild-type SJ but is reduced with SJ23d9 and SJ12d9. This is most pronounced in the presence of a partner RSS that likely outcompetes weaker RAG binding to the mutant SJ.

See also Figure S3.

To test this idea, we used an oligonucleotide in which the nonamer of the 23-RSS within the SJ is mutated (SJ23d9). This retains a consensus 12-RSS and thus is expected to promote cutting when paired with a 23-RSS. This prediction is borne out with increased cutting of a 23-RSS but not a 12-RSS, in the presence of SJ23d9 (Figure 3A, compare lanes 4 and 8). Moreover, when an unlabeled partner 23-RSS is added to the labeled SJ23d9 oligonucleotide, a clear increase in SJ23d9 cutting is observed (Figure 3A, lane 12). Similarly, when the 12-RSS nonamer (Figure 3B) or the 12-RSS heptamer (Figure S3) of the SJ are mutated, increased cutting is observed when the mutated SJ is paired with a 12-RSS, resulting in almost symmetrical cleavage within the SJ-RSS complex. These data are therefore consistent with the idea that when RAGs bind to both sides of the SJ, this prevents SJ cleavage within an SJ-RSS complex.

Our model also predicts that if RAG binding to both RSSs of the SJs is represented by the higher molecular weight complex C seen in Figure 2A, then mutating one of the RSSs would be expected to reduce the abundance of this complex. Consistent with this, we find deletion of the nonamer of the 23- or 12-RSS in the SJ reduces formation of complex C compared to the unmutated SJ in the presence of competitor (Figures 3C and 3D). Overall, therefore, these data support the model that RAG proteins bind simultaneously to both sides of the SJ, leading to asymmetric cleavage.

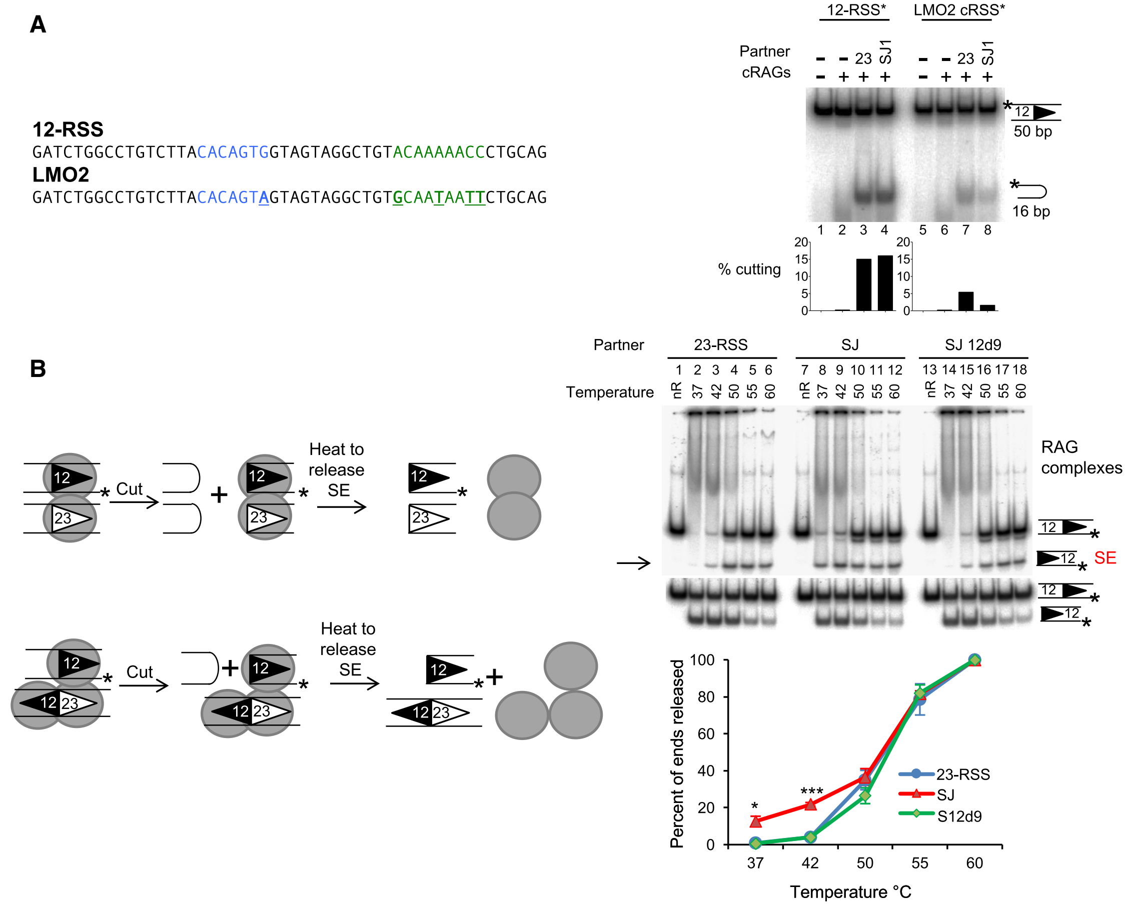

Figure 4. The ESC Poses a Risk to Genome Stability

(A) The SJ triggers cutting at cRSSs. RAG cutting assays were performed using radiolabeled oligonucleotides, denoted by an asterisk, carrying a 12-RSS or the LMO2 cRSS. Unlabeled 23-RSS or SJ partners are present as indicated.

(B) Signal ends are released faster following a SJ-RSS cutting reaction than a 12/23-RSS reaction. Cutting reactions, using oligonucleotides labeled on the lower strand to detect signal ends, were performed at 37°C, followed by incubation at the temperatures indicated. The reaction was divided into two and resolved on a native gel (top) and a cutting gel (bottom). Release of the signal ends (indicated by the arrow, top gel) was calculated relative to the level of cutting (bottom gel). Data obtained with core RAG1 and full-length RAG2 are shown; equivalent data were obtained with core RAG2. nR = no RAGs. Data are represented as mean of three experiments ± SD. Increased SE release in the presence of the SJ is statistically significant (Student's t test; *p = 0.01 [37°C] and ***p = 0.0001 [42°C]). See also Figure S4.

The SJ Triggers Cutting at a cRSS

The observed asymmetric cleavage between SJ-RSS complexes implies that the RAG-ESC complex could trigger DSBs at RSSs *in vivo*. The risks associated with this to genome stability will be determined, in part, by the locations of the ESC-directed breaks. These could occur at single RSSs in the antigen receptor loci to produce substrates for end donation reactions as well as at cRSSs, some of which lie next to oncogenes that are involved in chromosome translocations (Marculescu et al., 2002; Raghavan et al., 2001). To test if the SJ is capable of causing breaks at these oncogene-associated cRSSs, the LMO2 cRSS was used as an example. We find that cutting is increased in the presence of either a 23-RSS or SJ partner (Figure 4A, right), suggesting that the ESC can indeed promote RAG cutting at cRSSs, a finding that is confirmed by more extensive genome-wide analyses of RAG-SJ cutting (below).

The RSS Is Released following Cutting but RAGs Remain Bound to the SJ

A second factor that determines the danger posed by asymmetric cutting is the fate of the DNA following cleavage. If, following cutting, the broken RSS is released, then RAG proteins would be unable to shepherd the broken end toward the cNHEJ pathway (Lee et al., 2004), thereby enhancing the risk of translocations. To investigate the fate of the broken RSS, we determined the stability of the RAG:RSS post-cleavage complexes. First, the release of signal ends was quantified by challenging complex stability at increasing temperatures (Figure 4B) (Coussens et al., 2013) and second, the release of coding ends was measured, using time course assays (Figure S4). In both cases, half of the reaction was resolved on a native polyacrylamide gel to analyze RAG-substrate complexes, and the other half was deproteinated to analyze RAG cleavage products on cutting gels.

We find that the labeled signal end is released at lower temperatures in the presence of a SJ partner (Figure 4B, lanes 7–12), compared to complexes involving either an RSS or mutant SJ partner (Figure 4B, lanes 1–6 and 13–18). Likewise, when coding ends are examined, HSC1 and HSC2 are formed with a labeled 12- or 23-RSS (Figures S4A and S4B), but upon addition of a partner, RSS cleavage is stimulated and this correlates with a substantial loss of HSC2, the active cleavage complex (Figures S4A and S4B, middle and right). By contrast, following addition of an RSS partner to a SJ, the levels of SJ complexes remain relatively unchanged (Figure S4C). These data therefore indicate that a SJ-stimulated cleavage of an RSS results in the release of the signal and coding ends, but RAG proteins remain stably associated with the SJ. This implies that the cut RSS is free to undergo joining with other DNA ends, while the RAG-ESC complex could potentially trigger further DNA breaks at additional RSSs, via a cut-and-run mechanism.

Asymmetry of SJ-RSS Cutting *In Vivo*

To further test the cut-and-run idea, we next investigated if asymmetric RAG cutting within the SJ-RSS synaptic complex is also observed *in vivo*. To this end, integrase-deficient lentiviruses carrying a 12-RSS, a 23-RSS, or a SJ were used to transduce REH cells, a B cell line derived from an ALL patient (Rosenfeld et al., 1977) where RAG expression is at physiological levels (Bories et al., 1991). 48 h after transduction, episomal DNA, resulting from reverse transcription and homologous recombination between the LTRs of the integrase-deficient lentiviruses, was recovered. The level of RAG cleavage of the RSS or SJ on these episomes was then measured via qPCR, using a primer pair that amplifies across the RAG cleavage site so that only intact substrate is amplified. The data were normalized by amplifying a sequence unique to each substrate.

When lentiviruses carrying either a 12- or a 23-RSS were cotransduced into REH cells, so stimulation of RAG cleavage requires synapsis in *trans*, the amount of intact 12- and 23-RSS substrate was reduced to 70% and 74%, respectively, relative to samples where no partner is present (Figure 5A, left). This is consistent with the level of cutting previously published for cutting in *trans* using vectors with individual RSSs (Steen et al., 1996). When a SJ-carrying lentivirus is transduced with either a 12- or 23-RSS, in each case cutting occurs at the RSS, while negligible cleavage of the SJ is detected (Figure 5A). Therefore, *in vivo*, as *in vitro*, cleavage within a SJ-RSS complex is asymmetric. Such asymmetric cutting was also observed in NIH 3T3 cells transfected with expression vectors for RAG1 and RAG2, together with two substrate plasmids, that encode either a 12-RSS, a 23-RSS or a SJ (Figure S5A). Notably, asymmetric cutting was observed regardless of whether core RAG2 or full-length RAG2 protein was used, implying that the non-core region of RAG2, which is known to inhibit transposition and re-integration reactions (Curry et al., 2007; Elkin et al., 2003), does not influence asymmetric SJ-RSS cleavage (Figure S5B).

Our *in vitro* data suggest that asymmetric cutting in the SJ-RSS synaptic complex results from RAG binding to both RSSs in the SJ simultaneously. To test if the same is true *in vivo*, we used the same mutated SJ sequences as above in the REH cell/virus transduction assay, together with wild-type RSS vectors. If RAG binding to the second RSS indeed blocks SJ cutting, then we predict that the mutated SJ should be cut in the presence of the relevant RSS partner. As can be seen in Figure 5A, this prediction is borne out, both for the mutant SJ/12-RSS pairs (Figure 5A, middle) and also when a SJ mutated at the 23-RSS is paired with a 23-RSS (Figure 5A, right). Moreover, the same effect is observed in transfected NIH 3T3 cells (Figure S5A). Together, these data imply that asymmetric cleavage within a SJ-RSS synaptic complex occurs via the same mechanism *in vivo* as *in vitro*.

RAGs generate blunt double strand breaks at the signal ends and to verify that the observed cutting was RAG-mediated, the recovered DNA samples were subject to ligation-mediated polymerase chain reaction (LM-PCR). Here, a linker that recreates an ApaLI site upon ligation to a broken signal end was used to allow the presence of RAG-cleaved ends to be verified by ApaLI digestion. As can be seen in Figure 5B, the reduction in intact 12- and 23-RSSs in Figure 5A as well as mutated SJs is mirrored by an increase in LM-PCR product, indicating that RAG cleavage indeed appears to be involved. By contrast, only minimal cutting of the unmutated SJ is detected (Figure 5B, middle and right). Similar analyses confirmed DSBs are present at RSSs in NIH 3T3 cells whereas cutting of the SJ was undetectable (Figure S5B).

The ESC Is Ligated *In Vivo*

The ESC has been reported to persist *in vivo* with unligated signal ends (Livak and Schatz, 1996; Ramsden and Gellert, 1995), either because these ends are ligated more slowly than coding ends or because the ESC is re-cut by RAG proteins (Neiditch et al., 2002). Because these "open" ESCs could potentially undergo re-integration, rather than cut-and-run, we were keen to assess the level of unligated signal ends *in vivo*. To this end, we examined the Igλ locus where 60%–70% of recombination occurs between Vλ1 and JCλ1 (Boudinot et al., 1994), and thus the Vλ1Jλ1 ESC is generated in the majority of Igλ-positive cells. Using LM-PCR, we find that unligated Vλ1Jλ1 signal ends are detectable, but only weakly (Figure 5C). To determine the proportion of signal ends that are fully ligated, we separately amplified the Vλ1Jλ1 ESC signal joint and the Vλ1Jλ1 coding joint (Figure 5D, red arrows and blue arrows, respectively) and then prepared a dilution range with known numbers of copies of each PCR product. These were used as standard curves in qPCR reactions to determine the absolute amounts of coding and signal joints in pre-B DNA samples. This showed that ESC signal joints are present at 60.4% the level of the Vλ1Jλ1 coding joints. Similarly, we find that 52% and 65% of the Vκ16Jκ5 and Vκ3Jκ5 ESCs, respectively, are intact. Together, these data imply that a majority of ESCs are ligated in pre-B cells *in vivo* and thus have the potential to undergo cut-and-run (Figure 5D).

ESCs Cause Increased DSBs *In Vivo*

If the cut-and-run hypothesis is correct, we expect that the number of DSBs *in vivo* will increase in the presence of the RAG-ESC complex. Within a minute of DSB formation, γH2AX becomes phosphorylated at serine 139, which provides an excellent marker for genomic DSBs (Rogakou et al., 1998). To test if the ESC indeed triggers DSBs, we first transfected Cos7 cells with

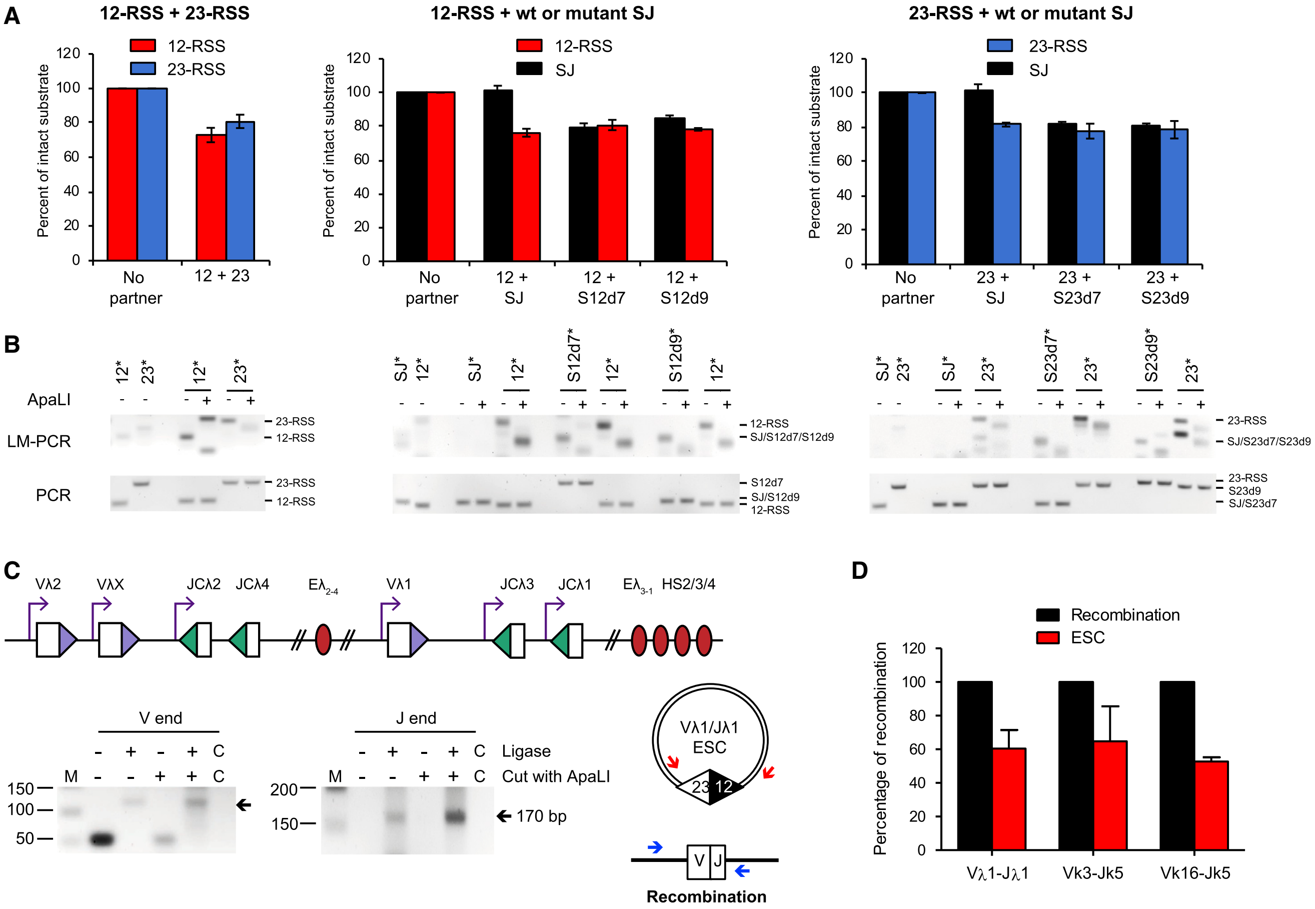

Figure 5. Cleavage of SJ-RSS Substrates Is Asymmetric *In Vivo*

(A) REH cells were transduced with integrase-deficient lentiviruses carrying a 12-RSS, a 23-RSS, or a wild-type or mutant SJ sequence, as indicated. The amount of intact substrate after 48 h was measured by qPCR. Data were normalized to unique regions within each provirus, and the values given are relative to controls where lentiviruses carrying a partner sequence were not transduced. Data are represented as mean of three separate transductions ± SD. See also Figure S5.

(B) LM-PCR was performed using the samples from (A), and the products are shown under the respective graphs. The template amplified by LM-PCR is indicated above the lanes with an asterisk. Ligation of the linker primer to a RAG cleaved end recreates an ApaLI site and digestion was used to verify RAG-mediated cutting. For the SJ mutations (and SJ), the RSS that is expected to be blunt following cleavage was amplified by LM-PCR. Amplification of each of the SJ ends is in the quantitative range and since the same primers were used to amplify the SJ-12-RSS end and SJ23d7, the increased signal with SJ23d7 indicates increased cutting, likewise for the SJ-23-RSS end and SJ12d9. SJ12d7 and SJ23d9 were cloned into the vector in the reverse orientation and gave different sized products with some primers. This also resulted in cross-reactivity of SJ23d9 with 23-RSS primers, giving an additional band (right gel, lane 13). Samples were normalized using qPCR; the bottom (PCR) shows PCRs with the normalization primers to verify the respective vectors were present.

(C) Unligated signal ends are present *in vivo*. Top: cartoon of the Igλ locus. Bottom: DNA from pro-B cells of PIP3 transgenic mice, where the Igλ locus undergoes premature recombination in pro-B cells, was used in an LM-PCR reaction to amplify Vλ1 and Jλ1 signal ends. ApaLI was used to cleave the signal ends prior to LM-PCR to give a positive control for unligated signal ends. No ligase controls are shown; C indicates the no template control.

(D) The majority of signal ends are ligated. The signal joints and coding joints of three distinct ESCs or recombination products were quantified against a standard curve of known numbers of copies and the relative amounts calculated. Data are represented as mean of three experiments ± SD.

a plasmid carrying a SJ and an SV40 origin of replication to increase the number of SJs present. Cotransfection with RAG expression plasmids resulted in a mean of 7.3 γH2AX foci per cell compared to far fewer in control cells where the RAG expression vectors, or the SJ vector, were omitted or where a 12- or 23-RSS or SJ12d9 replaced the SJ (Figure 6A). This implies that the RAG-ESC complex indeed triggers genomic DSBs.

To examine whether the SJ also triggers DSBs at physiological RAG concentrations, REH cells were transduced with a lentivirus carrying a SJ, a single RSS, a mutated SJ (SJ12d9) or a control (empty) virus and γH2AX foci were measured. Although only 3–6 SJ vectors are present per cell (STAR Methods), a significant increase in foci was again observed with the SJ but not the single RSS or mutated SJ (Figure 6B).

We noticed a higher level of cell death in cells transduced with the SJ-carrying lentivirus compared to those transduced with a control virus. Because unrepaired DSBs trigger apoptosis via the p53-mediated pathway, we next examined the levels of apoptosis by staining REH cells with fluorescent annexin V, which detects early apoptosis. A significant increase in staining was observed in the cells transduced with a SJ-containing virus compared to a single RSS or the control virus (Figure 7A), which

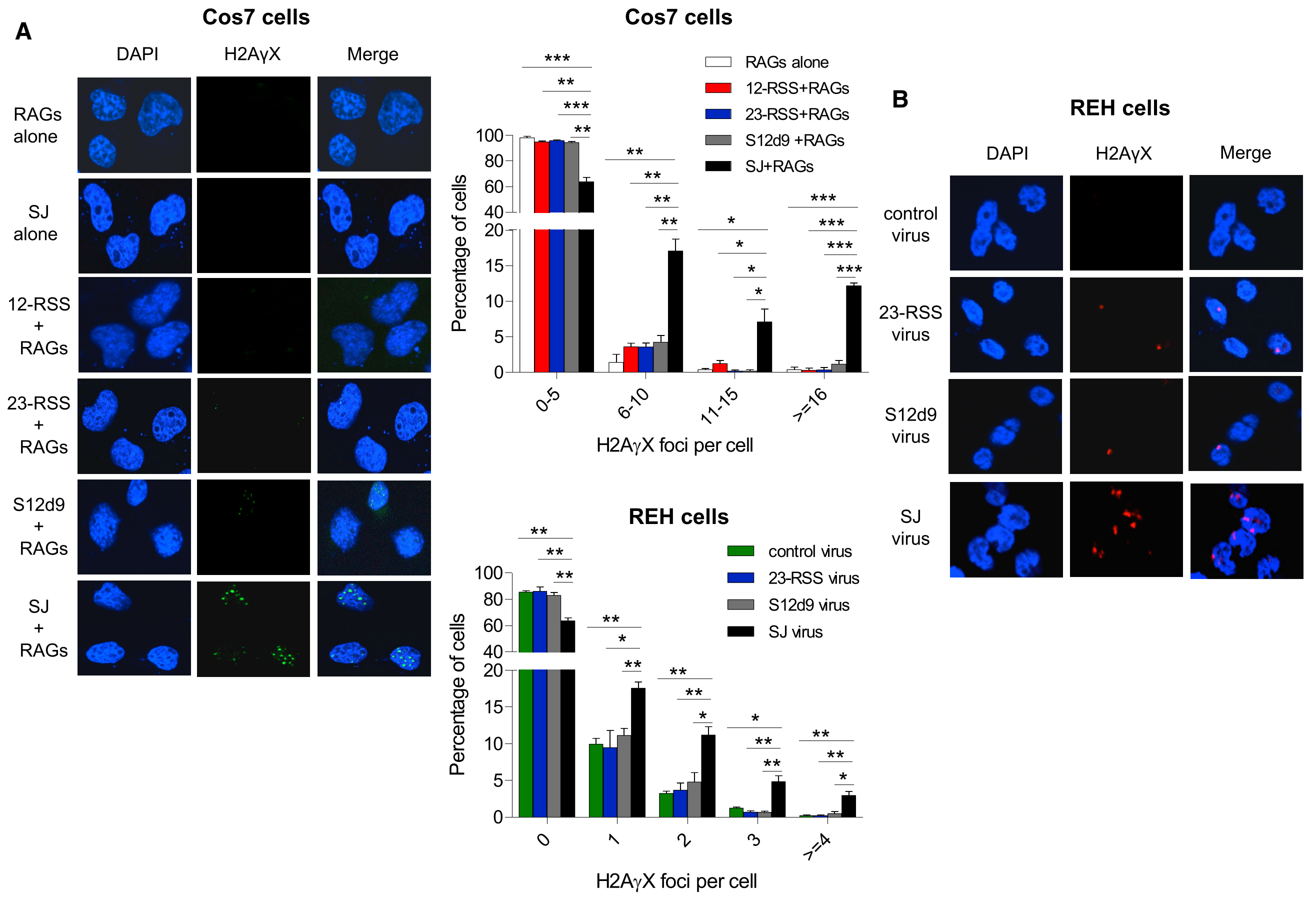

Figure 6. The SJ Triggers DSB *In Vivo*

(A) Left: γH2AX foci in Cos7 cells transfected with RAG expression vectors and plasmids carrying the 12- or 23-RSS or SJ12d9 or SJ. One representative example of a vector alone transfection is shown. Middle, top graph: quantification of γH2AX foci from 450 Cos7 cells per transfection condition and three independent experiments.

(B) REH cells were transduced with EGFP-expressing integrase-deficient lentiviruses, that carry no additional sequence (control virus), the 23-RSS, SJ12d9, or the SJ sequence. γH2AX foci were detected using an AlexaFluor568-labeled (red) secondary antibody. Middle, bottom graph: foci in 450 REH cells per condition from three independent experiments. Fewer foci are observed than in (A) because only 3–6 RSS or SJ vectors are present per cell. Error bars show SD.

is consistent with the generation of increased DNA breaks in the presence of the ESC, as expected for the cut-and-run reaction. In the presence of SJ12d9, more apoptotic cells are present than with a single RSS, which is likely due to residual RAG binding to both RSSs in SJ12d9 (Figure 3D). The levels, however, remain significantly below those with the wild-type SJ.

We next wished to test if the DSBs triggered by the RAG-ESC complex occur at RSSs, as would be predicted by the cut-and-run hypothesis, and also if the broken ends potentially form the substrates for chromosome translocations or deletions. We therefore performed linear amplification-mediated high-throughput genome-wide translocation sequencing (LAM-HTGTS) by transducing REH cells with lentiviruses designed to introduce a CRISPR/Cas9-mediated "bait" DSB at genomic loci and asked if there are increased translocations of "prey" DSBs to these sites in the presence of the SJ.

Two separate loci were chosen to introduce the Cas9-mediated breaks: *ROSA26* and *IgH*. We then determined the breakpoints that are translocated to the Cas9-mediated breaks using the LAM-HTGTS software pipeline and the DNAGrab algorithm to predict whether RSSs are present (RSS site; https://www.itb.cnr.it/rss/analyze.html). A significant increase in translocations involving broken RSSs is observed in cells transduced with the SJ-containing lentivirus compared to those transduced with Cas9-only virus ($p < 0.0001$; Figure S6A) or with control empty virus (*IgH*; $p < 0.0001$, *ROSA*; $p = 0.0004$) or with viruses carrying a single RSS ($p = <0.0001$ for both 12- and 23-RSS). Given that the 23-RSS sequence is the same in the 23-RSS and SJ lentiviruses and that these viruses differ by <300 out of 6,198 bp, the increased breaks at genomic RSSs in the presence of SJ is quite remarkable. We do, however, observe a similar level of cutting at RSSs following transduction with lentiviruses containing SJ12d9 and SJ. This might be because these two vectors differ by only 7 bp and there is some residual RAG binding to both RSSs in SJ12d9 (Figure 3D). Notably, the LAM-HTGTS assay measures just the first round of cutting and, unlike the γH2AX assay, cannot detect subsequent cuts. This can potentially explain why cutting at RSSs appears similar in the

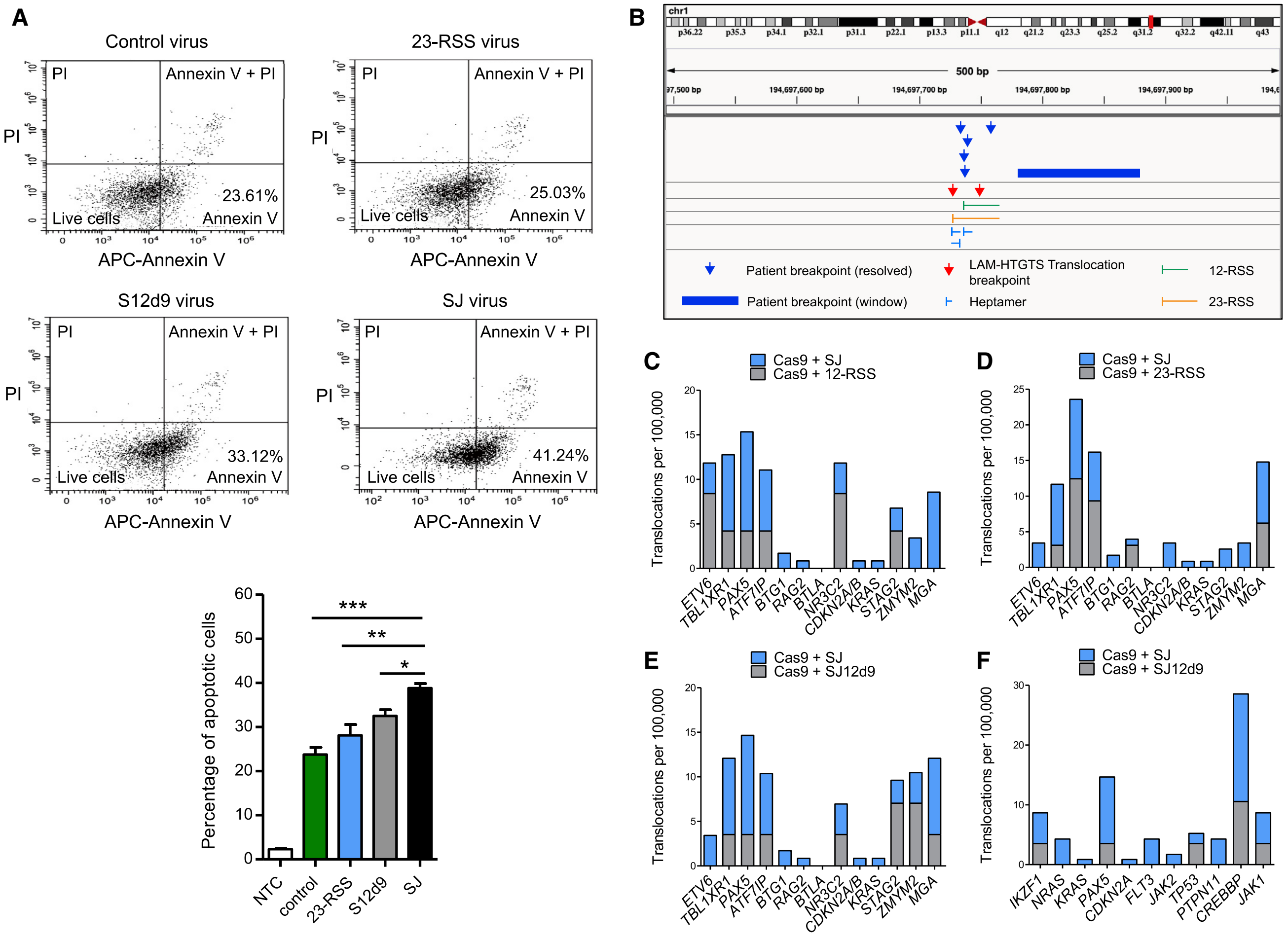

Figure 7. The SJ Causes Chromosome Breaks

(A) Apoptosis in transduced REH cells as measured by annexin V and propidium iodide (PI) staining for early and late stage apoptosis, respectively. Data are represented as mean of three independent experiments ± SD; increased early stage apoptosis in the presence of the SJ compared to the other vectors is statistically significant (Student's t test; ***p = 0.0002 [control vector], **p = 0.0064 [23-RSS], *p = 0.011 [SJ12d9]).

(B) Example of two SJ-mediated breaks that co-localize with breaks found in ALL patients. Chromosome breaks mapping close to the cRSS at 194697727 on chromosome 1 were observed in six ALL patients and in two independent LAM-HTGTS experiments. Similar co-localization was observed for 22 more patients (see also Figures S6, S7, and Table S2).

(C–E) Graphs showing the frequency of breaks in the 13 genes that most frequently acquire somatic mutations in *ETV6/RUNX1*-positive ALL (Papaemmanuil et al., 2014) in the presence of the SJ compared to the 12-RSS (C), 23-RSS (D), and SJ12d9 (E), respectively.

(F) As for (E) but comparing the frequency of breaks in genes that are commonly mutated in all types of B-ALL.

See also Table S3.

LAM-HTGTS assay with the SJ and SJ12d9 (Figure S6) but is very different in the γH2AX assay.

Previous whole genome sequencing studies of *ETV6/RUNX1*-positive ALL patients identified 140 chromosome breakpoints that lie in close proximity to RSS-like sequences. Crucially, we find breaks that are generated in the presence of the SJ map to within a 100 bp window of 28 different ALL patient breakpoints (Table S2) (Papaemmanuil et al., 2014). The probability of this degree of overlap if the breaks were randomly located is one in 2×10^{12} ($p = 6 \times 10^{-13}$). It is also notable that three of the breaks identified with the SJ occur in 5, 5, and 6 patients, respectively, suggesting that these might be hotspots for chromosome alterations in ALL (Figures 7B and S7). By contrast, there is no significant increase in the degree of overlap of the breaks generated in the presence of control virus (p = 0.7), Cas9 (p = 0.07), or the 12-RSS (p = 0.6). We do, however, find four of the translocations with the 23-RSS and SJ12d9 overlap with patient breakpoints (p = 0.002), which is consistent with previous studies where single RSSs can cause some genome instability (Han et al., 1999). That said, one of the breaks identified with SJ12d9 maps precisely to a breakpoint detected twice with Cas9, suggesting that it might be due to off-target Cas9 cutting.

Finally, previous whole genome sequencing and array-based genome profiling studies identified genes that are commonly mutated in *ETV6/RUNX1*-positive ALL. These genes are often involved in B cell development and of these, 13 were found

to be enriched in RSS motifs and acquire somatic mutations most frequently (Papaemmanuil et al., 2014). Our LAM-HTGTS assay detects an increase in cleavage of nine of these genes in the presence of the SJ-containing virus that is a significant increase in the number of genes cleaved (p = 0.01–0.02), compared to the number of genes cleaved with the single RSSs or SJ12d9 virus (Figures 7C–7E; Table S3). Furthermore, using the list of genes that are commonly mutated in all types of B-ALL from the COSMIC database (https://cancer.sanger.ac.uk/cosmic) we find increased breaks in eight to ten of the eleven most commonly mutated genes in the presence of the SJ (p = 0.02–0.0002) compared to breaks with the single RSSs or SJ12d9 (Figure 7F; Table S3). These data therefore strongly imply that cut-and-run makes a significant contribution to mutation of cancer genes and to chromosome alterations in ALL.

DISCUSSION

The excision of intervening DNA from between the gene segments to form an ESC by-product is an integral part of the V(D)J recombination reaction. Sealing the two signal ends was previously thought to sequester the potentially reactive DNA ends to inactivate them (Arnal and Roth, 2007). However, two reports published in 2007 indicated that ESCs are far from inert and can be re-bound by RAG proteins by virtue of the two RSSs and reintegrated back into the genome at RSSs or cRSSs (Curry et al., 2007; Vanura et al., 2007). While re-integration clearly occurs (Curry et al., 2007), we find that upon formation of a SJ-RSS synaptic complex, cleavage is asymmetric both *in vitro* and *in vivo*. Because following cleavage, the cut RSS is released but RAGs remain bound to the SJ, we propose that the RAG-ESC complex could trigger DSBs at further RSSs and cRSSs. This could continue until the ESC becomes cleaved, or until the RAG proteins are downregulated, thus generating multiple genomic DSBs in developing lymphocytes in a reaction we have named cut-and-run.

A plausible explanation for the observed asymmetric cleavage within the SJ-RSS synaptic complex is that RAG proteins bind to both RSSs of the SJ simultaneously, thus blocking effective binding of the heptamer-heptamer border and preventing efficient cleavage. This prediction was borne out by EMSA experiments that showed that a complex of higher molecular weight is formed with a SJ and by cleavage experiments with mutant SJs, which showed that deleting part of the conserved region on one side of the SJ restores symmetric cleavage by preventing simultaneous RAG binding.

We find that RAG cutting of a SJ is blocked at the nicking step although the low level of nicking seen at the SJ implies that RAG activity is not blocked entirely, but instead, the complex may have some intrinsic flexibility. Synapsis with a 12-RSS partially relieves the block because there is an increase in hairpin formation. By contrast, synapsis with a 23-RSS does not allow hairpin formation. The reason for this block is unclear but it is consistent with an *in vivo* study that found reintegration at Vκ 12-RSSs but not Jκ 23-RSSs (Curry et al., 2007) and may be due to the inability of a RAG/ESC/23-RSS complex to adopt the correct conformation for ESC cleavage.

The low level of SJ cleavage that we observe differs from previous studies where SJs were cut only ~3-fold less efficiently than RSSs (Neiditch et al., 2002). The difference from our data cannot be explained by the SJ sequence used because we confirmed our observation with six different SJs, including the one used previously. Instead, Neiditch et al. (2002) propose efficient SJ cleavage occurs by a nick-nick mechanism where RAGs sequentially nick at the heptamer boundary of each RSS to generate a DSB. Consistent with this, or with the failure to join signal ends until RAGs are downregulated, RAG1 and RAG2 binding maps precisely to broken signal ends detected by END-seq in thymocytes at the TCRα locus (Canela et al., 2016). By contrast, we find three different ESCs are ~60% uncut in primary pro- and pre-B cells (Figure 5D), although it remains to be determined whether RAGs normally associate with ligated ESCs.

Occurrence of Cut-and-Run *In Vivo*

Definitive proof that cut-and-run occurs *in vivo* is inherently difficult to obtain. Unlike chromosome translocations and reintegration, which result in gross DNA rearrangements and insertions, cut-and-run would leave no trace other than a broken RSS. If the cleaved RSS was subsequently used in a chromosome translocation, again there would be no indication that the breakpoint was specifically caused by an ESC. Nevertheless, several pieces of evidence suggest that it is highly likely that cut-and-run occurs *in vivo*.

First, extrachromosomal substrate assays indicate that cleavage of RSSs and SJs is asymmetric *in vivo* with both full-length and core RAG proteins, and therefore is not an *in vitro* artifact. Moreover, transduction of REH cells confirmed that asymmetric cutting occurs at physiological levels of RAG proteins and at only 3–6 copies of the SJ per cell.

In addition to asymmetric cleavage, cut-and-run requires the RAG-ESC complex to synapse with genomic RSSs. Reintegration of an ESC at genomic loci indicates that this is likely to be the case (Curry et al., 2007). Moreover, the increase in γH2AX foci in the presence of RAG proteins and the SJ strongly indicates that the RAG-ESC complex is capable of triggering DSB formation *in vivo*. Importantly, the LAM-HTGTS assay shows that this cutting occurs at cRSSs and that broken cRSSs are translocated to a DSB with a significantly higher frequency in the presence of the SJ than the 12- or 23-RSS alone.

While these data indicate that cut-and-run can occur *in vivo* and triggers DSBs, in order for ESCs to pose a threat during normal lymphocyte development, they must be present in a cell at the same time as RAG proteins. Previous studies indicated that signal ends are tightly bound by RAGs and may not form ESCs until RAGs are downregulated (Agrawal and Schatz, 1997; Livak and Schatz, 1996). Our analysis of the endogenous Vλ1Jλ1 ESC and Igκ ESCs in mouse pro- and pre-B cells, however, indicates that a substantial fraction of signal ends are ligated soon after they are formed. Moreover, there are several other instances where RAG expression could coincide with the presence of ESCs. The first is following rearrangement of the IgH or TCRβ locus; here, the ESC generated during rearrangement of the first locus in pro-B or pro-T cells will persist to later stages of lymphocyte development when RAGs are upregulated

to rearrange IgL or TCRα loci. Second, ESCs have been shown to persist for many weeks, even years, and upregulation of RAGs in immature B cells for receptor editing (Hillion et al., 2007) could enable RAG-ESC binding, potentially activating the cut-and-run reaction. Nonetheless, the times that RAGs and ESCs co-exist during lymphocyte development are relatively limited, which will restrict the frequency of the cut-and-run reaction.

Following RAG cleavage, the cut RSS is released and the dangers posed by these broken ends to genome stability depends entirely on their fate. In particular, it is unclear how asymmetric cleavage, that produces only one hairpinned and one blunt end, rather than a pair of each type of end, might affect the end-joining process. The broken RSS might be bound by the cNHEJ machinery and in the absence of a suitable partner, it may be more likely to undergo translocation. Alternatively, the DSB might be transferred to the alternative NHEJ pathway, or perhaps it is not transferred to any end-joining machinery at all. This would be consistent with studies that showed RAG proteins do not efficiently shepherd single broken ends to the cNHEJ pathway (Cui and Meek, 2007). More thorough analyses of the fate of the ends following RAG-ESC cleavage are needed to address the risks posed by cut-and-run.

Implications of Cut-and-Run

Chromosome translocations caused by aberrant recombinase activity in the production of antibodies and T cell receptors are a hallmark of many leukemias and lymphomas (Küppers, 2005). Here, we have identified an unexpected way in which aberrant RAG activity could produce orphan DSBs that have the potential to participate in carcinogenic chromosome translocations.

One of the most common forms of translocation caused by V(D)J recombination is end donation (Roth, 2003), and it is possible that cut-and-run produces the broken RSSs that serve as substrates for this reaction. Likewise, recent studies showed a high frequency of translocations of RSS and cRSS ends to an engineered DSB (Rommel et al., 2017). Although the use of core RAG proteins in these experiments could have enhanced the release of DNA ends (Coussens et al., 2013), translocations were also observed using full-length RAG proteins. A potential mechanism by which the broken RSS and cRSS are generated, is cut-and-run.

A specific leukemia where cut-and-run could play a significant role is ALL where patients bear the *ETV6/RUNX1* (*TEL/AML1*) translocation. This translocation often arises in utero, but it cannot trigger malignancy in the absence of secondary mutations. Instead, the *ETV6/RUNX1* translocation partially stalls B cell progression at the pre-B cell stage, where RAG proteins are still expressed (Tsuzuki et al., 2004), thus providing an extended window of opportunity for secondary recombination reactions. The majority of these aberrant recombination reactions occur at cryptic RSSs adjacent to transcriptionally active B cell development genes, which can eventually result in full malignant transformation; 50% of the RAG-mediated chromosome aberrations contain an RSS or cRSS at only one side of translocation (Papaemmanuil et al., 2014). Remarkably, our LAM-HTGTS data show that translocation break-points, formed in the presence of the SJ, map to the same cRSSs as 28 ALL patient breakpoints (Papaemmanuil et al., 2014). These data strongly suggests that a plausible mechanism by which at least some single RSS or cRSS breaks are formed, is cut-and-run. Moreover, we find that SJ-mediated breaks occur in many of the genes that frequently acquire somatic mutations in ALL, strongly implying that cut-and-run is involved in the etiology of ALL. Given the longevity of the ESC, it is entirely plausible that the RAG-ESC complex also triggers breaks at other stages of lymphocyte development, leading to chromosome alterations that contribute to other lymphoid cancers.

STAR★METHODS

Detailed methods are provided in the online version of this paper and include the following:

- KEY RESOURCES TABLE
- CONTACT FOR REAGENT AND RESOURCE SHARING
- EXPERIMENTAL MODEL AND SUBJECT DETAILS
  - Mice
  - Cell lines
- METHOD DETAILS
  - Purification of RAG proteins from 293T cells
  - Purification of HMGB1
  - *In vitro* RAG cleavage and binding assays
  - Calculation of ESC matches to consensus RSSs
  - DNase I footprint analysis of RAG-SJ binding
  - Preparation of GA ladder
  - Signal end release assay
  - Generation of 12/23-RSS and SJ lentiviruses
  - *In vivo* RAG cutting assay
  - Calculation of pro-virus copies per cell
  - LM-PCR of extrachromosomal substrates from NIH 3T3 cells
  - LM-PCR of extra-chromosomal substrates from REH cells
  - LM-PCR to detect broken Vλ1-JCλ1 signal ends
  - Quantification of ligated signal ends
  - Detection of γH2AX foci by immunofluorescence
  - Detection of apoptosis
  - LAM-HTGTS assay
- QUANTIFICATION AND STATISTICAL ANALYSIS
- DATA AND SOFTWARE AVAILABILITY

SUPPLEMENTAL INFORMATION

Supplemental Information can be found with this article online at https://doi.org/10.1016/j.molcel.2019.02.025.

ACKNOWLEDGMENTS

We thank Professor Marco Bianchi for HMGB1 expression plasmids. This work was supported by a research grant from Bloodwise (15042), an Engineering and Physical Sciences Research Council studentship (EP/P505593/1 to C.M.K.), a China Scholarship Council studentship (to X.W.), a studentship from the National Centre for the Replacement, Refinement and Reduction of Animals in Research (NC3Rs; NC/K001639/1 to A.L.S.), and a research grant from Yorkshire Cancer Research (L338).

AUTHOR CONTRIBUTIONS

Conceptulization, C.M.K. and J.B.; Investigation, C.M.K., J.N.F.S., X.W., A.P.K., and J.B.; Software, A.L.S., J.N.F.S., and D.R.W.; Formal Analysis, D.R.W., A.L.S., and J.N.F.S.; Resources, A.M.F.; Writing – Original Draft, C.M.K. and J.B.; Writing – Reviewing & Editing, all authors; Funding Acquisition, J.B., P.G.S., R.T., A.M.F., and D.R.W.

DECLARATIONS OF INTERESTS

The authors declare no competing interests.

Received: August 10, 2018
Revised: December 20, 2018
Accepted: February 14, 2019
Published: March 21, 2019

SUPPORTING CITATIONS

The following references appear in the Supplemental Information: Hiom and Gellert (1997); Mizushima and Nagata (1990); Tevelev and Schatz (2000).

REFERENCES

Agrawal, A., and Schatz, D.G. (1997). RAG1 and RAG2 form a stable post-cleavage synaptic complex with DNA containing signal ends in V(D)J recombination. Cell *89*, 43–53.

Arnal, S.M., and Roth, D.B. (2007). Excised V(D)J recombination byproducts threaten genomic integrity. Trends Immunol. *28*, 289–292.

Bergeron, S., Anderson, D.K., and Swanson, P.C. (2006). RAG and HMGB1 Proteins: Purification and Biochemical Analysis of Recombination Signal Complexes. Methods Enzymol. *408*, 511–528.

Bevington, S., and Boyes, J. (2013). Transcription-coupled eviction of histones H2A/H2B governs V(D)J recombination. EMBO J. *32*, 1381–1392.

Bories, J.C., Cayuela, J.M., Loiseau, P., and Sigaux, F. (1991). Expression of human recombination activating genes (RAG1 and RAG2) in neoplastic lymphoid cells: correlation with cell differentiation and antigen receptor expression. Blood *78*, 2053–2061.

Boudinot, P., Drapier, A.M., Cazenave, P.A., and Sanchez, P. (1994). Conserved distribution of lambda subtypes from rearranged gene segments to immunoglobulin synthesis in the mouse B cell repertoire. Eur. J. Immunol. *24*, 2013–2017.

Canela, A., Sridharan, S., Sciascia, N., Tubbs, A., Meltzer, P., Sleckman, B.P., and Nussenzweig, A. (2016). DNA Breaks and End Resection Measured Genome-wide by End Sequencing. Mol. Cell *63*, 898–911.

Coussens, M.A., Wendland, R.L., Deriano, L., Lindsay, C.R., Arnal, S.M., and Roth, D.B. (2013). RAG2's acidic hinge restricts repair-pathway choice and promotes genomic stability. Cell Rep. *4*, 870–878.

Cowell, L.G., Davila, M., Ramsden, D., and Kelsoe, G. (2004). Computational tools for understanding sequence variability in recombination signals. Immunol. Rev. *200*, 57–69.

Cui, X., and Meek, K. (2007). Linking double-stranded DNA breaks to the recombination activating gene complex directs repair to the nonhomologous end-joining pathway. Proc. Natl. Acad. Sci. USA *104*, 17046–17051.

Curry, J.D., Schulz, D., Guidos, C.J., Danska, J.S., Nutter, L., Nussenzweig, A., and Schlissel, M.S. (2007). Chromosomal reinsertion of broken RSS ends during T cell development. J. Exp. Med. *204*, 2293–2303.

Elkin, S.K., Matthews, A.G., and Oettinger, M.A. (2003). The C-terminal portion of RAG2 protects against transposition in vitro. EMBO J. *22*, 1931–1938.

Fugmann, S.D., Villey, I.J., Ptaszek, L.M., and Schatz, D.G. (2000). Identification of two catalytic residues in RAG1 that define a single active site within the RAG1/RAG2 protein complex. Mol. Cell *5*, 97–107.

Gabriel, R., Lombardo, A., Arens, A., Miller, J.C., Genovese, P., Kaeppel, C., Nowrouzi, A., Bartholomae, C.C., Wang, J., Friedman, G., et al. (2011). An unbiased genome-wide analysis of zinc-finger nuclease specificity. Nat. Biotechnol. *29*, 816–823.

Gellert, M. (2002). V(D)J recombination: RAG proteins, repair factors, and regulation. Annu. Rev. Biochem. *71*, 101–132.

Gigi, V., Lewis, S., Shestova, O., Mijušković, M., Deriano, L., Meng, W., Luning Prak, E.T., and Roth, D.B. (2014). RAG2 mutants alter DSB repair pathway choice in vivo and illuminate the nature of 'alternative NHEJ'. Nucleic Acids Res. *42*, 6352–6364.

Grant, C.E., Bailey, T.L., and Noble, W.S. (2011). FIMO: scanning for occurrences of a given motif. Bioinformatics *27*, 1017–1018.

Han, J.O., Steen, S.B., and Roth, D.B. (1999). Intermolecular V(D)J recombination is prohibited specifically at the joining step. Mol. Cell *3*, 331–338.

Helmink, B.A., and Sleckman, B.P. (2012). The response to and repair of RAG-mediated DNA double-strand breaks. Annu. Rev. Immunol. *30*, 175–202.

Hesse, J.E., Lieber, M.R., Mizuuchi, K., and Gellert, M. (1989). V(D)J recombination: a functional definition of the joining signals. Genes Dev. *3*, 1053–1061.

Hillion, S., Youinou, P., and Jamin, C. (2007). Peripheral expression of RAG in human B lymphocytes in normal and pathological conditions is dependent on interleukin-6. Autoimmun. Rev. *6*, 415–420.

Hiom, K., and Gellert, M. (1997). A stable RAG1-RAG2-DNA complex that is active in V(D)J cleavage. Cell *88*, 65–72.

Hu, J., Meyers, R.M., Dong, J., Panchakshari, R.A., Alt, F.W., and Frock, R.L. (2016). Detecting DNA double-stranded breaks in mammalian genomes by linear amplification-mediated high-throughput genome-wide translocation sequencing. Nat. Protoc. *11*, 853–871.

Jäger, U., Böcskör, S., Le, T., Mitterbauer, G., Bolz, I., Chott, A., Kneba, M., Mannhalter, C., and Nadel, B. (2000). Follicular lymphomas' BCL-2/IgH junctions contain templated nucleotide insertions: novel insights into the mechanism of t(14;18) translocation. Blood *95*, 3520–3529.

Kim, D.R., Dai, Y., Mundy, C.L., Yang, W., and Oettinger, M.A. (1999). Mutations of acidic residues in RAG1 define the active site of the V(D)J recombinase. Genes Dev. *13*, 3070–3080.

Kim, M.-S., Chuenchor, W., Chen, X., Cui, Y., Zhang, X., Zhou, Z.H., Gellert, M., and Yang, W. (2018). Cracking the DNA Code for V(D)J Recombination. Mol. Cell *70*, 358–370.e4.

Küppers, R. (2005). Mechanisms of B-cell lymphoma pathogenesis. Nat. Rev. Cancer *5*, 251–262.

Landree, M.A., Wibbenmeyer, J.A., and Roth, D.B. (1999). Mutational analysis of RAG1 and RAG2 identifies three catalytic amino acids in RAG1 critical for both cleavage steps of V(D)J recombination. Genes Dev. *13*, 3059–3069.

Lee, G.S., Neiditch, M.B., Salus, S.S., and Roth, D.B. (2004). RAG proteins shepherd double-strand breaks to a specific pathway, suppressing error-prone repair, but RAG nicking initiates homologous recombination. Cell *117*, 171–184.

Lefranc, M.P., Clement, O., Kaas, Q., Duprat, E., Chastellan, P., Coelho, I., Combres, K., Ginestoux, C., Giudicelli, V., Chaume, D., and Lefranc, G. (2005). IMGT-Choreography for immunogenetics and immunoinformatics. In Silico Biol. (Gedrukt) *5*, 45–60.

Lescale, C., Abramowski, V., Bedora-Faure, M., Murigneux, V., Vera, G., Roth, D.B., Revy, P., de Villartay, J.-P., and Deriano, L. (2016). RAG2 and XLF/Cernunnos interplay reveals a novel role for the RAG complex in DNA repair. Nat. Commun. *7*, 10529.

Livak, F., and Schatz, D.G. (1996). T-cell receptor alpha locus V(D)J recombination by-products are abundant in thymocytes and mature T cells. Mol. Cell. Biol. *16*, 609–618.

Liyanage, M., Weaver, Z., Barlow, C., Coleman, A., Pankratz, D.G., Anderson, S., Wynshaw-Boris, A., and Ried, T. (2000). Abnormal rearrangement within the alpha/delta T-cell receptor locus in lymphomas from Atm-deficient mice. Blood *96*, 1940–1946.

Lovely, G.A., Brewster, R.C., Schatz, D.G., Baltimore, D., and Phillips, R. (2015). Single-molecule analysis of RAG-mediated V(D)J DNA cleavage. Proc. Natl. Acad. Sci. USA *112*, E1715–E1723.

Marculescu, R., Le, T., Simon, P., Jaeger, U., and Nadel, B. (2002). V(D)J-mediated translocations in lymphoid neoplasms: a functional assessment of genomic instability by cryptic sites. J. Exp. Med. *195*, 85–98.

Mizushima, S., and Nagata, S. (1990). pEF-BOS, a powerful mammalian expression vector. Nucleic Acids Res. *18*, 5322.

Nagawa, F., Kodama, M., Nishihara, T., Ishiguro, K., and Sakano, H. (2002). Footprint analysis of recombination signal sequences in the 12/23 synaptic complex of V(D)J recombination. Mol. Cell. Biol. *22*, 7217–7225.

Nagawa, F., Hirose, S., Nishizumi, H., Nishihara, T., and Sakano, H. (2004). Joining mutants of RAG1 and RAG2 that demonstrate impaired interactions with the coding-end DNA. J. Biol. Chem. *279*, 38360–38368.

Neiditch, M.B., Lee, G.S., Huye, L.E., Brandt, V.L., and Roth, D.B. (2002). The V(D)J recombinase efficiently cleaves and transposes signal joints. Mol. Cell *9*, 871–878.

Papaemmanuil, E., Rapado, I., Li, Y., Potter, N.E., Wedge, D.C., Tubio, J., Alexandrov, L.B., Van Loo, P., Cooke, S.L., Marshall, J., et al. (2014). RAG-mediated recombination is the predominant driver of oncogenic rearrangement in ETV6-RUNX1 acute lymphoblastic leukemia. Nat. Genet. *46*, 116–125.

Raghavan, S.C., Kirsch, I.R., and Lieber, M.R. (2001). Analysis of the V(D)J recombination efficiency at lymphoid chromosomal translocation breakpoints. J. Biol. Chem. *276*, 29126–29133.

Ramsden, D.A., and Gellert, M. (1995). Formation and resolution of double-strand break intermediates in V(D)J rearrangement. Genes Dev. *9*, 2409–2420.

Ramsden, D.A., Baetz, K., and Wu, G.E. (1994). Conservation of sequence in recombination signal sequence spacers. Nucleic Acids Res. *22*, 1785–1796.

Rogakou, E.P., Pilch, D.R., Orr, A.H., Ivanova, V.S., and Bonner, W.M. (1998). DNA double-stranded breaks induce histone H2AX phosphorylation on serine 139. J. Biol. Chem. *273*, 5858–5868.

Rommel, P.C., Oliveira, T.Y., Nussenzweig, M.C., and Robbiani, D.F. (2017). RAG1/2 induces genomic insertions by mobilizing DNA into RAG1/2-independent breaks. J. Exp. Med. *214*, 814–831.

Rosenfeld, C., Goutner, A., Venuat, A.M., Choquet, C., Pico, J.L., Dore, J.F., Liabeuf, A., Durandy, A., Desgrange, C., and De The, G. (1977). An effect human leukaemic cell line: Reh. Eur. J. Cancer *13*, 377–379.

Roth, D.B. (2003). Restraining the V(D)J recombinase. Nat. Rev. Immunol. *3*, 656–666.

Roth, D.B., Zhu, C., and Gellert, M. (1993). Characterization of broken DNA molecules associated with V(D)J recombination. Proc. Natl. Acad. Sci. USA *90*, 10788–10792.

Ru, H., Chambers, M.G., Fu, T.-M., Tong, A.B., Liao, M., and Wu, H. (2015). Molecular Mechanism of V(D)J Recombination from Synaptic RAG1-RAG2 Complex Structures. Cell *163*, 1138–1152.

Ru, H., Mi, W., Zhang, P., Alt, F.W., Schatz, D.G., Liao, M., and Wu, H. (2018). DNA melting initiates the RAG catalytic pathway. Nat. Struct. Mol. Biol. *25*, 732–742.

Sadofsky, M.J., Hesse, J.E., McBlane, J.F., and Gellert, M. (1993). Expression and V(D)J recombination activity of mutated RAG-1 proteins. Nucleic Acids Res. *21*, 5644–5650.

Sadofsky, M.J., Hesse, J.E., van Gent, D.C., and Gellert, M. (1995). RAG-1 mutations that affect the target specificity of V(D)j recombination: a possible direct role of RAG-1 in site recognition. Genes Dev. *9*, 2193–2199.

Schatz, D.G., and Swanson, P.C. (2011). V(D)J recombination: mechanisms of initiation. Annu. Rev. Genet. *45*, 167–202.

Steen, S.B., Gomelsky, L., and Roth, D.B. (1996). The 12/23 rule is enforced at the cleavage step of V(D)J recombination in vivo. Genes Cells *1*, 543–553.

Swanson, P.C. (2002). A RAG-1/RAG-2 tetramer supports 12/23-regulated synapsis, cleavage, and transposition of V(D)J recombination signals. Mol. Cell. Biol. *22*, 7790–7801.

Tevelev, A., and Schatz, D.G. (2000). Intermolecular V(D)J recombination. J. Biol. Chem. *275*, 8341–8348.

Tsuzuki, S., Seto, M., Greaves, M., and Enver, T. (2004). Modeling first-hit functions of the t(12;21) TEL-AML1 translocation in mice. Proc. Natl. Acad. Sci. USA *101*, 8443–8448.

van Gent, D.C., Hiom, K., Paull, T.T., and Gellert, M. (1997). Stimulation of V(D)J cleavage by high mobility group proteins. EMBO J. *16*, 2665–2670.

Vanura, K., Montpellier, B., Le, T., Spicuglia, S., Navarro, J.M., Cabaud, O., Roulland, S., Vachez, E., Prinz, I., Ferrier, P., et al. (2007). In vivo reinsertion of excised episomes by the V(D)J recombinase: a potential threat to genomic stability. PLoS Biol. *5*, e43.

Welzel, N., Le, T., Marculescu, R., Mitterbauer, G., Chott, A., Pott, C., Kneba, M., Du, M.Q., Kusec, R., Drach, J., et al. (2001). Templated nucleotide addition and immunoglobulin JH-gene utilization in t(11;14) junctions: implications for the mechanism of translocation and the origin of mantle cell lymphoma. Cancer Res. *61*, 1629–1636.

Yin, F.F., Bailey, S., Innis, C.A., Ciubotaru, M., Kamtekar, S., Steitz, T.A., and Schatz, D.G. (2009). Structure of the RAG1 nonamer binding domain with DNA reveals a dimer that mediates DNA synapsis. Nat. Struct. Mol. Biol. *16*, 499–508.

STAR★METHODS

KEY RESOURCES TABLE

REAGENT or RESOURCE	SOURCE	IDENTIFIER
Antibodies		
Anti-phospho-histone H2A.X (Ser139)	Cell Signaling Technology	Cat#2577;RRID:AB_2118010
AlexaFluor488 anti-rabbit IgG (H+L) F(ab')$_2$ Fragment	Cell Signaling Technologies	Cat#4412;RRID:AB_1904025
AlexaFluor568 goat anti-rabbit IgG	Life Technologies	Cat#A11011;RRID:AB_143157
APC-conjugated Annexin V	Thermo Fisher Scientific	Cat#17-8007-72; RRID:AB_2575165
Bacterial and Virus Strains		
DH5α competent cells	Thermo Fisher Scientific	Cat#18265017
BL21(DE3) competent cells	NEB	Cat#C25271
Endura competent cells	Lucigen	Cat#60240
Chemicals, Peptides, and Recombinant Proteins		
Protease inhibitors	Roche	Cat#11697498001
Amylose resin	NEB	Cat#E80225
Ni-NTA agarose resin	QIAGEN	Cat#30230
HiTrap Q XL column	GE Healthcare	Cat#17-5158-01
Proteinase K	Roche	Cat#03115879001
NheI	NEB	Cat#R3131
ApaI	NEB	Cat#R0114
EcoRV	NEB	Cat#R0195
ApaLI	NEB	Cat#R0507
Klenow DNA polymerase	NEB	Cat#M0212
T4 DNA ligase	NEB	Cat#M0202
Vent (exo$^-$) DNA polymerase	NEB	Cat#M0254
Taq DNA polymerase	NEB	Cat#M0267
DNase I	Worthington	Cat#DPRF
α-^{32}P dCTP	Perkin Elmer	Cat#NEG013H
γ-^{32}P ATP	Perkin Elmer	Cat#NEG002A
Poly-L-lysine	Sigma	Cat#P4704
Vectashield plus DAPI	Vector laboratories	Cat#H-1200
Polyethylenimine	Sigma	Cat#408727
Polybrene	Merck	Cat#TR-1003-G
dsGreen Gel staining solution	Lumiprobe	Cat#A10010
Critical Commercial Assays		
Annexin V Apoptosis detection kit - APC	Thermo Fisher Scientific	Cat#88-8007-72
SensiFAST SYBR No-Rox	Bioline	Cat#BIO-72005
QuantiFluor dsDNA System	Promega	Cat#E2671
MiSeq Reagent kit v2 (500 cycle)	Illumina	Cat#MS-102-2003
PhiX Control v3 kit	Illumina	Cat#FC-110-3001
Deposited Data		
FASTQ raw sequence files from LAM-HTGTS	This paper	Uploaded to NCBI SRA; PRJNA483469
Experimental Models: Cell Lines		
REH - human ALL cell line carrying the *ETV6/RUNX1* (*TEL-AML*) fusion gene	Greaves lab, Institute of Cancer Research, London; Rosenfeld et al., 1977	N/A
NIH 3T3 - mouse embryo fibroblast cell line	Bonifer lab, University of Birmingham	N/A

(*Continued on next page*)

Continued

REAGENT or RESOURCE	SOURCE	IDENTIFIER
Cos 7 - African green monkey kidney fibroblast cell line	Harris lab, University of Leeds	N/A
HEK293T - human embryonic kidney cell line	Harris lab, University of Leeds	N/A
Experimental Models: Organisms/Strains		
CBA/C57BL/6J mice	University of Leeds Facility	N/A
λ5-IRF4 transgenic mice	Bevington and Boyes, 2013	PIP3 mice
Oligonucleotides		
Primers used in RAG cutting assays are given in Table S4	This paper; Table S4	N/A
Primers used in PCR assays are given in Table S5	This paper; Table S5	N/A
Adaptors and common primers for HTGTS	Hu et al., 2016	N/A
Recombinant DNA		
Plasmid: pETM11-HMGB1	A gift from Professor Marco Bianchi	N/A
Plasmid: pEF-McR1	This paper; Table S6	N/A
Plasmid: pEF-McR2	This paper; Table S6	N/A
Plasmid: pJH290	Steen et al., 1996; Han et al., 1999	N/A
Plasmid: pJH29012SJ	This paper; Table S6	N/A
Plasmid: pJH29023SJ	This paper; Table S6	N/A
Plasmid: pJδ1+	This paper; Table S6	N/A
Plasmid: pSJ+	This paper; Table S6	N/A
Plasmid: p23+	This paper; Table S6	N/A
Plasmid: p12+	This paper; Table S6	N/A
Plasmid: pSJ12d7+	This paper; Table S6	N/A
Plasmid: pSJ23d7+	This paper; Table S6	N/A
Plasmid: pSJ12d9+	This paper; Table S6	N/A
Plasmid: pSJ23d9+	This paper; Table S6	N/A
Plasmid: pSJ12d9CAC+	This paper; Table S6	N/A
Plasmid: pSJ23d9CAC+	This paper; Table S6	N/A
Plasmid: pSJ12d923d9CAC+	This paper; Table S6	N/A
Plasmid: pJH548	Sadofsky et al., 1993	N/A
Plasmid: pEFflRAG2	This paper; Table S6	N/A
Plasmid: pEFcRAG2	This paper; Table S6	N/A
Plasmid: pWPI	Addgene	Cat#12254
Plasmid: pWPI-12RSS	This paper; Table S6	N/A
Plasmid: pWPI-23RSS	This paper; Table S6	N/A
Plasmid: pWPI-SJ	This paper; Table S6	N/A
Plasmid: pWPI-mutant SJ	This paper; Table S6	N/A
Plasmid: LentiCRISPR-v2	Addgene	Cat#52961
Plasmid: pCMVR8.74	Addgene	Cat#22036
Plasmid: pMD2.G	Addgene	Cat#12259
Software and Algorithms		
HTGTS pipeline	Hu et al., 2016	http://robinmeyers.github.io/transloc_pipeline/
GraphPad Prism	N/A	https://www.graphpad.com/
IMGT® repertoire (The international ImMunoGeneTics information system)	Lefranc et al., 2005	http://www.imgt.org
Recombination Signal Sequences Site	Cowell et al., 2004	https://www.itb.cnr.it/rss/
FIMO	Grant et al., 2011	http://meme-suite.org/
COSMIC	COSMIC mutation data (genomic screens) Release v85	https://cancer.sanger.ac.uk/cosmic

(*Continued on next page*)

Continued

REAGENT or RESOURCE	SOURCE	IDENTIFIER
The R Project for Statistical Computing	N/A	https://www.r-project.org/
Analysis of translocations to RSSs	This paper	Deposited on GitHub: https://github.com/boyeslab/cutandrun/
Analysis of chromosome distribution of translocations	This paper	Deposited on GitHub: https://github.com/boyeslab/cutandrun/
Analysis of overlaps between target genes and translocations	This paper	Deposited on GitHub: https://github.com/boyeslab/cutandrun/
Analysis of the intersection between ALL patient breakpoints and translocations	This paper	Deposited on GitHub: https://github.com/boyeslab/cutandrun/

CONTACT FOR REAGENT AND RESOURCE SHARING

Further information and requests for resources and reagents should be directed to and will be fulfilled by the corresponding author, Joan Boyes (j.m.boyes@leeds.ac.uk).

EXPERIMENTAL MODEL AND SUBJECT DETAILS

Mice

PIP3 mice have been described previously (Bevington and Boyes, 2013) and express the IRF4 cDNA under the control of the pro-B cell specific lambda promoter and LCR. Non-transgenic CBA/C57BL/6J were obtained from the University of Leeds animal facility. Animals were sacrificed at 5-7 weeks, bone marrow was removed from femurs and used for the isolation of pro- or pre-B cells by flow cytometry. A single mouse was used to isolate DNA from pro-B or pre-B cells per independent experiment; equivalent numbers of male and female animals were used overall. All animal procedures were performed under Home Office license PPL 70/7697, following review by the University of Leeds ethics committee. They were housed in a full barrier facility, with no more than six animals per cage, where all mice are free of common pathogens, including murine norovirus, *Pasteurella* and *Helicobacter*.

Cell lines

REH cells were established from the peripheral blood of a 15-year old female with acute lymphoblastic leukemia (Rosenfeld et al., 1977). This cell line carries t(12:21) that generates the *ETV6/RUNX1* (*TEL-AML*) fusion gene. Cells were maintained in RPMI1640 with 10% fetal calf serum at 37°C in 5% CO_2 and were grown at a density between 5×10^5 and 2×10^6 (https://www.dsmz.de/catalogues/details/culture/ACC-22.html).

NIH 3T3 (male mouse embryo fibroblast) cells, Cos 7 (male African green monkey kidney fibroblast) cells and HEK293T (female human embryonic kidney) cells were maintained in DMEM with 10% fetal calf serum, 3.8 mM L-glutamine, 50 U/ml penicillin and 0.05 mg/ml streptomycin at 37°C in 5% CO_2. These adherent cell lines were passaged every 2-3 days to ensure that they remained sub-confluent. They have not been authenticated.

METHOD DETAILS

Purification of RAG proteins from 293T cells

MBP-tagged core RAG proteins were purified from HEK293T cells according to Bergeron et al. (2006). HEK293T cells were seeded at 1×10^6 cells per 10 cm plate. The following day, the medium was changed three hours prior to transfection; the cells were then transfected with 5 µg each of the plasmids pEF-McR1 and pEF-McR2 (Table S6) using PEI. Cells were harvested after 48 hours, washed twice in PBS and frozen until use. All purification steps were performed at 4°C. Briefly, cell pellets (typically from 30 dishes) were thawed on ice and resuspended in a total of 10 mL of lysis buffer (10 mM NaP_i [pH 7.2], 0.5 M NaCl, 1 mM DTT, 0.25% TWEEN-20, 1x protease inhibitors with EDTA (Roche), 1 mM PMSF, 1 mM benzamidine). Cells were lysed by using a Dounce homogenizer for 20 strokes with a tight-fitting pestle, then centrifuged for 40 minutes at 11,000 x *g* (30,000 rpm), 4°C, in a SW55 Ti rotor (Beckman). The supernatant was loaded onto a 1 mL amylose column equilibrated with buffer A (10 mM NaP_i [pH 7.2], 0.5 M NaCl, 1 mM DTT, 0.25% TWEEN-20). The column was washed with 10 column volumes (CV) of buffer A, followed by 10 CV of buffer B (buffer A without TWEEN-20) and protein was eluted with 10 CV buffer C (Buffer A without TWEEN-20, with 10 mM maltose). Fractions were analyzed by SDS-PAGE and RAG-containing fractions were pooled and dialysed twice for three hours against dialysis buffer (25 mM Tris-HCl [pH 8.0], 150 mM KCl, 2 mM DTT, 10% glycerol). Dialysed protein was aliquoted, snap-frozen in a dry ice/ethanol bath and stored at −80°C.

Purification of HMGB1

The rat HMGB1 expression plasmid pETM11-HMGB1 was a kind gift from Prof Marco Bianchi (San Raffaele Institute, Milan, Italy). This plasmid contains the coding sequence of full-length rat HMGB1 with an N-terminal 6x His tag. BL21(DE3) cells were grown to an OD_{600} of 0.6 - 0.8, at which point expression of HMGB1 was induced with 0.5 mM IPTG. Induced cells were incubated at 25°C with shaking (250 rpm) for 16 hours. Cells were harvested for 20 min at 4,000 x *g*, washed once in ice-cold PBS, and the pellet was snap-frozen in a dry ice/ethanol bath and stored at −80°C until purification. The pellet was thawed on ice and resuspended in 20 mL lysis buffer (20 mM Tris-HCl [pH 8.0], 150 mM NaCl, 10 mM imidazole, 2 mM β-mercaptoethanol, 0.2% Igepal CA-630, 1x EDTA-free protease inhibitor cocktail (Roche), 1 mM benzamidine, 1 mM PMSF). All subsequent steps were carried out at 4°C. The resuspended cells were sonicated for four cycles of 2 minutes at 10 microns, 2 minutes on ice, and the lysate was clarified by centrifugation at 16,000 x *g* for 30 minutes. The cleared lysate was then filtered through a 0.2 μm syringe filter unit and bound to 5 mL Ni-NTA resin on a rotating shaker for 30 minutes. The flowthrough was collected and applied to an additional 2.5 mL of Ni-NTA resin for 30 minutes to maximize recovery of His-tagged HMGB1. Both columns were then washed with 10 CVs of lysis buffer, 5 CVs of lysis buffer without Igepal CA-630 or protease inhibitors, 5 CVs of wash buffer 1 (20 mM Tris-HCl [pH 8.0], 1 M NaCl, 10 mM imidazole, 2 mM β-mercaptoethanol) and 5 CVs of wash buffer 2 (20 mM Tris-HCl [pH 8.0], 150 mM NaCl, 30 mM imidazole, 2 mM β-mercaptoethanol). HMGB1 was eluted with elution buffer (Tris-HCl [pH 8.0], 150 mM NaCl, 300 mM imidazole, 2 mM β-mercaptoethanol).

The 6x His tag was removed by adding Tobacco Etch Virus (TEV) protease at a ratio of 1:50 TEV:HMGB1 and dialysing the proteins overnight against dialysis buffer (20 mM Tris-HCl [pH 8.0], 2 mM β-mercaptoethanol and 20 mM NaCl) at 4°C. Completion of TEV protease digestion was determined by gel electrophoresis. The dialysate was spun at 4,000 x *g* for 30 minutes and applied to 3 mL Ni-NTA agarose, pre-equilibrated in dialysis buffer, by rotation for 30 minutes at 4°C. Unbound (cleaved HMGB1) was recovered by centrifugation at 4,000 x *g* for 5 minutes.

Cleaved HMGB1 was loaded onto a 1 mL HiTrap Q XL column (GE Healthcare) equilibrated with buffer A (20 mM Tris-HCl [pH 8.0], 1 mM DTT, 80 mM NaCl). The column was washed with 9 CV of buffer A at 80 mM NaCl and HMGB1 was eluted with a gradient of 80-650 mM NaCl (20 mM Tris-HCl [pH 8.0], 1 mM DTT) over 25 CVs. HMGB1-containing fractions were dialysed against 100 volumes of storage buffer (25 mM Tris-HCl [pH 8.0], 150 mM KCl, 2 mM DTT, 10% glycerol).

In vitro RAG cleavage and binding assays

RAG cleavage reactions contained 25 mM MOPS pH 7.0, 50 mM potassium acetate, 1 mM DTT, 100 μg/ml BSA, 1 mM $MgCl_2$, 2 nM radiolabelled oligonucleotide substrate, 100 ng (400 nM) HMGB1 and 2 μl of purified RAG proteins (~80 nM) in a total volume of 10 μl. Where indicated, an unlabelled partner oligonucleotide (20 nM) was included. Reactions were incubated at 37°C for 15 minutes to ensure that differences in activity are measured under conditions where all reagents are in excess. HMGB1 was included to increase the affinity of RAGs for RSSs (van Gent et al., 1997); in the presence of HMGB1, the Kd of RAGs for a 12-RSS is 4.1 ± 1.6 nM and 8.8 ± 4.0 nM for a 23-RSS (Lovely et al., 2015). Stop buffer was added to give final concentrations: 50 mM Tris-HCl pH 8.0, 0.1% SDS, 5 mM EDTA, 0.175 mg/ml proteinase K, 1x DNA loading buffer, followed by incubation at 37°C for 30 minutes. The reaction was loaded onto a 12% TAE polyacrylamide gel and separated for 1.5 hours at 170 V. Gels were soaked in 70% methanol for 20 minutes and dried onto Whatman® 3MM paper before being visualized using an FLA-5100 phosphorimager (Fujifilm).

To analyze RAG nicking and hairpinning activity, cleavage assays were performed as above but following cutting, the reaction was purified by phenol-chloroform extraction/ethanol precipitation. This was resuspended in 5 μl formamide loading buffer and run on a 12% (19:1) polyacrylamide gel containing 7 M urea for 1 hour at 500 V.

RAG cleavage on long DNA templates was performed as above but where the SJ and RSS were present in *cis*. The vector pJH290, that has a 12- and 23-RSS in a convergent orientation, was modified to generate pJH29012SJ and pJH29023SJ (Table S6) that contain an SJ in place of each of the RSSs. Fragments of 695-741 bp that carry two RSSs or SJ+RSS were amplified from these plasmids via PCR with primers JH290F and JH290R (Table S5), gel extracted and used in RAG cutting assays. Following cutting, products were separated on a 5.5% TAE polyacrylamide gel for 2 hours at 170 V. Gels were stained with dsGreen and visualized using an FLA-5100 phosphorimager (Fujifilm).

RAG binding assays were identical to cleavage reactions, except that the divalent cation was Ca^{2+} instead of Mg^{2+}. After incubation, reactions were chilled on ice for 5 minutes and loaded onto a 4% (19:1) polyacrylamide gel in 0.5 x TBE, pre-chilled to 4°C. The gels were run at 170 V for 2 hours at 4°C, dried and exposed to a phosphorimager screen as above. Oligonucleotide sequences are given in Table S4.

Calculation of ESC matches to consensus RSSs

The percentage of ESCs where both RSSs display a high similarity to the consensus RSS sequence was determined by generating all possible head-to-head RSS combinations for each recombining gene segment in each human antigen receptor locus. All RSS sequences were retrieved from the IMGT® repertoire (The international ImMunoGeneTics information system, accessible at http://www.imgt.org) (Lefranc et al., 2005), and similarity to consensus RSSs was determined for each possible ESC based on the following criteria: either containing two consensus heptamers (CACAGTG) or containing heptamers with CAC and allowing a single mismatch, with both criteria requiring positions 5 and 6 (AA) to be conserved in both nonamers. The percentage of ESCs matching these criteria for each locus was calculated and these percentages were weighted according to gene segment usage frequency, retrieved from the IMGT®/GeneFrequency database (Lefranc et al., 2005).

DNase I footprint analysis of RAG-SJ binding

The wild-type and mutated SJ footprint probes were prepared by digesting 100 μg pSJ+ or the plasmids carrying the respective SJ mutations: pSJ12d9CAC+, pSJ23d9CAC+ and pSJ12d923d9CAC+ (Table S6) with NheI and ApaI. The SJ-containing fragments (258 bp) were gel-purified, resuspended in dH_2O, and radiolabelled by filling in the recessed NheI end with α-^{32}P-dCTP and Klenow DNA polymerase. Labeling reactions, containing 5 pmol of the ApaI/NheI fragment, 5 μl (50 μCi) α-^{32}P-dCTP, 1x NEBuffer 2, and 10 units of Klenow DNA polymerase, were incubated for 30 min at room temperature (RT), then supplemented with 5 mM dNTP mix for 10 minutes to complete the fill-in reaction. The labeled fragment was then purified by phenol-chloroform extraction/ethanol precipitation and resuspended in 50 μl TE.

Binding reactions containing 1x buffer (25 mM MOPS [pH 7.0], 0.1 mg/ml BSA, 50 mM KOAc, 2 mM DTT, 1 mM $CaCl_2$), SJ probe (~150 - 250 counts per second), 20% DMSO, 0.2 μg polydI.dC, and 500 nM cRAG proteins, were incubated at 37°C for 10 minutes. $MgCl_2$, at 5 mM final concentration, and 2 units of DNase I were added and the mixture was incubated at RT for 1 minute. DNase I reactions were quenched by the addition of EDTA to 15 mM, and then purified by phenol-chloroform/ethanol precipitation. DNA was resuspended in 3 μl formamide loading buffer, boiled for 3 min and run alongside the appropriate GA marker on a pre-warmed 6% (19:1) denaturing polyacrylamide gel for 45 min at 38 mA. Gels were transferred to Whatman® 3MM paper and dried in a BioRad vacuum drier for 45 min with an 80°C heated lid. The dried gel was exposed to a PhosphorImager screen and scanned using an FLA-5100 PhosphorImager (FujiFilm).

Preparation of GA ladder

The GA ladder was prepared by ethanol precipitating a fifth of the labeled probe stock and resuspending it in 10 μl dH_2O. 25 μl 100% formic acid was added, incubated for 2 minutes at RT, then quenched by addition of 200 μl stop buffer (300 mM NaOAc [pH 5.2], 0.1 mM EDTA [pH 8.0], 0.1 mg/ml yeast tRNA) and 150 μl 100% ethanol, and chilled to −20°C. DNA was precipitated by centrifugation at 16,000 x *g* for 10 min and washed twice with 70% ethanol. The pellet was resuspended in 200 μl dH_2O and precipitated once more, washed with 70% ethanol, and dried in a SpeedVac. The dried pellet was resuspended in 70 μl 10% (v/v) piperidine, transferred to a screw-cap tube and incubated in a 90°C heat block for 30 min. Piperidine was evaporated in a SpeedVac, and the pellet was resuspended in 30 μl of dH_2O. The DNA was then transferred to a fresh tube to minimize piperidine contamination, and dried again in a SpeedVac. This was repeated twice more, resuspending in 20 μl dH_2O the first time, and 10 μl formamide loading buffer the second time.

Signal end release assay

This was performed as described by Coussens et al. (2013). Specifically, reactions were as for *in vitro* RAGs cutting assays, described above, except the oligonucleotides were labeled on the anti-sense strand to detect signal ends. Following cutting for 15 minutes at 37°C, the reactions were challenged at different temperatures ranging from 37°C to 60°C for 30 minutes. To detect signal end release, half of the reaction was resolved on a 8% (19:1) polyacrylamide gel in 0.5 x TBE at 170 V for 2 hours at 4°C, dried and exposed to a phosphorimager screen as above. To detect RAG cutting, the remainder was treated with stop buffer as above, resolved on a 12% 1 x TAE polyacrylamide gel and separated for 1.5 hours at 170 V. These latter gels were soaked in 70% methanol for 20 minutes and dried onto Whatman® 3MM paper before being visualized using an FLA-5100 phosphorimager (Fujifilm). The amount of signal end release was normalized to the level of RAG cutting.

Generation of 12/23-RSS and SJ lentiviruses

Lentiviruses carrying a 12- or 23-RSS or the SJ were generated by transfection of the relevant plasmids (Table S6) into 293T cells. Specifically, 293T cells were seeded at a density of 2.5×10^6 cells/plate in five 10 cm^2 dishes and the following day, the medium was changed to antibiotic-free medium. Three hours later, cells were transfected with 30 μg PEI and 4.9 μg of lentiviral vector (pWPI-12RSS, pWPI-23RSS or pWPI-SJ, Table S6), 2.6 μg of LV packaging vector pCMVR8.74, a gift from Didier Trono (Addgene plasmid #22036), modified to express a mutated integrase gene (D64V) and 2.5 μg of the vector that expresses the coat protein, pMD2.G, a gift from Didier Trono (Addgene plasmid #12259). After 48 hours, the growth medium containing the virus was harvested by centrifugation at 300 x *g* for 3 minutes to remove cell debris. The supernatant was added to one quarter volume of sucrose buffer (50 mM Tris-HCl [pH 7.4], 100 mM NaCl, 0.5 mM EDTA, 10% sucrose), followed by centrifugation at 14,000 x *g* for 4 hours. The supernatant was discarded and the pellet was resuspended in PBS overnight at 4°C. Aliquots of the concentrated virus were stored at −80°C until use.

In vivo RAG cutting assay

REH cells were seeded at 1×10^6 per well in 12-well plates and were transduced with integrase-deficient lentiviruses. These generate episomes by reverse transcription of the lentiviruses to produce linear double stranded DNA which is then converted to circular DNA either via homologous recombination between the LTRs or by NHEJ-mediated joining. Lentiviruses that encode EGFP and carry either a 12- or 23-RSS or a SJ were used. After incubation for 48 hours, the cells were mixed well and 20% were used to check the infection efficiency (which was routinely 45%–55%) by analyzing EGFP expression via flow cytometry using a LSRFortessa (Becton Dickinson). DNA was extracted from the remaining cells by Hirt extraction as described below.

NIH 3T3 cells were plated at 1 × 10^5 cells/well of a 6-well dish in 3 mL complete DMEM medium the day before transfection. They were transfected with 0.5 μg of RSS-containing plasmid and/or a SJ-containing plasmid, and 1 μg each of RAG1 (Sadofsky et al., 1993) and RAG2 expression vectors using PEI. Plasmids are described in Table S6. Cells were harvested 48 hours after transfection and plasmid DNA was recovered via Hirt extraction.

Briefly, transfected cells were washed twice with 3 mL of PBS, and lysed directly on the plate by addition of 0.8 mL of buffer 1 (0.6% (w/v) SDS, 10 mM Tris [pH 8.0], 1 mM EDTA [pH 8.0]), and incubation at room temperature for 10 minutes. Subsequently, 0.2 mL of buffer 2 (5 M NaCl, 10 mM Tris [pH 8.0], 1 mM EDTA [pH 8.0]) was added, mixed by swirling and left for 2 minutes, after which the cells were scraped into a 1.5 mL tube and stored at 4°C overnight. SDS and protein was removed by centrifugation at 16,000 x *g*, 4°C, for 40 minutes. The supernatant was transferred to a fresh tube, followed by two rounds of phenol-chloroform extraction. 1 mL 100% butanol was then added, mixed vigorously, and spun at top-speed for 1 minute. The butanol layer was removed and DNA was precipitated in 100% ethanol and 2.5 M NH_4OAc, then washed in 1 mL 70% ethanol and resuspended in 30 μl dH_2O. 1 ng of each sample was analyzed by qPCR.

Quantitative PCRs contained 5 μl 2x SensiFAST SYBR® No-ROX mix (Bioline), 400 nM of forward and reverse primers and 1 ng of sample in a total volume of 10 μl. PCRs were carried out in a Corbett RotorGene 6000 qPCR machine, and analyzed using the Corbett Rotor-Gene 6000 Series Software (v.1.7, build 87).

The amount of intact 12-RSSs in p12+ and pWPI-12RSS were quantified using the primer pair 12-RSS Cut Fwd and 12-RSS Cut Rev. The amount of intact 23-RSSs in p23+ and pWPI-23RSS were quantified using 23-RSS Cut Fwd and 23-RSS Cut Rev. The total amount of 12-RSS (p12+) or 23-RSS plasmid (p23+) was quantified using primers Neo3 Fwd and Neo3 Rev while the levels of the proviruses were quantified using 12-RSS virus Norm Fwd/virus Norm Reverse and 23-RSS virus Norm Fwd and 23-RSS Norm Rev, respectively. The amount of intact SJ and mutant SJ from both plasmids and proviruses was quantified using the primer pair SJ Cut Fwd and SJ Cut Rev. The total amount of SJ plasmids was quantified using primers Jd Norm Fwd and Jd Norm Rev and SJ-carrying proviruses with SJ virus Norm Fwd and virus Norm Rev, except SJ12d7 and SJ23d9 where 23-RSS virus Norm Fwd was substituted for virus Norm Rev. Primer sequences are given in Table S5. Following an initial denaturation at 95°C for 6 minutes, thermal cycling conditions were 95°C for 10 s; Tm for 15 s; 72°C for 10 s for 40 cycles.

Calculation of pro-virus copies per cell

Genomic DNA was extracted from transduced or non-transduced REH cells and a 680 bp fragment was amplified from the genome using the primer pair Genomic DNA PCR 680bp Fwd and Genomic DNA PCR 680bp Rev (Table S5). This PCR product was gel extracted and following four phenol/chloroform extractions and ethanol precipitation, it was resuspended in 50 μL of TE. The concentration was determined using the QuantiFluor dsDNA System (Promega) and the copies of the genomic fragment per ml were calculated. The concentrations of pWPI-12RSS, pWPI-23RSS and pWPI-SJ plasmid DNAs were similarly measured. The genomic fragment was diluted to 1 × 10^{10} copies/ml and a serial dilution from 1 × 10^8 to 1 × 10^2 copies/ml was generated and used as a standard curve for qPCR. A similar standard curve was generated for each of the pWPI plasmids. The copies of genomic DNA in the test samples was determined using the primer pair Genomic DNA qPCR 200bp Fwd and Genomic DNA qPCR 200bp Rev. The copies of SJ, 12-RSS and 23-RSS proviruses were determined using the primer pairs, SJ virus norm Fwd and virus norm Rev, 12RSS virus norm Fwd and virus norm Rev and 23RSS virus norm Fwd and 23RSS virus norm Rev, respectively (Table S5). The copies of 12-RSS, 23-RSS or SJ-containing proviruses per cell were calculated by dividing the copies of the 12-RSS, 23-RSS or SJ by the amount of copies of the genomic sample and then multiplying by 2 (chromosome 14, from which the 680 bp fragment was amplified, is diploid in REH cells; https://www.dsmz.de/catalogues/details/culture/ACC-22.html).

LM-PCR of extrachromosomal substrates from NIH 3T3 cells

Plasmid DNA extracted from transfected NIH 3T3 cells was used in an ligation-mediated PCR (LM-PCR) assay to determine whether double-strand breaks are present at the plasmid RSSs. Only double-strand breaks, which are generated by RAG cleavage at the signal ends, will ligate with a linker oligonucleotide and give an LM-PCR product. 5 ng of extracted 12-RSS plasmid DNA, digested with EcoRV was used as positive control and processed in the same way as recovered Hirt samples while the SJ plasmid was digested with ApaLI and filled in by Klenow DNA polymerase. The FM25 and FM11 oligonucleotides (Table S5) were annealed and used in a ligation reaction containing 5 ng of Hirt extracted plasmid DNA, 3 μl 10x T4 DNA ligase buffer and 5 units T4 DNA ligase in a final volume of 30 μl. Ligation reactions were incubated at 16°C for 16 hours, then purified by phenol-chloroform extraction/ethanol precipitation and resuspended in 10 μl dH_2O. In the first round of PCR, 2 μl resuspended DNA was used in a reaction containing 3 μl 10x ThermolPol buffer, 1 μl Vent polymerase (NEB), 0.6 μM each of CMVfwd (12-RSS) or BGHRev (SJ) and FM25 primers (Table S5), and 300 nM each dNTP in a total of 30 μl. Following an initial denaturation at 95°C for 5 minutes, cycling conditions were 95°C for 30 s; 57°C for 20 s; 72°C for 30 s for 25 cycles.

In the second round of PCR, 1 μl first round PCR product was used in a reaction identical to the first round of PCR, except the 12-RSS fwd or SJRev and FM25 primers (Table S5) were used. Cycling conditions were identical to the first round, except the temperature of the annealing step was 60°C. Reaction products were then separated by agarose gel electrophoresis.

LM-PCR of extra-chromosomal substrates from REH cells

Ligation reactions consisted of 5 μL Hirt extract, 60 pmol annealed DR19/DR20 linker (Roth et al., 1993), 3 μl 10x T4 DNA ligase buffer and 5 units T4 DNA ligase for 16 hours at 16°C. Samples were extracted twice with phenol/chloroform, ethanol precipitated and re-suspended in 30 μL H_2O. The amount of each episome was then determined via qPCR using the normalization primers described above for the RAG cutting assay. Samples containing equivalent amounts of each episome were then used in LM-PCR reactions and the presence of the template further verified by regular PCR using the normalization primers.

First round LM-PCR reactions contained normalized amounts of ligation product, 200 μM dNTPs, 0.2 μM of each primer (the relevant forward primer and DR20 linker primer), 2.5 μl 10x ThermolPol buffer and 0.625 units Taq DNA polymerase (NEB) in 25 μL final volume. The second round, semi-nested PCR reactions contained 0.5 μl first round product, 400 μM dNTPs, 1 μM of each primer, 2.5 μl 10x ThermolPol buffer and 1.5 units Taq DNA polymerase (NEB) in 25 μL final volume. Following an initial denaturation at 95°C for 5 minutes, cycling conditions consisted of 95°C for 20 s, Tm for 20 s and 72°C for 15 s for the optimized number of cycles (below) for each LM-PCR reaction. An aliquot (10 μl) of the PCR product was then digested with ApaLI for 5 hours at 37°C. Undigested and digested products were then electrophoresed on a 2% agarose gel. Due to the difficulty in designing optimal primers that also differentiate between RSS and SJ-containing episomes, (which are in the same vector backbone), some non-specific LM-PCR products are visible. However, the expected LM-PCR products were verified by restriction mapping using relevant enzymes in addition to ApaLI.

Primers (Table S5) and cycling conditions for each of the RSS or ESC ends were as follows: 12-RSS, first round PCR was for 23 cycles at 59.7°C using 12-RSS 1st round plus DR20 while the second round was for 27 cycles at 63.5°C using 12-RSS 2nd round plus 2nd round reverse. 23-RSS, first round PCR was for 16 cycles at 61.5°C using 23-RSS/SJ23d9-12-RSS 1st round plus DR20 while the second round was for 28 cycles at 66°C using 23-RSS 2nd round plus 2nd round reverse. The 12-RSS end of the SJ was amplified using a first round PCR of 19 cycles at 59.7°C using SJ-12-RSS/SJ12d7-23-RSS 1st round plus DR20 whereas the second round was for 32 cycles at 65°C using SJ-12-RSS 2nd round plus 2nd round reverse. The 23-RSS end of the SJ was amplified using a first round PCR of 16 cycles at 63.5°C using SJ 23-RSS 1st round plus DR20 while the second round was for 28 cycles at 65°C using SJ-23-RSS 2nd round plus 2nd round reverse. Since two of the mutated SJs were inserted into the plasmid in the opposite orientation, amplification of the SJ23d9-12-RSS end was for 26 cycles at 63°C using 23-RSS/SJ23d9-12-RSS 1st round plus DR20 whereas the second round was for 26 cycles at 65°C using SJ-12-RSS 2nd round and 2nd round reverse. Amplification of SJ12d7-23-RSS end was for 24 cycles at 59.7°C using SJ12-RSS/SJ12d7-23-RSS and DR20 while the second round was for 25 cycles at 65°C using SJ-23-RSS 2nd round and 2nd round reverse.

The standard PCRs to check the normalized levels of episomes in the ligation mix containing 40 fg of template (as determined by qPCR), 400 μM dNTPs, 0.2 μM of each of the respective normalization primers as used for the RAG cutting assay, 2.5 μl 10x ThermolPol buffer and 3 units Taq DNA polymerase (NEB). PCR reactions were for 28-31 cycles, depending on the primer efficiency, and the products were resolved on a 2% agarose gel.

LM-PCR to detect broken Vλ1-JCλ1 signal ends

LM-PCR to detect broken ESC ends was carried out in the same way as for NIH 3T3 cells except that 5 ng of genomic DNA from pro-B cells from PIP3 transgenic mice was used in the ligation reaction. Control samples were digested with ApaLI and the end was filled in with Klenow fragment of DNA polymerase I prior to ligation of the annealed FM25/FM11 oligonucleotides. Ligated products were amplified by 30 cycles of PCR using the primers Vend1 or Jend1 for the V and J ends, respectively together with primers that consisted of 18 bp of the 25 bp linker extended 7 bp into the sequence at the V or the J end of the ESC (Vendnested and Jendnested; Table S5). Since the ends of ApaLI cut products were filled in, compared to the blunt ends expected at the ESC, ApaLI cut samples were amplified with an appropriately altered primer (VendApaLI and JendApaLI). PCR products were electrophoresed on a 2% agarose gel.

Quantification of ligated signal ends

DNA was isolated from pro-B cells from PIP3 mice and pre-B cells of non-transgenic mice as described (Bevington and Boyes, 2013). For Vλ1Jλ1, approximately 3 ng of DNA was PCR amplified using primers pairs that amplify across the ESC junction (Jend3 and Vend2nd; Table S5) or the predicted Vλ1Jλ1 recombination product (V1Sac1F and J1StyR; Table S5). The PCR products were quantified using the QuantiFluor dsDNA System (Promega) and were diluted to generate standard curves with known numbers of copies each over a 10^4-fold range. Equivalent amounts of DNA samples from pre-B cells were then used in qPCR reactions to determine the copies of ESC and recombination product per cell. For the ESC, a single round of qPCR was carried out using the primers Jend3 and Vend2nd. For the Vλ1Jλ1 recombination product, a first round of amplification was carried out on both the pre-B DNA sample and the standard curve using the primers V1Sac1F and J1StyR, followed by qPCR of 1.5 μL of 1:10 diluted first round PCR product using the primers V1RSSF and J1RSSR. Absolute copies of ESC and recombination were then calculated from the standard curves, which allowed the ratio of intact ESC to be calculated.

The ESC and recombination products of Vκ3Jκ5 and Vκ16Jκ5 were detected in the same way except two rounds of PCR were performed in all cases using 1 μL of a 1:10 dilution of the first round product. The first round amplifications of the Vκ3Jκ5 and Vκ16Jκ5 ESC junctions used the primers Vk3 end first or Vk16 end first with Jk5 end first (Table S5) for 18 cycles of 94°C for 10 s, 53°C for 20 s and 72°C for 20 s. The second round qPCR reaction used Vk3 end second or Vk16 end second with Jk5 end

second and an annealing temperature of 60°C. To amplify the recombination junctions, primers Vk3 cdnd first or Vk16 cdjt first were used with Jk5 cdjt first under the same conditions used to amplify the ESC except the annealing temperature was 55°C. The second round qPCR reaction used Vk3 cdnd second or Vk16 cdnd first with Jk5 cdnd first and an annealing temperature of 60°C.

Detection of γH2AX foci by immunofluorescence

Cos7 cells were plated at a density of 1 × 10^5 per well in 6-well plates on poly-L-lysine coated coverslips. They were transfected 24 hours later with 0.5 μg of p12+, p23+, pSJ12d9 or pSJ+ plasmids with or without 1 μg of expression vectors for RAG1 and RAG2. Cells were stained for γH2AX foci after 24 hours by fixing in 4% paraformaldehyde for 10 minutes, followed by permeabilisation with 1% Triton X-100. After washing, non-specific binding was blocked by incubation with 1% BSA for 1 hour at 37°C. Antibody hybridization was carried out using anti phospho-histone H2A.X (Ser139) antibody (2577, Cell Signaling Technology), diluted 1:800 for 1 hour at 37°C, followed by washing and hybridization with AlexaFluor488 anti-rabbit IgG (Cell Signaling Technology), diluted 1 in 500 in 1% BSA. Following washing, the coverslip was mounted using Vectashield plus DAPI (Vector Laboratories). Images were recorded using a Zeiss LSM880 confocal microscope. REH cells were transduced with EGFP-expressing lentiviruses that either carry no additional sequence or the 23-RSS, SJ12d9 or SJ by spinfection at 800 x *g* for 30 minutes. After 9 hours, cells were spun onto a poly-L lysine coated coverslip at 800 x *g* for 30 minutes and incubated overnight. Following fixing in 4% paraformaldehyde (v/v) in PBS, γH2AX foci were detected as above except that blocking was with 5% BSA and an AlexaFluor568 (red)-labeled secondary antibody (Invitrogen) was used (at 1:400 dilution).

Detection of apoptosis

REH cells were seeded at 1 × 10^6 in a 12-well dish and transduced as above. Cells were harvested after 24 hours and labeled with APC-conjugated Annexin V (Thermo Fisher) and stained with propidium iodide, according to the instructions of the Annexin V Apoptosis detection kit. Labeled cells were detected by flow cytometry using a LSRFortessa (Becton Dickinson).

LAM-HTGTS assay

To detect translocations between a CRISPR/Cas9 induced bait DSB and potential RAG mediated DSBs, LAM-HTGTS was performed as described (Hu et al., 2016), with minor modifications. CRISPR/Cas9 guide RNAs (gRNAs) were designed against two bait sites in the human genome: *ROSA26* (CTGTCACAAGGCCGCGAGAA, hg19 chr3:9440072-9440091) and *IgH* (ATATTCCACC CAGGTAGTGG, hg19 chr14:106326860-106326879) and were cloned into LentiCRISPR-v2, a gift from Feng Zhang (Addgene plasmid # 52961), an all-in-one vector that expresses both the guide RNA (gRNA) and Cas9.

Lentiviruses containing the 12-RSS, 23-RSS, SJ12d9, SJ and control vector were produced as above using cell culture supernatants that were filtered and used directly without concentration. LentiCRISPR-v2 lentiviruses were produced using the wild-type pR8.74 packaging plasmid, producing integrase competent viruses to allow high level CRISPR/Cas9 expression.

For experiments containing nuclease only (Cas9 only, N = 7 for *ROSA26* and N = 3 for *IgH*), nuclease and control vector (Cas9+pWPI, N = 3 for *ROSA26* and *IgH*) or nuclease and RSS, mutant SJ or SJ (Cas9+12-RSS, Cas9+23-RSS, Cas9+SJ12d9, all N = 3 for *ROSA26* and Cas9+SJ, N = 12 for *ROSA26* and N = 4 for *IgH*), 4 X 10^6 REH cells were transduced with 1.2 mL LentiCRISPR-v2 virus containing the appropriate gRNA. For Cas9+pWPI and Cas9+RSS or Cas9+SJ experiments, REH cells were transduced with equivalent titers of pWPI (control virus), RSS or SJ virus, produced using the mutant integrase deficient D64V pCMVR8.74 packaging plasmid, at 18 hours. In instances where < 10% of the cells were transduced, a further transduction was performed at 42 hours after the initial transduction. All transductions were centrifuged at 800 x *g*, 32°C, for 30 minutes in the presence of 0.4 μg/ml polybrene (TR-1003-G, Merck). Seventy-two hours after the initial transduction, cells were harvested and genomic DNA prepared. For negative control experiments (without nuclease and RSS or SJ), genomic DNA was extracted directly from uninfected REH cells, as described (Hu et al., 2016).

LAM-HTGTS was performed as described (Hu et al., 2016), using 80 μg input DNA. Equimolar amounts of individual libraries were pooled for sequencing (2 × 250 bp) on an Illumina Miseq using a 500V2 kit (Illumina). Fastq files were demultiplexed and analyzed for translocation junctions using the tools provided in Hu et al., 2016. The sequence ± 100 bp of each translocation was then analyzed for potential RSS motifs using Recombination Signal Sequences Site (accessible at: https://www.itb.cnr.it/rss/) (Cowell et al., 2004). Potential 12-RSSs and 23-RSSs were analyzed, and the number of RSSs passing the recombination sequence information content (RIC) score threshold were compared between Cas9 only, Cas9+pWPI, Cas9+12-RSS, Cas9+23-RSS, Cas9+SJ12d9 and Cas9+SJ experiments. The RIC score is the log probability of observing the RSS sequence in a population of human RSSs and ranges from 0 to −1000, where a score of 0 is best. Scores of > −38.81 and > −58.45 for a 12-RSS and 23-RSS, respectively, pass the filter. RSS motif analysis was automated using an in house Python script.

To determine if the presence of a SJ leads to a significant increase of translocations to RSSs, a one tailed chi-square test of association (with Yates' continuity correction) was used. A large number of translocations to the *EEF1A1* promoter region (chr6:74,222,954-74,233,993) were observed in all datasets and our analyses exclude these translocations. We believe this is more representative as the apparent translocations to the *EEF1A1* promoter region is most likely due to the integration of EF1α promoter sequences from the RSS, SJ and LentiCRISPR-v2 lentiviruses at the Cas9-mediated break-points. Indeed, DSBs have been shown to capture exogenous DNA via a mechanism that does not involve sequence homology (Gabriel et al., 2011). A similar phenomenon was observed with the 12-RSS vector where a large number of translocations appear to originate from chromosome 7.

Since this corresponds exactly to the chromosome origin of the TCRβ 12-RSS sequence used in the lentiviral vector (chr7:142,495,018-142,495,281), these apparent translocations were excluded from our analysis of the 12-RSS data. In the absence of CRISPR/Cas9, we detect ~1500 translocations, approximately 10-fold fewer than in the presence of CRISPR/Cas9, similar to the relative levels previously reported by Hu et al. (2016).

To find overlaps between LAM-HTGTS translocations and genes which were observed to acquire somatic mutations in *ETV6-RUNX1*-positive ALL patients (Papaemmanuil et al., 2014), an in house Python script was used. The same strategy was also used to detect overlaps within 100 bp of LAM-HTGTS translocations and structural variations found in *ETV6-RUNX1*-positive ALL patients (Papaemmanuil et al., 2014). Potential RSS motifs in 1 kb windows centered on translocations overlapping patient breakpoints were scanned for using Recombination Signal Sequences Site (as above). Potential RSS heptamer motifs were scanned using a position weight matrix (PWM) generated from an RSS conservation table from Hesse et al. (1989) using the software tool FIMO, part of the MEME suite (version 4.12.0) (Grant et al., 2011). A background model of 20% C/G and 30% A/T, a pseudocount of 1 and a threshold p value of 0.001 were used.

QUANTIFICATION AND STATISTICAL ANALYSIS

A two-sample (unpaired), two-tailed Student's t test was used to determine the statistical significance of differences between sample means of % γH2AX foci in Cos7 and REH cells as well as % apoptosis in the presence of control virus, RSS or mutant SJ virus versus SJ virus. The same test was used to determine the statistical significance of differences between sample means of % SE release in the presence of the SJ partner versus the 23-RSS partner; * indicates $p \leq 0.05$; ** $p \leq 0.01$ and *** $p \leq 0.001$. A one tailed chi-square test of association (with Yates' continuity correction) was used to determine the statistical significance of differences between pairs of LAM-HTGTS data-sets with respect to chromosome translocation distribution and the presence/absence of RSSs at the chromosome breakpoints.

The significance of the co-localization of breaks detected by LAM-HTGTS and genes that are frequently mutated in ALL was calculated by using the hypergeometric distribution (implemented in the R software, https://www.r-project.org) to analyze the overlap (k genes) of the lists of ALL associated genes (N genes) and the genes identified as being involved in potentially SJ-mediated translocations (M genes). The p value is the probability of observing an overlap $\geq$ k with null hypothesis that the lists are unrelated.

The significance of the overlaps of breaks detected by LAM-HTGTS with those found in patients was tested using the Poisson distribution with the null hypothesis that patient breaks occur randomly over the genome with no relation to the breaks observed in the LAM-HTGTS data. An overlap was defined to occur if a LAM-HTGTS breakpoint occurred within 100 base pairs of a patient breakpoint. With the null hypothesis, the expected number of overlaps is the Poisson parameter $\lambda = kM/N$ where k is the number of patient breakpoints, N the size of the genome (in bases) and M the number of bases in the genome covered by LAM-HTGTS breakpoints, each extended by 100 bp on each side to account for the overlap condition.

DATA AND SOFTWARE AVAILABILITY

The LAM-HTGTS data have been uploaded as FASTQ files to NCBI SRA: PRJNA483469. The bespoke Python scripts to determine the presence of RSSs in the vicinity of each translocation, the chromosome distribution of translocations, the analysis of overlaps between translocations and target genes as well as the intersection between ALL patient breakpoints and translocations, have been deposited to GitHub: https://github.com/boyeslab/cutandrun.

Molecular Cell, Volume *74*

Supplemental Information

Cut-and-Run: A Distinct Mechanism

by which V(D)J Recombination

Causes Genome Instability

Christopher M. Kirkham, James N.F. Scott, Xiaoling Wang, Alastair L. Smith, Adam P. Kupinski, Anthony M. Ford, David R. Westhead, Peter G. Stockley, Roman Tuma, and Joan Boyes

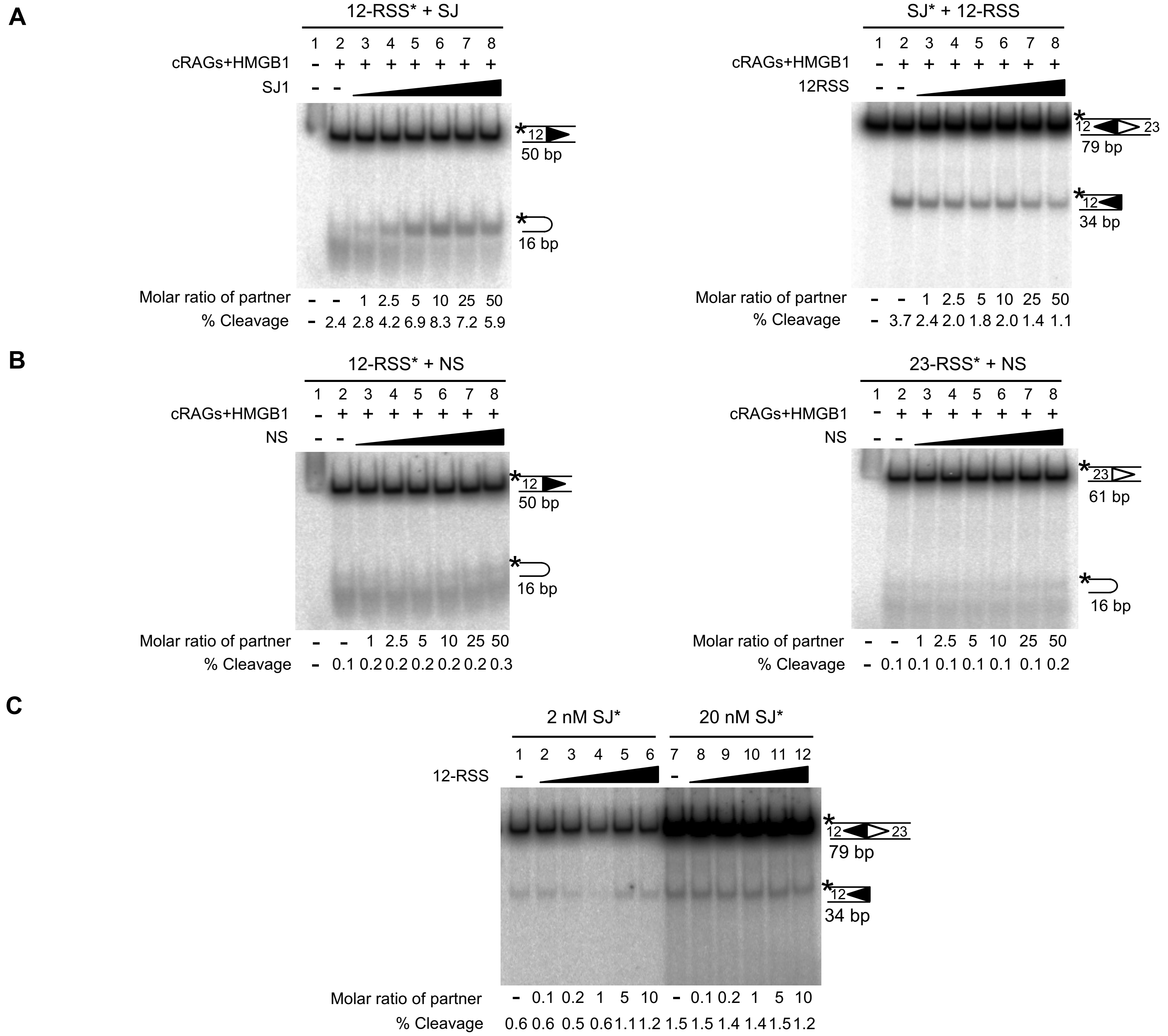

Supplementary Figure 1

Supplementary Figure Legends

Figure S1 – Asymmetric Cutting Occurs Under Different Conditions, Related to Figure 1. (A) Increasing amounts of unlabelled partner were added to an *in vitro* cleavage assay, as indicated by the filled arrow. This showed that i) cleavage is asymmetric over a wide range of partner ratios, and ii) that cleavage is greatest at a 10-fold excess of partner. The labelled oligonucleotide is indicated by an asterisk above each set of lanes. **(B)** Titration of a non-specific oligonucleotide does not enhance RAG cutting. Increasing amounts of an oligonucleotide that lacks RSS sequences (NS) were added to an *in vitro* cleavage assay, as indicated. **(C)** SJ cleavage by RAG proteins is not limited by the amount of substrate. The amount of labelled SJ was increased over a 10-fold range (lanes 1-6 versus 7-12) with no increase in per cent cutting. Titration of a partner RSS (indicated by the closed arrow) also did not increase the per cent cutting.

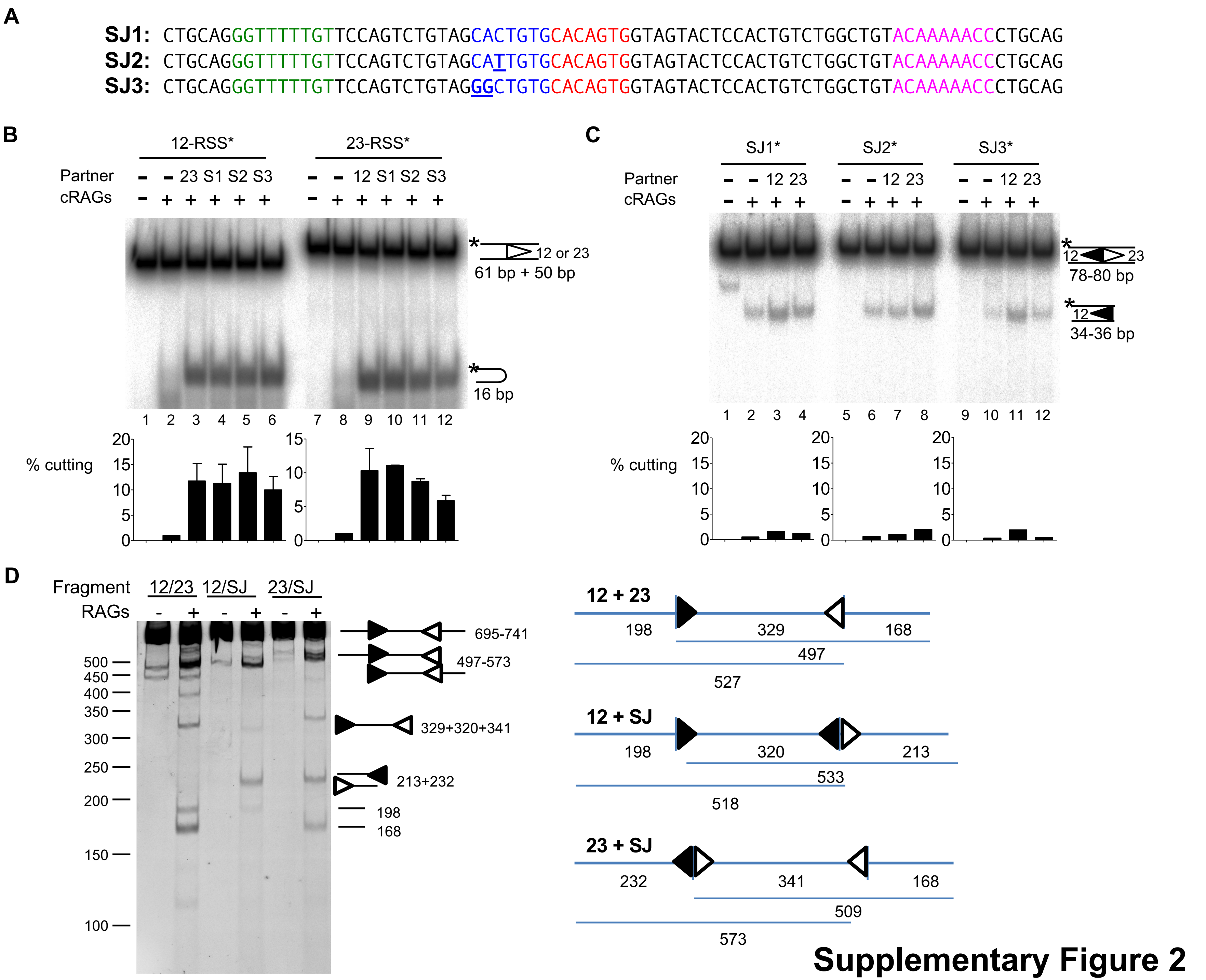

Supplementary Figure 2

Figure S2 – SJ cleavage is asymmetric with different SJ sequences, Related to Figure 1. (A) Comparison of the SJ sequences used. Bases that differ from consensus heptamer sequences are shown in bold. Blue = 12-RSS heptamer, green = 12-RSS nonamer, red = 23-RSS heptamer, magenta = 23-RSS nonamer. **(B)** SJs with common heptamer sequences (SJ2 and SJ3) were used in RAG cleavage reactions as partners for labelled 12 or 23-RSSs or **(C)** were radiolabelled and used as substrates with 12- and 23-RSS partners. Labelled oligonucleotides are indicated by an asterisk. See also Table S1 for frequently generated human ESCs. **(D)** The plasmid pJH290, which contains two RSSs 329 bp apart in a convergent orientation, was modified to contain a SJ in place of either the 12- or 23-RSS. Fragments with two complementary RSSs or an RSS plus SJ were PCR amplified from the plasmids, gel purified and used in RAG cutting assays (left). Following electrophoresis, DNA was stained with dsGreen. The expected product sizes for complete cleavage are shown to the right (12+23: 168+198+329 bp; 12+SJ: 198+213+320 bp; 23+SJ: 168+232+341 bp). Filled triangles = 12-RSS; open triangles = 23-RSS. The low level of cutting of the 12-RSS in pJH290 has been reported previously (Tevelev and Schatz, 2000).

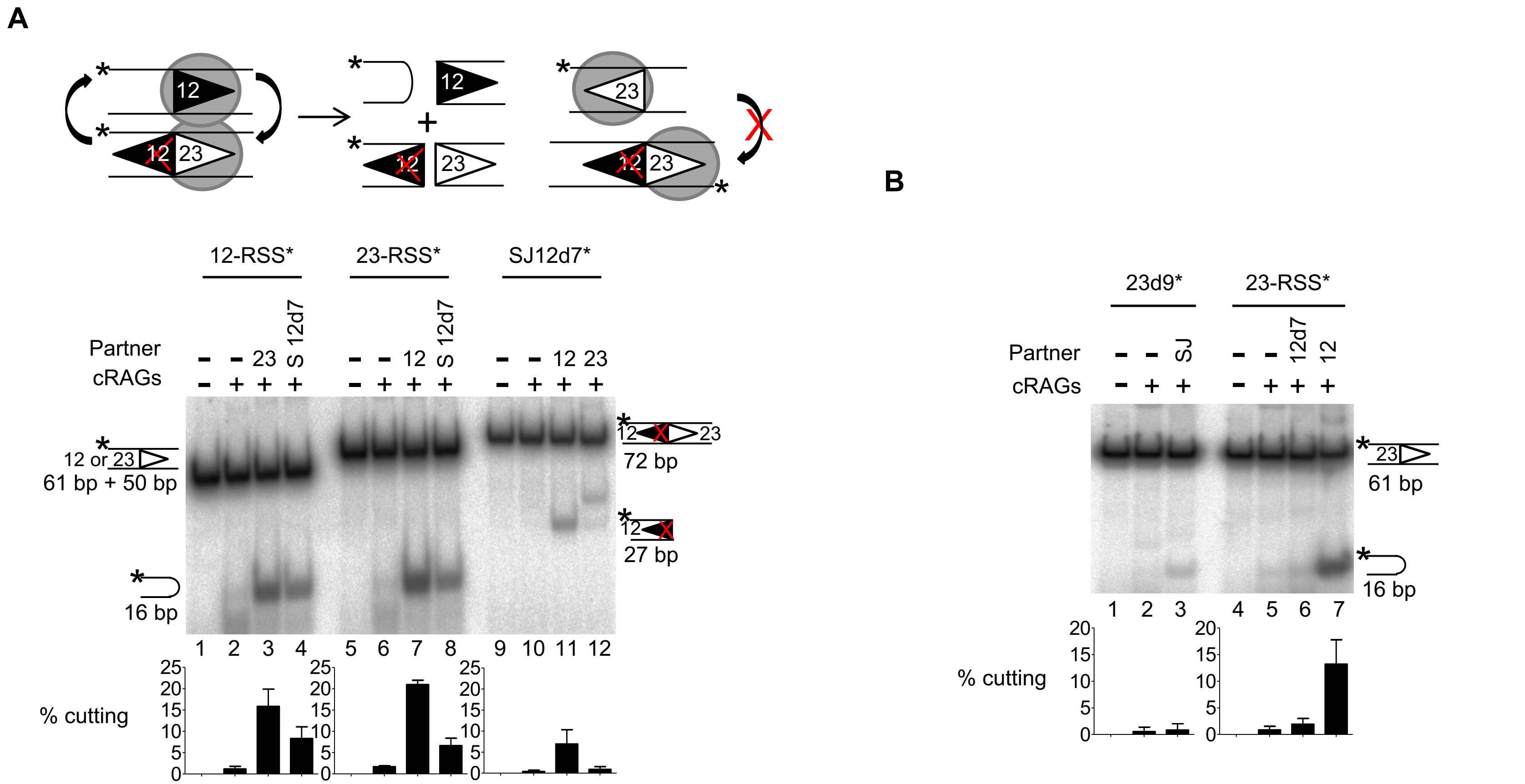

Supplementary Figure 3

Figure S3 – Deletion of the 12-RSS Heptamer within the SJ Restores Symmetric Cleavage, Related to Figure 3. (A) The 12-RSS heptamer within the SJ was deleted and the oligonucleotide used either as a partner for labelled 12- or 23-RSSs or as the substrate. Labelled oligonucleotides are denoted by an asterisk. Symmetrical cutting is restored with a 12-RSS (lanes 4 and 11). Enhanced cutting of SJ12d7 was observed when it was paired with a 23-RSS, to generate a product that is 7 bp bigger than that generated by cutting at the RSS/RSS boundary. This is likely due to the single heptamer being recognised in conjunction with both the 12 bp and 23 bp spacer and cutting occurring at the 3' end of the heptamer when it is paired with a 23-RSS. Graphs represent mean of ≥3 experiments ± SD. **(B)** The SJ mutations significantly reduce RAG cutting. An assumption of the experiments in Figures 3A, 3B and S3A is that mutating either the conserved heptamer or nonamer elements renders that "side" of the SJ unusable. To test this, we used a 12-RSS oligonucleotide where the heptamer sequence is mutated, essentially leaving a nonamer. Since the 12d7 oligonucleotide lacks a heptamer to direct RAG cutting, it was used as a partner with a 23-RSS. As expected, this oligonucleotide was unable to stimulate cutting at a 23-RSS (lane 6). Likewise, no cleavage is detected following mutation of the nonamer within a 23-RSS oligonucleotide (lanes 2-3). Graphs represent mean of four experiments ± SD.

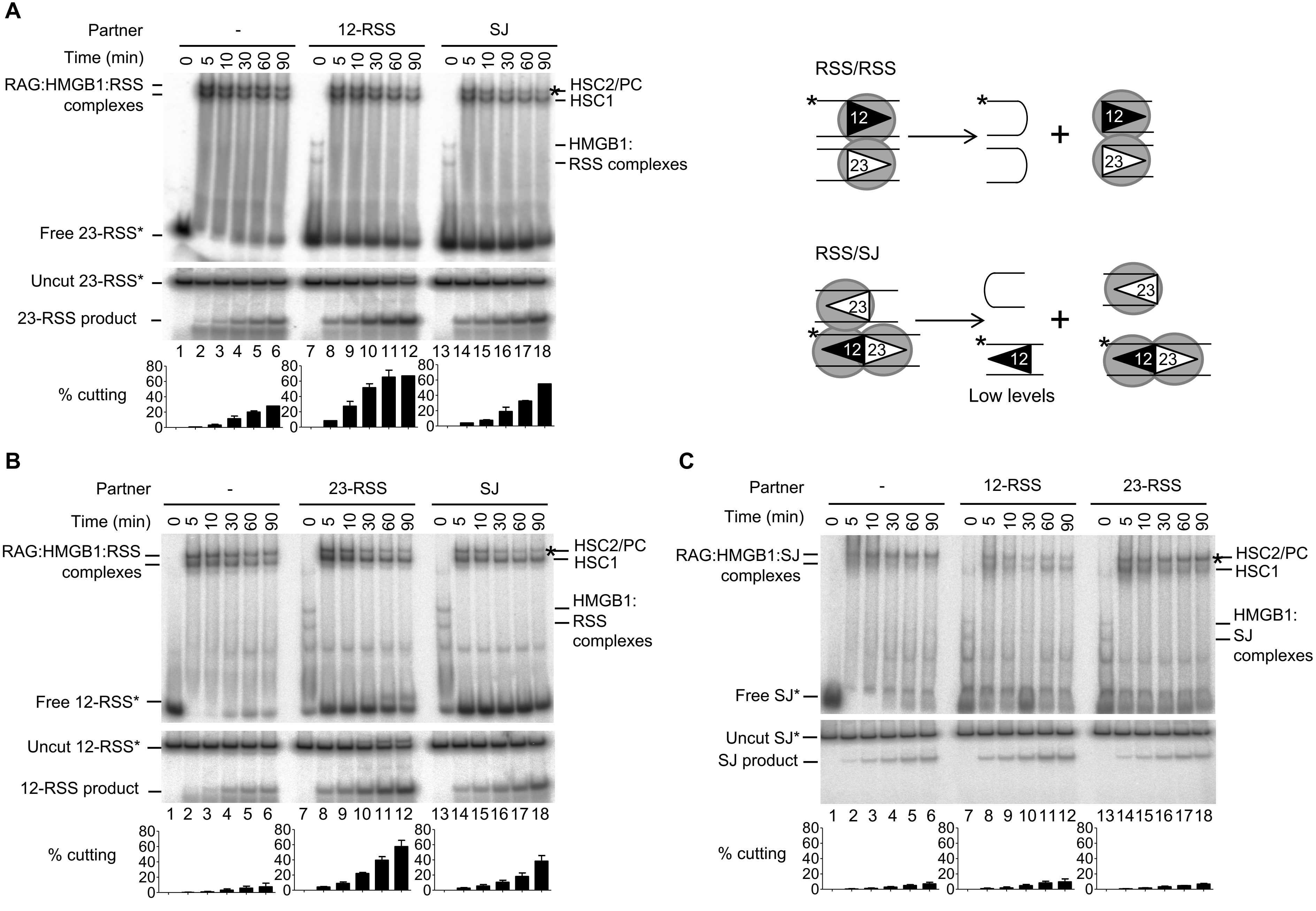

Supplementary Figure 4

Figure S4 – The Coding End of an RSS is Released by RAG Proteins Following Cleavage, Related to Figure 4. RAG cleavage assays were carried out in the presence of Mg^{2+} and the partner indicated with **(A)** a 23-RSS, **(B)** a 12-RSS and **(C)** a SJ. Samples were taken at the times indicated. Half the reaction was run on a 4% polyacrylamide gel to observe complex stability (upper gels), and half was run on a 12% native polyacrylamide gel to observe RAG cutting (lower gels). The HSC2 complex is indicated by an asterisk. In the absence of a partner RSS or SJ, little RAG cutting occurs (lower gels) and the RAG/ labelled RSS complex is stable (lanes 1-6). Upon addition of either a RSS or SJ partner, RAG cutting is enhanced and stability of the HSC2 complex is decreased at an RSS (parts A and B, middle and right panels) but not a SJ (part C; lanes 6-12 and 13-18). With a labelled SJ, complex C shown previously (Figure 2) is not consistently observed. A possible reason for this is that previously, Ca^{2+} was used as the divalent cation to observe precleavage synaptic complexes (Hiom and Gellert, 1997) whereas here, Mg^{2+} was used to facilitate cleavage, which possibly destabilises complex C. Graphs represent mean of three experiments $\pm$ SD.

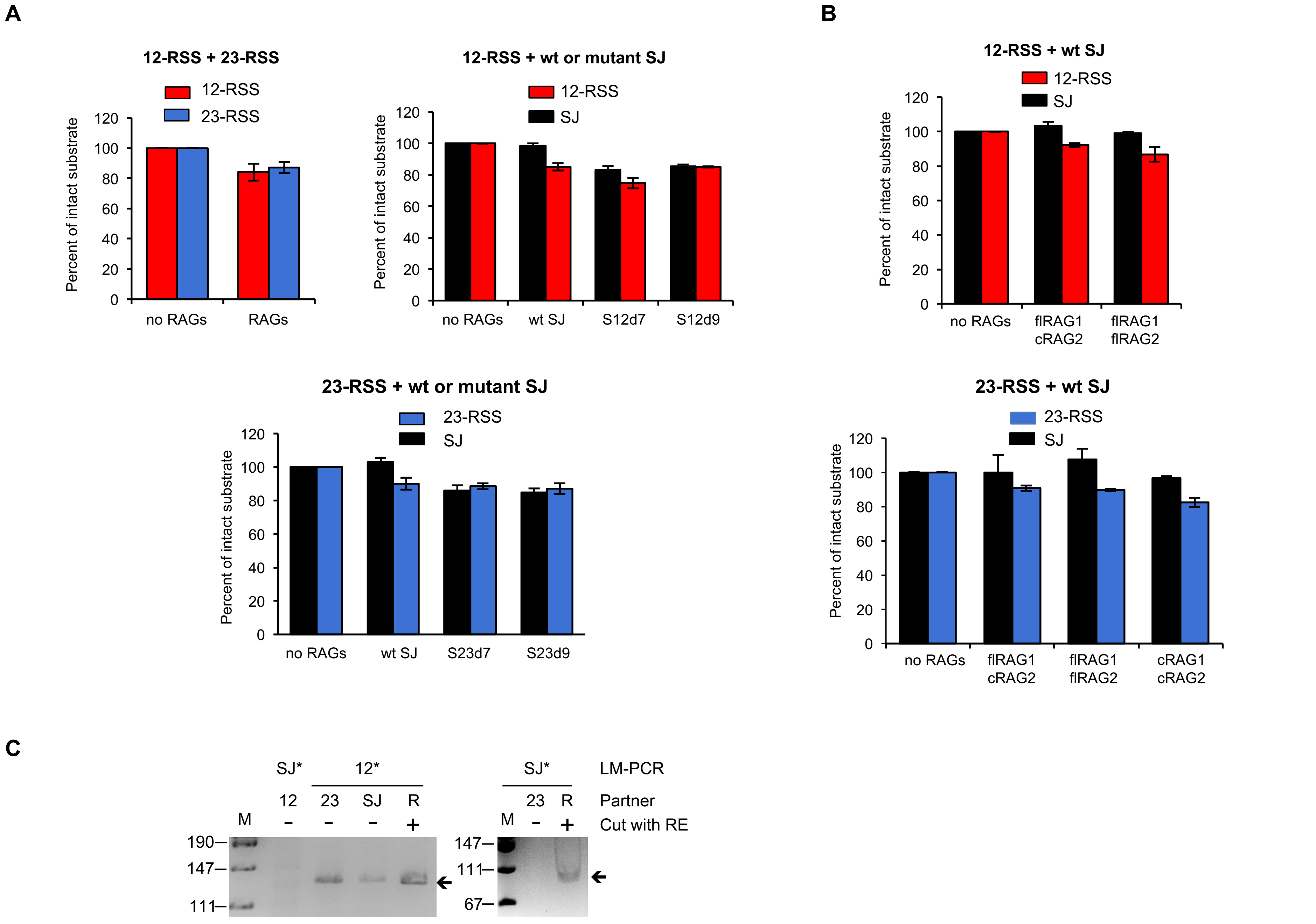

Supplementary Figure 5

Figure S5 – Asymmetric Cutting in SJ-RSS Pairs *in vivo,* Related to Figure 5. A) NIH3T3 cells were transfected with expression vectors for full length RAG1 and full length RAG2, and two substrate plasmids, as indicated. The amount of intact substrate after 48 hours was measured by qPCR. Data were normalised to unique regions within each plasmid, and the values given are relative to no RAG2 controls. Data are represented as mean of three experiments ± SD. **B)** As for (A) except that expression vectors for full-length (fl) RAG1 and RAG2 or core (c) RAG1 and RAG2 were used together with two substrate plasmids, as indicated. Data are represented as mean of three experiments ± SD. **C)** LM-PCR of extra-chromosomal substrate cleavage products. The plasmid samples used in (A) were used in an LM-PCR assay to verify that DSBs are formed. The RSS or SJ element that was amplified by LMPCR is indicated by an asterisk above each gel and the element on the partner plasmid is indicated by “Partner”. A product is detected only with plasmids carrying a 12-RSS and not those carrying a SJ. R indicates positive control samples that were cut with a restriction enzyme (RE; EcoRV for the 12-RSS and ApaLI for the SJ); arrows indicate products of the expected sizes (128 bp for 12-RSS & 92 bp for SJ).

A

Bait DSB site	Experiment	Total translocations	RSS	Not RSS	RSS fraction	Fold increase vs Cas9 only	Fold increase of SJ-mediated breaks at RSSs vs experiment	Significance of SJ-mediated breaks at RSSs vs experiment
ROSA26	Cas9 only	69824	12079	57745	0.172992094	1	1.131700211	p<0.0001
	Cas9 + pWPI (Control virus)	20641	3830	16811	0.185553026	1.072609856	1.055090259	p=0.0004
	Cas9 + 12-RSS	23591	4334	19257	0.183714128	1.061979907	1.065651247	p<0.0001
	Cas9 + 23-RSS	31993	5887	26106	0.184009002	1.063684457	1.063943545	p<0.0001
	Cas9 + SJ12d9	28252	5682	22570	0.201118505	1.162587837	0.973432007	ns
	Cas9 + SJ	106561	20862	85699	0.19577519	1.131700211	1	-
IgH	Cas9 only	9821	1616	8205	0.164545362	1	1.262257139	p<0.0001
	Cas9 + pWPI (Control virus)	6376	1163	5213	0.18240276	1.108525687	1.138681001	p<0.0001
	Cas9 + SJ	9846	2045	7801	0.207698558	1.262257139	1	-

B

	ROSA						IgH		
**Experiment**	**Cas9 only**	**Cas9 + pWPI (empty virus)**	**Cas9 + 12-RSS**	**Cas9 + 23-RSS**	**Cas9 + SJ12d9**	**Cas9 + ESC**	**Cas9 only**	**Cas9 + pWPI (empty virus)**	**Cas9 + SJ**
**Bait site chromosome**	41737	10789	12687	17680	13542	51321	5862	3294	4006
**Other chromosomes**	28087	9852	10904	14313	14710	55240	3959	3082	5840
**Fraction bait chromosome**	0.59774576	0.52269754	0.53778984	0.552621	0.479329	0.481611	0.59688423	0.51662484	0.40686573
**Significance of SJ-mediated breaks on other chromosomes vs experiment**	p<0.0001	p<0.0001	p<0.0001	p<0.0001	ns	-	p<0.0001	p<0.0001	-

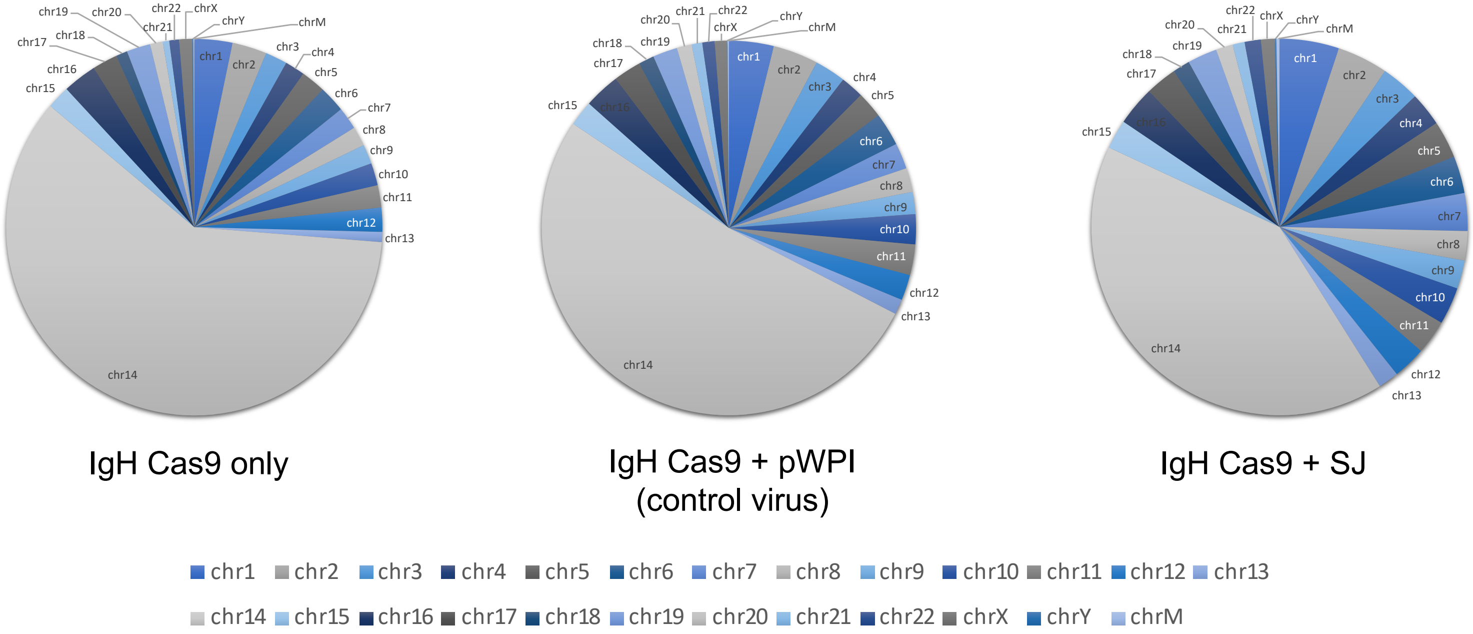

Supplementary Figure 6

Figure S6 – Translocations to RSSs are Increased in the Presence of the SJ, Related to Figure 7. (A) Table showing total number of translocations at the *ROSA26* and *IgH* loci to RSSs, as defined by RSS Site. A sequence is scored as an RSS by RSS Site by its similarity to physiological human RSSs. It is possible that yet more sequences are used as RSSs *in vivo*. Since rare cutting events at relatively poor RSSs are likely to be detected by the translocation assay, the calculation of translocations to RSSs is likely to be conservative. The SJ and SJ12d9 vectors differ by only 7 bp and since there is some residual binding to both RSSs in SJ12d9, it is perhaps not surprising that it also causes breaks at RSSs. The greater effect of SJ12d9 in cutting RSSs compared to the generation of γH2AX foci may be due to the γH2AX assay detecting all cuts whereas the LAM-HTGTS assay detects only the first cut but not subsequent ones due to "run". If the RAG-SJ12d9 complex is less stable, this may reduce subsequent cuts due to "run", giving rise to fewer γH2AX foci. **(B)** Table and pie charts of chromosomal distribution of translocations to the *ROSA26* and *IgH* loci in the presence and absence of the SJ. Many of the Cas9 only translocations are to the same chromosome as the break-point, suggesting that they might be the result of processing around the break-point. By contrast, the SJ causes translocations throughout the genome. Consistent with the idea that the SJ causes more breaks than a single RSS, a significant increase in chromosome distribution of breaks with the SJ is observed compared to the 12- and 23-RSS as well as the empty (control) virus (p <0.0001 in each case).

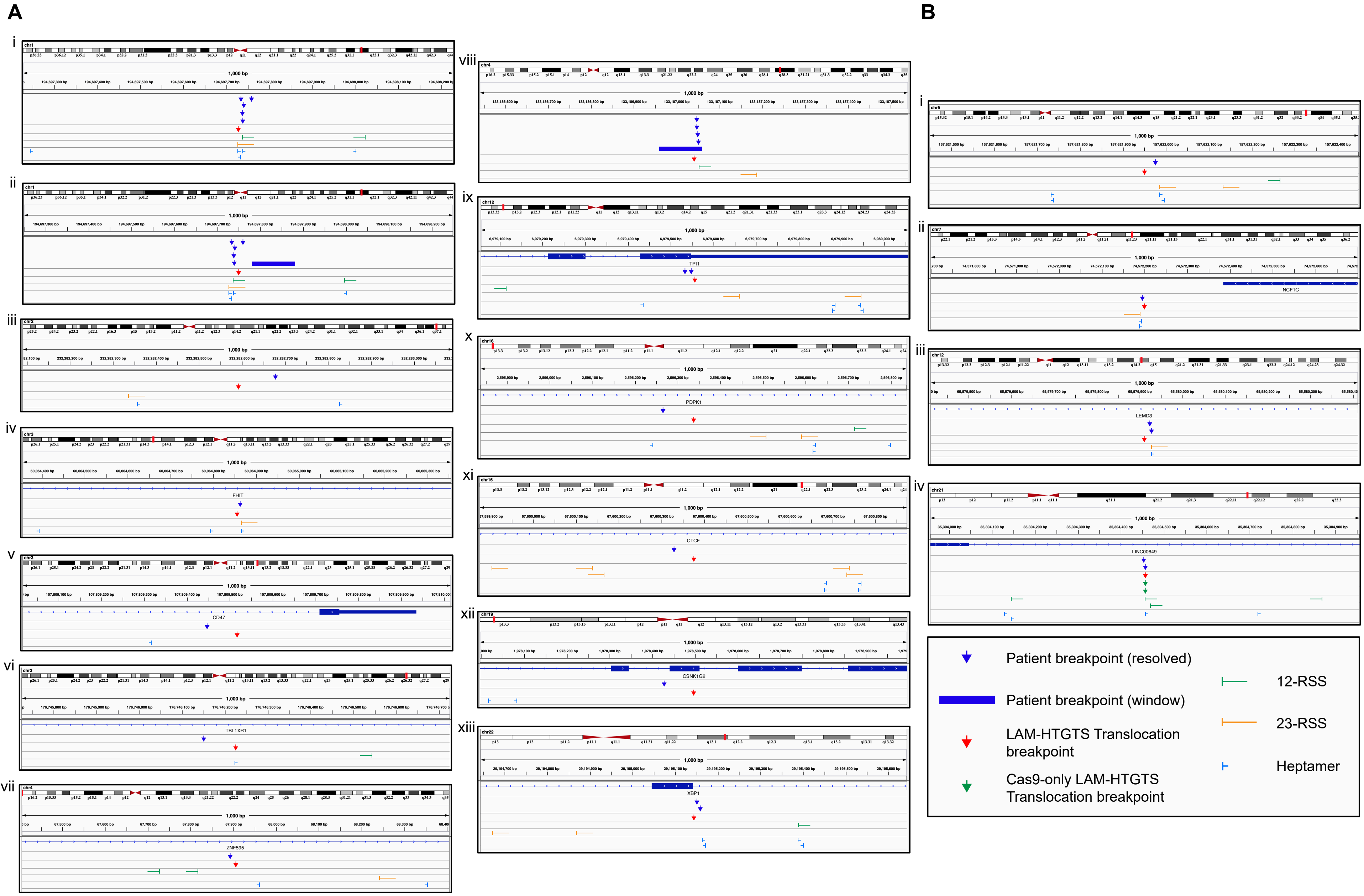

Supplementary Figure 7

Figure S7 – SJ-mediated Breaks Co-localise with those Found in ALL Patients, Related to Figure 7. A) Data showing 13 individual translocations identified by LAM-HTGTS in the presence of the SJ (red arrowheads) that lie within 100 bp of 28 breakpoints in ALL patients with the *ETV6/RUNX1* translocation (blue arrowheads; i-xiii). Some patient breakpoints were not fully resolved and these are represented by blue bars. Panels i and ii show two breaks detected by LAM-HTGTS that occur in close proximity on chromosome 1. These are shown separately since (i) overlaps with five patient breakpoints whereas (ii) overlaps with six. Heptamer and RSS data are taken from the FIMO and RSS Site web-sites, respectively (STAR Methods). **B)** Translocations detected in the presence of SJ12d9 (i-iv). A number of breaks is to be expected since single RSSs cause some genome instability (Han et al., 1999). One of the breakpoints in the presence of SJ12d9 (iv) is detected twice in cells transduced to express Cas9 only, suggesting that this particular break may be due to off-target Cas9 cleavage.

Table S1: Sequence Conservation of most Frequently Generated human ESCs, Related to Figure S2.

Locus	Recombination event	Number of recombination events	ESCs where both heptamers have the consensus sequence and AA is conserved in positions 5 and 6 of both nonamers (%)	ESCs where both heptamers have CAC and at least 6 out of 7 conserved nucleotides and AA is conserved in positions 5 and 6 of both nonamers (%)
Immunoglobulin Heavy Chain (*IgH*)	V to D	1512	49.70	86.24
Immunoglobulin Heavy Chain (*IgH*)	D to J	216	0.00	84.82
Immunoglobulin Kappa (*IgK*)	V to J	200	62.80	90.84
Immunoglobulin Lambda (*IgL*)	V to J	238	18.40	63.6
T Cell Receptor Alpha (*TCRA*)	V to J	2499	21.14	65.16
T Cell Receptor Beta (*TCRB*)	V to D	98	0.00	36.67
T Cell Receptor Beta (*TCRB*)	D to J	26	0.00	19.06
T Cell Receptor Gamma (*TCRG*)	V to J	40	4.22	5.46
T Cell Receptor Delta (*TCRD*)	V to D	6	14.40	20.00
T Cell Receptor Delta (*TCRD*)	D to J	8	0.00	0.00
**Total**		**4843**	**29.98**	**72.12**

Data weighted based on IMGT database of human gene segment usage

Table S2: Overlap between patient breakpoints and translocations mapped via LAM-HTGTS, Related to Figures 7 and S7

Translocation	Translocation chr	Translocation breakpoint	Translocation orientation	Patient breakpoint ID	Breakpoint position	Patient sample ID	RSS within 500 bp	Heptamer within 500 bp
**Cas9**								
1	chr12	123075587	-	37612940	123075442-123075542	PD4013a	Yes	Yes
2	chr20	33412439	-	37733808	33412465	PD4028a	Yes	Yes
3	chr21	35304456	-	37724900	35304456	PD3958a	Yes	Yes
				38057914	35304453	PD4038a		
4	chr21	35304456	-	37724900	35304456	PD3958a	Yes	Yes
				38057914	35304453	PD4038a		
**pWPI**								
1	chr16	69169672	+	37719304	69169579	PD4010a	Yes	Yes
**12-RSS**								
1	chr19	37019097	-	37718050	37019115	PD4030a	Yes	Yes
**23-RSS**								
1	chr8	133765911	+	37446514	133765977	PD3956a	Yes	Yes
2	chr11	36638045	+	37821575	37821575	PD4016a	No	Yes
				37611808	37611808	PD4013a		
				37700046	37700046	PD3958a		
3	chr14	93812808	-	37706408	93812668-93812768	PD3961a	Yes	Yes
4	chrX	38421475	-	37456899	38421395-38421495	PD3968a	Yes	Yes
**SJ12d9**								
1	chr5	157621949	-	37441544	157621975	PD3964a	Yes	Yes
2	chr7	74572198	+	43011562	74572193	PD4020a	Yes	Yes
3	chr12	65579911	-	37519754	65579927	PD4022a	Yes	Yes
				37612853	65579923	PD4013a		
4	chr21	35304456	+	37724900	35304456	PD3958a	Yes	Yes
				38057914	35304453	PD4038a		

**SJ**								
1	chr1	194697727	-	37502102	194697737	PD3971a	Yes	Yes
				37750532	194697739	PD3960a		
				37639400	194697758	PD4018a		
				37526674	194697733	PD3953a		
				37631487	194697736	PD4025a		
2	chr1	194697749	+	37502102	194697737	PD3971a	Yes	Yes
				37750532	194697739	PD3960a		
				37639400	194697758	PD4018a		
				37526674	194697733	PD3953a		
				37631487	194697736	PD4025a		
				38581730	194697780-194697880	PD4035a		
3	chr2	232282590	-	37668781	232282677	PD4018a	Yes	Yes
4	chr3	60064855	-	37981423	60064861	PD4036a	Yes	Yes
5	chr3	107809516	-	37654082	107809446	PD4025a	No	Yes
6	chr3	176746225	-	37657164	176746149	PD4028a	Yes	Yes
7	chr4	67905	+	37516749	67891	PD4022a	Yes	Yes
8	chr4	133187040	-	37603732	133187048	PD4013a	Yes	No*
				37869991	133187050	PD4012a		
				38033843	133187046	PD4037a		
				37663849	133187046	PD4010a		
				37465888	133186959-133187059	PD3965a		
9	chr12	6979556	+	37703949	6979532	PD4010a	Yes	Yes
				37704018	6979547	PD4010a		
10	chr16	2596337	-	37736538	2596265	PD3957a	Yes	Yes
11	chr16	67600374	-	37716657	67600328	PD3973a	Yes	Yes
12	chr19	1978496	-	37718728	1978427	PD3958a	No	Yes
13	chr22	29195143	-	37457724	29195158	PD3959a	Yes	Yes
				37725243	29195150	PD4025a		

*As determined by FIMO motif finder; RSSs were determined by RSS Site

Table S3: Breaks in frequently mutated genes in ALL in the presence of the SJ, Related to Figure 7

	Translocations normalised per 100,000 translocations						SJ enrichment over test:				
	Cas9	pWPI	12-RSS	23-RSS	SJ12d9	SJ	Cas9	pWPI	12-RSS	23-RSS	SJ12d9
IKZF1	0.000	3.704	8.478	6.251	3.540	5.156	TRUE	TRUE	FALSE	FALSE	TRUE
NRAS	1.256	0.000	0.000	0.000	0.000	4.297	TRUE	TRUE	TRUE	TRUE	TRUE
KRAS	0.000	0.000	0.000	0.000	0.000	0.859	TRUE	TRUE	TRUE	TRUE	TRUE
PAX5	10.048	0.000	4.239	12.503	3.540	11.172	TRUE	TRUE	TRUE	FALSE	TRUE
CDKN2A	0.000	0.000	0.000	0.000	0.000	0.859	TRUE	TRUE	TRUE	TRUE	TRUE
FLT3	2.512	0.000	0.000	3.126	0.000	4.297	TRUE	TRUE	TRUE	TRUE	TRUE
JAK2	1.256	0.000	0.000	3.126	0.000	1.719	TRUE	TRUE	TRUE	FALSE	TRUE
TP53	1.256	0.000	4.239	0.000	3.540	1.719	TRUE	TRUE	FALSE	TRUE	FALSE
PTPN11	5.024	3.704	0.000	3.126	0.000	4.297	FALSE	TRUE	TRUE	TRUE	TRUE
CREBBP	11.304	29.629	21.195	6.251	10.619	18.047	TRUE	FALSE	FALSE	TRUE	TRUE
JAK1	2.512	0.000	4.239	3.126	3.540	5.156	TRUE	TRUE	TRUE	TRUE	TRUE
Number of genes enriched with SJ vs test:							10	10	8	8	10
Significance of SJ vs test:							0.00022	0.00029	0.021	0.018	0.00019

(B) Frequently mutated genes in *ETV6/RUNX1*-positive ALL

	Translocations normalised per 100,000 translocations						SJ enrichment over:				
	Cas9	pWPI	12-RSS	23-RSS	SJ12d9	SJ	Cas9	pWPI	12-RSS	23-RSS	SJ12d9
ETV6	0.000	3.704	8.478	0.000	0.000	3.438	TRUE	FALSE	FALSE	TRUE	TRUE
TBL1XR1	1.256	0.000	4.239	3.126	3.540	8.594	TRUE	TRUE	TRUE	TRUE	TRUE
PAX5	10.048	0.000	4.239	12.503	3.540	11.172	TRUE	TRUE	TRUE	FALSE	TRUE
ATF7IP	2.512	3.704	4.239	9.377	3.540	6.875	TRUE	TRUE	TRUE	FALSE	TRUE
BTG1	1.256	0.000	0.000	0.000	0.000	1.719	TRUE	TRUE	TRUE	TRUE	TRUE
RAG2	0.000	0.000	0.000	3.126	0.000	0.859	TRUE	TRUE	TRUE	FALSE	TRUE
BTLA	0.000	0.000	0.000	0.000	0.000	0.000	-	-	-	-	-
NR3C2	3.768	3.704	8.478	0.000	3.540	3.438	FALSE	FALSE	FALSE	TRUE	FALSE
CDKN2A	0.000	0.000	0.000	0.000	0.000	0.859	TRUE	TRUE	TRUE	TRUE	TRUE
KRAS	0.000	0.000	0.000	0.000	0.000	0.859	TRUE	TRUE	TRUE	TRUE	TRUE
STAG2	0.000	3.704	4.239	0.000	7.079	2.578	TRUE	FALSE	FALSE	TRUE	FALSE
ZMYM2	5.024	11.111	0.000	0.000	7.079	3.438	FALSE	FALSE	TRUE	TRUE	FALSE
MGA	3.768	7.407	0.000	6.251	3.540	8.594	TRUE	TRUE	TRUE	TRUE	TRUE
Number of genes enriched with SJ vs test:							10	8	9	9	9
Significance of SJ vs test:							0.0027	0.056	0.023	0.019	0.012

Table S4: Oligonucleotides used in RAG cutting assays, Related to Star Methods

Oligonucleotide	Sequence
12-RSS top (DAR39)	5'-GATCTGGCCTGTCTTACACAGTGCTACAGACTGGAACAAAAACCCTGCAG-3'
12-RSS bottom (DAR40)	5'-CTGCAGGGTTTTTGTTCCAGTCTGTAGCACTGTGTAAGACAGGCCAGATC-3'
23-RSS top (DG61)	5'-GATCTGGCCTGTCTTACACAGTGGTAGTACTCCACTGTCTGGCTGTACAAAAACCCTGCAG-3'
23-RSS bottom (DG62)	5'-CTGCAGGGTTTTTGTACAGCCAGACAGTGGAGTACTACCACTGTGTAAGACAGGCCAGATC-3'
SJ1 top (MBNSJ top)	5'-CTGCAGGGTTTTTGTTCCAGTCTGTAGCACTGTGCACAGTGGTAGTACTCCACTGTCTGGCTGTACAAAAACCCTGCAG-3'
SJ1 bottom (MBNSJ bottom)	5'-CTGCAGGGTTTTTGTACAGCCAGACAGTGGAGTACTACCACTGTGCACAGTGCTACAGACTGGAACAAAAACCCTGCAG-3'
SJ2 top	5'-CTGCAGGGTTTTTGTTCCAGTCTGTAGCA**T**TGTGCACAGTGGTAGTACTCCACTGTCTGGCTGTACAAAAACCCTGCAG-3'
SJ2 bottom	5'-CTGCAGGGTTTTTGTACAGCCAGACAGTGGAGTACTACCACTGTGCACA**A**TGCTACAGACTGGAACAAAAACCCTGCAG-3'
SJ3 top	5'-CTGCAGGGTTTTTGTTCCAGTCTGTAG**GG**CTGTGCACAGTGGTAGTACTCCACTGTCTGGCTGTACAAAAACCCTGCAG-3'
SJ3 bottom	5'-CTGCAGGGTTTTTGTACAGCCAGACAGTGGAGTACTACCACTGTGCACAG**CC**CTACAGACTGGAACAAAAACCCTGCAG-3'
Non-specific top (DAR81)	5'-GATCTCGCCTCTCTTAGGTTAATCCTATAGAACTCGTCCCCGTACCTCGAG-3'

Non-specific bottom (DAR82)	5’-CTAGAGCGGAGAGAATCCAATTAGGATATCTTGAGCAGGGGCATGGAGCTC-3’
SJ12d7 top	5’-CTGCAGGGTTTTTGTTCCAGTCTGTAGCACAGTGGTAGTACTCCACTGTCTGGCTGTACAAAAACCCTGCAG-3’
SJ12d7 bottom	5’-GACGTCCCAAAAACAAGGTCAGACATCGTGTCACCATCATGAGGTGACAGACCGACATGTTTTTGGGACGTC-3’
SJ23d9 top	5’-CTGCAGGGTTTTTGTTCCAGTCTGTCGCACTGTGCACAGTGGTAGTACTCCACTGTCTGGCTGTCTGCAG-3’
SJ23d9 bottom	5’-CTGCAGACAGCCAGACAGTGGAGTACTACCACTGTGCACAGTGCGACAGACTGGAACAAAAACCCTGCAG-3’
SJ12d9 top	5’-CTGCAGTCCAGTCTGTAGCACTGTGCACAGTGGTAGTACTCCACTGTCTGGCTGTACAAAAACCCTGCAG-3’
SJ12d9 bottom	5’-CTGCAGGGTTTTTGTACAGCCAGACAGTGGAGTACTACCACTGTGCACAGTGCTACAGACTGGACTGCAG-3’
12d7 top	5’-GATCTGGCCTGTCTTACTACAGACTGGAACAAAAACCCTGCAG-3’
12d7 bottom	5’-CTGCAGGGTTTTTGTTCCAGTCTGTAGCACTGTGTAAGACAGGCCAGATC-3’
23d9 top	5’-GATCTGGCCTGTCTTACACAGTGGTAGTACTCCACTGTCTGGCTGTGCGCTTATTCTGCAG-3’
23d9 bottom	5’-CTGCAGAATAAGCGCACAGCCAGACAGTGGAGTACTACCACTGTGTAAGACAGGCCAGATC-3’
LMO2 top	5’-GATCTGGCCTGTCTTACACAGT**A**GTAGTAGGCTGT**G**CAA**T**AA**TT**CTGCAG-3’
LMO2 bottom	5’-CTGCAG**AA**TT**A**TTG**C**ACAGCCTACTAC**T**ACTGTGTAAGACAGGCCAGATC-3’

Table S5: Oligonucleotides used in PCR assays, Related to Star Methods

Oligonucleotide	Sequence	Tm °C
JH290F	5'-GCACGACAGGTTTCCCGACTGG-3'	61
JH290R	5'-CCAGCTGAACGGTCTGGTTATAGGTAC-3'	61
23-RSS Cut Fwd	5'-CTGGCTAGCGTTTAAACTTAAGC-3'	60
23-RSS Cut Rev	5'-CTATGATCGATTCACTGACTGTAGA-3'	60
12-RSS Cut Fwd	5'-CCTGTGCTGGGAGACCTG-3'	60
12-RSS Cut Rev	5'-AGACGCCCGAATCTCACC-3'	60
Neo3 Fwd	5'-TGCTCCTGCCGAGAAAGTATC-3'	60
Neo3 Rev	5'-TTTCGCTTGGTGGTCGAATG-3'	60
SJ Cut Fwd	5'-CTGGCTAGCGTTTAAACTTAAGC-3'	60
SJ Cut Rev	5'-TGTTTTTCCAAAGCGGTCGAG-3'	60
Jdnorm Fwd	5'-GTCGCCTTCTTGCACATTACTC-3'	60
Jdnorm Rev	5'-AACAACAGATGGCTGGCAAC-3'	60
12-RSS virus Norm Fwd	5'-CCACCTTCCAGCAAGCTTG-3'	60
Virus Norm Rev	5'-CCCGTAGTTTTAGAAGGCACAG-3'	60
23-RSS virus Norm Fwd	5'-GGTACAGTGCAGGGGAAAGAATAG-3'	60
23-RSS virus Norm Rev	5'-GTGAATCGATCATAGTCTAGAGGG-3'	60
SJ virus Norm Fwd	5'-CGTCGCCTTCTTGCACATTAC-3'	60
FM25	5'-GCGGTGACTCGGGAGATCTGAAGTG-3'	-
FM11	5'-CACTTCAGATC-3'	-
CMV fwd	5'-CGCAAATGGGCGGTAGGCGTG-3'	57
12-RSS fwd	5'-CTTCCTGTGCTGGGAGACCT-3'	60
BGHRev	5'-TAGAAGGCACAGTCGAGG-3'	55
V1SacIF	5'-GCCAACTGGGTCCAAGAAAAAC-3'	58
J1StyIR	5'-GCACCTCAAGTCTTGGAGAG-3'	58
V1RSSF	5'-GGCACAGACTGAGGATGAG-3'	55
J1RSSR	5'-CAGTCAGTTTGGTTCCTCCA-3'	55
Vend2nd	5'-CCTGTTAAGAAGATGGTAGTTATGAGACTG	61

	TACC-3’	
Jend3	5’-CCTATGAGGACATATGGATCCTGGGAAGA AGG-3’	63
Jk5 end first	5’-CCTGTGCATCAATAGAAGATCC-3’	54
Jk5 end second	5’-CTTAGGCTTCTGAGACCAGT-3’	54
Jk5 cdjt first	5’-GAACTGACTTTAACTCCTAACATG-3’	53
Jk5 cdnd first	5’-GTACTTACGTTTCAGCTCCAGC-3’	56
Vk3 end first	5’-GTAATTTTTACGAATAAACCTATAGC-3’	50
Vk3 end second	5’-GAGCCTCAGTGAGCTGCAAC-3’	58
Vk3 cdnd first	5’-GAATATTATGGCACAAGTTTAATGCAG-3’	54
Vk3 cdnd second	5’-CCTCATCTATGCTGCATCCAACG-3’	58
Vk16 end first	5’-CATTCTCCATGGGTTCATGCT-3’	54
Vk16 end second	5’-GCCCAACATCTGGTTTCTGC-3’	56
Vk16 cdjt first	5’-GCCTGGTATCAAGAGAAACC-3’	54
Vk16 cdnd first	5’-GGATCCACTTTGCAATCTGG-3’	54
GAPDH F	5’-ACTTTCTTGTGCAGTGCCAGC-3’	56
GAPDH R2	5’-GCACACTTCGCACCAGCATC-3’	56
Vend1	5’-CAGACTAGGGAGATGTAGCCACCTG-3’	61
Jend1	5’-CCTATGAGGACATATGGATCCTGGGAAGA AGG-3’	63
Vendnested	5’-CTCGGGAGATCTGAAGTGCACAATG-3’	60
VendApaLI	5’-CGGGAGATCTGAAGTGTGCACAATG-3’	60
Jendnested	5’-CTCGGGAGATCTGAAGTGCACTGTG-3’	61
JendApaLI	5’-CTCGGGAGATCTGAAGTGTGCACTGTGATA T-3’	61
Genomic DNA PCR 680bp Fwd	5’-TCATTCCCAGTGTTGATGCC-3’	54
Genomic DNA PCR 680bp Rev	5’-TGGGTCCACTCTGAAGGAAC-3’	56
Genomic DNA qPCR 200bp Fwd	5’-ACCATGTCCTTCCCGATCTG-3’	56
Genomic DNA qPCR 200bp Rev	5’-TGGGTCCACTCTGAAGGAAC-3’	56

hROSA26-Bio primer	5'-/5Biosg/ TTCCTGGGTCAGGACGCTTC-3'	
hROSA26-I5-Nested	5'-ACACTCTTTCCCTACACGACGCTCTTCCGATCT**BARCODE**GGAACTGCCTACTCCACGTT-3'	
IgH-Bio primer	5'-/5Biosg/ GGAAGGTGGAGGCTCTGAGC-3'	
IgH-I5_Nested-control	5'-ACACTCTTTCCCTACACGACGCTCTTCCGATCT**BARCODE**ATGCTGCCACTTCTAGAGCA-3'	
12-RSS 1st round	5'-GATCACGAGACTAGCCTCG-3'	56
12-RSS 2nd round	5'-CTGTGCTGGGAGACCTGGGC-3'	62
23-RSS/SJ23d9-12-RSS 1st round	5'-GGAATTCCTGCAGCCCGTAG-3'	58
23-RSS 2nd round	5'CCTGCAGCCCGTAGTTTTAATACGACTCACTATAGGG-3'	65
SJ-12-RSS/SJ12d7-23-RSS 1st round	5'-GATCACGAGACTAGCCTCGAG-3'	58
SJ-12-RSS 2nd round	5'GCTAGCGTTTAAACTTAAGCTTGGTACCGAGCTCGG-3'	65
SJ-23-RSS 1st round	5'-AGTCGAGGCTGATCAGCGGG-3'	60
SJ-23-RSS 2nd round	5'-TGTTTTTCCAAAGCGGTCGAGCTGCAGGG -3'	64
1st round reverse (DR20)	5'-GCTATGTACTACCCGGGAATTCGTG-3'	59
Ligation primer (DR19)	5'-CACGAATTCCC -3'	
2nd round reverse	5'-GTACTACCCGGGAATTCGTGCACAG-3'	61

Table S6: Plasmids, Related to Star Methods

Plasmid	Description
**pEF-McR1**	The cDNA for core RAG1 (amino acids 384-1008) was cloned in frame with a maltose binding protein tag into the Xba I and Bam HI sites of pEF-XC (Mizushima and Nagata, 1990).
**pEF-McR2**	The cDNA for core RAG2 (amino acids 1-387) was cloned in frame with a maltose binding protein tag into the Xba I and Bam HI sites of pEF-XC (Mizushima and Nagata, 1990).
**pJH290**	A 12- and 23-RSS are cloned in a convergent orientation 329 bp apart (Steen et al., 1996; Han et al., 1999)
**pJH29012SJ + pJH29023SJ**	As above but where an SJ replaces one of the RSSs; pJH29012SJ thus has a 12-RSS and an SJ and pJH29023SJ has a 23-RSS and an SJ
**pSJ+**	Oligonucleotides containing a consensus SJ oligonucleotide with terminal Bam HI and Xho I sites were annealed and cloned into the polylinker of pcDNA3.1. An additional 120 bp Sal I/Bam HI fragment was cloned into the Xho I site of the polylinker to enable the SJ plasmid to be distinguished from the 12- and 23-RSS plasmids.
**p23+**	Oligonucleotides containing a consensus 23-RSS oligonucleotide with terminal Bam HI and Hind III sites were annealed and ligated into pcDNA3.1 cut with Bam HI and Hind III. To remove a cRSS in the polylinker of pcDNA3.1, the vector was digested with Eco RI and Xba I and the oligonucleotide sequence AATTCTCTACAGTCAGTGAATCGATCATAGT was ligated in to give a unique sequence that could be used for as a primer for PCR.
**p12+**	Oligonucleotides containing a consensus 12-RSS oligonucleotide with terminal Bam HI and Hind III sites ligated into the polylinker of pcDNA3.1. To remove a cRSS in the polylinker of pcDNA3.1, the vector was digested with Eco RI and Xba I and the oligonucleotide sequence

	AATTCGTCTCACATCGATGAACTATTGTATGT was ligated in to give a unique sequence that could be used for as a primer for PCR.
**pSJ12d7+ and pSJ23d7+**	The SJ sequence in pSJ was replaced with a mutant SJ sequence in which the heptamer in the 12-RSS or 23-RSS within the SJ was deleted (SJ12d7 and SJ23d7, respectively).
**pSJ12d9+ and pSJ23d9+**	The SJ sequence in pSJ was replaced with a mutant SJ sequence in which the nonamer in the 12-RSS or 23-RSS within the SJ was mutated (SJ12d9 and SJ23d9, respectively).
**pSJ12d9CAC+ and pSJ23d9CAC+**	As above but the CAC of the heptamer was additionally mutated to TAT
**pSJ12d923d9CAC+**	The RSSs in pSJ+ were mutated in both 12- and 23-nonamers and heptamers. The heptamer mutations changed CAC to TAT in each RSS. The nonamer mutations were as above.
**pJH548 (RAG1 expression vector)**	The full length mouse RAG1 cDNA was cloned into pCDM8 so that the cDNA was expressed via the CMV immediate early promoter (Sadofsky et al 1993).
**pEFflRAG2 (full length RAG2 expression vector)**	The full length mouse RAG2 cDNA was cloned into the pEF-XC vector (Mizushima and Nagata, 1990) so that the cDNA was expressed via the strong EF1α promoter.
**pEFcRAG2 (core RAG2 expression vector)**	Core mouse RAG2 cDNA (amino acids 1-387) was cloned into the pEF-XC vector (Mizushima and Nagata, 1990) so that the cDNA was expressed via the strong EF1α promoter.
**pWPI**	Second generation bicistonic lentiviral vector to allow for simultaneous expression of a transgene and EGFP marker. A gift from Didier Trono (Addgene plasmid #12254).
**pWPI-12RSS**	The 12-RSS sequence was amplified from p12+ using the T7 forward and bGH reverse primers. The PCR product was cloned into the Pme I site of pWPI.
**pWPI-23RSS**	As above but the 23-RSS was amplified from p23+.

**pWPI-SJ and pWPI-mutant SJ vectors**	As above but the SJ sequence was amplified from pSJ+ whilst the mutant SJ sequences were amplified from the respective mutant pSJ+ plasmids.
